# Supplementary material for: Effect of supplemental nutrition in pregnancy on offspring’s risk of cardiovascular disease in young adulthood: Long-term follow-up of a cluster trial from India
Source: PLoS Med. 2020 Jul 21;17(7):e1003183. doi: 10.1371/journal.pmed.1003183 (PMC7373266; doi:10.1371/journal.pmed.1003183)

APCAPS PROTOCOL

Andhra Pradesh Children And Parents Study (APCAPS)

Nutritional challenges, abdominal adiposity and type 2 diabetes in Indians:
Parental and offspring cardio-metabolic risk:
a trans-generational extension of Hyderabad Nutrition Trial

STUDY PROTOCOL

Version 2

Dated 1st January, 2011

CONTENTS

S No.	Particulars	Page No.
1.	Overview of Study	1
2.	Main Outcome Measures and Study Instruments	3
3.	Study Staff and their Responsibilities	5
4.	General Outline of fieldwork	8
5.	Protocol for Participant tracking	8
6.	Protocol for village clinic	10
7.	Protocol for Blood Sampling	15
8.	Protocol for Saliva Sampling	25
9.	Protocol for Clinic Questionnaire, including FFQ	27
10.	Protocol for Anthropometry	65
11.	Protocol for Blood Pressure	71
12.	Protocol for Spirometry	74
13.	Protocol for Accelerometer measurements	79
14.	Protocol for TANITA body composition measurements	82
15.	Protocol for Vascular Measurements	90
16.	Miscellaneous procedures at completion of clinic	100
17.	Inventory and care of Equipment	101
18.	Calibration of Instruments	102
19.	Data Entry and Data Quality Control	108
20.	APPENDIX	110

Overview of the study

Background**: Recent studies have shown associations between maternal and paternal measures and offspring's' health and disease risk in childhood.(1) Much of this so far limited evidence relates maternal birth size and childhood or adult height to outcomes in the offspring, suggesting a critical role for maternal nutritional and environmental influences on disease risk in the next generation (2-4). Studies linking maternal characteristics and offspring outcomes are however very limited as compared to the numerous studies relating birth size and outcomes within a generation. There has been little research in developing country settings on the intergenerational pathways between parental health and social well-being in childhood and offspring's health. With the current "epidemic" of chronic diseases in India, with increasingly younger populations being affected, it would be of importance to study the trans-generational association and transmission of chronic disease risk, if any, to the next generation.**

Study objectives:

1. To examine the association between parental and offspring cardio-metabolic risk profile and to see if the mother-offspring associations are different from father-offspring associations.
2. To study if parental deprivation in childhood (assessed from socio-economic data and height and leg length of the parents) is associated with cardio-metabolic risk in the offspring, and if so, whether nutritional supplementation of mothers in pregnancy attenuates this risk
3. To see if children born before the nutritional supplementation of mothers differ from those born after, in their disease risk profile.

Methods :

Setting: In the Hyderabad Nutrition Trial (1987-1990), a controlled community trial of nutritional supplementation of pregnant women and children< 6 years of age, integrated with other public health programs, over 2000 women in about 30 villages were randomised to receive/ not receive nutritional supplementation during pregnancy and the first five years of their child/children's life. The children born during this initial study were re-examined at 13 -16 years (SK) and again now at 18 - 21 years are being followed up in the Hyderabad DEXA study –" Nutritional challenges, abdominal adiposity and type 2 diabetes in Indians" for the effect of nutritional supplementation in early life on obesity, diabetes and heart disease in India.

The proposed Trans-generational study (APCAPS) in this group will look at the association between parental height and cardio-metabolic risk profile and risk markers in the offspring, where parental

height and maternal leg length in particular are used as proxy measures of adverse nutritional and socioeconomic conditions in their childhood.

Study plan: An initial enumeration exercise to account for the current available numbers of subjects in the birth cohort (F1) in both intervention and control groups of the Hyderabad Nutrition Trial will be carried out. A sampling list of these subjects will then be used to carry out a retracing exercise through home visits in the villages by trained field investigators to enumerate the numbers of parents (F0) alive in both groups. We will also attempt to record in this visit the number, age and date of birth of the siblings of the index subject/subjects in the Nutrition trial. (Appendix 1 -Tracking Form). Parents and siblings will then be invited to participate in the current study.

Examination: All participants will undergo an interview to record information on health, diet, nutrition and physical activity besides anthropometric measures, blood pressure, body composition measurements (TANITA) and pulmonary function assessment. Blood samples will be collected and analysed to measure glucose, insulin, cholesterol, triglycerides and liver function. Saliva samples will be collected and stored for future genetic studies. All subjects will also be invited to a screening examination at NIN where DXA scanning will be used to measure body fat and central-body fat. Non-invasive measures of coronary disease risk will be made.

Timetable: The fieldwork is expected to take 18 months to complete (Jan 2010 to June 2011), with a further 3 months to analyse the data and write-up the results (study end date October 2011).

Source of funding: The study is funded by the Wellcome Trust, UK, and coordinated jointly by the National Institute of Nutrition, India, University of Bristol and London School of Hygiene & Tropical Medicine, UK with technical and administrative support from the South Asian Network for Chronic Diseases (SANCD) and Centre for Chronic Disease Control (CCDC), New Delhi.

Table 1 . Main outcome measures and study instruments

Outcome	Instrument/method	Comments
**Diet & physical activity**		
Diet	Food frequency dquest. (single recording)	Recall over last year
Physical activity	Activity frequency quest. (single recording)	Recall over last week
Physical activity	Accelerometers (single reading)	Actigraph
**Anthropometry**		
Standing height	Portable stadiometer (two readings)	Seca Leicester height measure; at end expiration
Sitting height	Above, seated on a stool (two readings)	Local stool; above with feet dangling
Weight	Digital weighing machine (two readings)	Seca 899 portable digital scales; minimal clothing, no shoes
Waist circumference	ADE Metallic tape (two readings)	Narrowest part of waist observed from the front
Hip circumference	ADE Metallic tape (two readings)	Maximum extension of the buttock observed from the side
Mid-arm circumference	ADE Metallic tape (two readings)	Mid-upper arm on the left arm
Calf circumference	ADE Metallic tape (two readings)	Maximum calf circumference on the left leg
Head circumference<br>Chest circumference	ADE Metallic tape (two readings)<br>ADE Metallic tape (two readings)	Maximum head circumference<br>Maximum chest circumference at inspiration and expiration.
Triceps skinfold	Holtain skinfold calliper (three readings)	Same level as MAC; Left arm; 5 seconds delay for all skinfolds
Biceps skinfold	Holtain skinfold calliper (three readings)	1 cm superior to line marked for MAC, Left arm
Subscapular skinfold	Holtain skinfold calliper (three readings)	Just below inferior angle of scapula, 45 degree angle, Left side
Suprailiac skinfold	Holtain skinfold calliper (three readings)	Anterior Axillary line superior to iliac crest, 45 degree angle, Left side
Medial calf skinfold	Holtain skinfold calliper (three readings)	Medial side of left calf viewed from front, same level as calf circ.
Grip Strength	Lafayette Hand-held Dynamometer 78010 (four readings)	Right and left arm separately. Provides average of 4 readings.
Body Composition	TANITA BC418 M57NA<br>DEXA study	Body fat ratio, fat mass, fat-free mass, total body water, BMR and segmental body composition.<br>Whole body, spine and hip scans
**Vascular physiology**		
Blood pressure	Systolic & diastolic (three readings)	Omron HEM 7300 ; Sitting, right upper arm; small/medium/large cuffs
Pulse rate	Beats per minute (three readings)	Omron HEM 7300 ; Sitting, right upper arm; small/medium/large cuffs
Carotid Intima Media Thickness	Ultrasound (Ethiroli)	Supine measures taken of carotid artery

Pulse Wave Velocity<br>**Outcome**	Vicorder (three readings)<br>**Instrument / Method**	Supine<br>**Comments**
**Lung function tests**		
Forced vital capacity	Card Guard Spiro Pro (min three readings)	Three acceptable blows – maximum eight blows
Forced expiratory volume$_1$	Card Guard Spiro Pro (min three readings)	Three acceptable blows – maximum eight blows
Room temperature	Digital thermometer (single reading)	MEXTECH TM1
**Biochemistry**		
Haemoglobin	Calorimetry	At NIN
Fasting sugar	Randox kit/Autoanalyser	At NIN
Fasting triglycerides	Randox kit/Autoanalyser	At CCDC/SANCD, New Delhi
Total cholesterol	Randox kit/Autoanalyser	At CCDC/SANCD, New Delhi
HDL-cholesterol	Randox kit/Autoanalyser	At CCDC/SANCD, New Delhi
Fasting insulin	Radioimmunoassay	At CCDC/SANCD, New Delhi
Liver function tests	Randox kit/Autoanalyser	At CCDC/SANCD, New Delhi
Serum Calcium	Randox kit/Autoanalyser	At CCDC/SANCD, New Delhi
Serum Phosphorus	Randox kit/Autoanalyser	At CCDC/SANCD, New Delhi
Serum Alkaline Phosphatase	Randox kit/Autoanalyser	At CCDC/SANCD, New Delhi
Serum Creatinine	Randox kit/Autoanalyser	At CCDC/SANCD, New Delhi
CRP	Randox kit/Autoanalyser	At CCDC/SANCD, New Delhi
Saliva sample	Oragene kits	At CCDC/SANCD, New Delhi

Study Staff and their Responsibilities

The team will consist of Project Manager, Field manager, three SRFs, Fourteen fieldworkers, three biochemists, one DXA technician (NIN staff member), two data entry operators, and four clinic helpers.

Project Manager

- Overall responsibility for the performance of the team
- Deliver recruitment targets
- Assist with training of staff
- Ensure data quality and completeness
- Coordinate day-to-day running of the study (plan activities of other staff, surveys, and clinics)
- Supervise data entry, data back-up, data storage (electronic and paper), data validation and verification
- Supervise record keeping
- Procure equipment and consumables on time, and ensure their maintenance (except for DXA scanner)
- Develop links with the village leaders and Anganwadi staff.
- Report to the local PI at NIN and PI at Bristol.

Field Manager

- Overall responsibility for day-to-day management of field work and teams and coordinate smooth functioning of all components of fieldwork
- Ensure recruitment targets
- Primarily responsible for establishing contact with the Village leaders / Panchayat officials, securing permissions as appropriate and earmarking clinic site towards end of work in previous site. This should allow smooth moving over of the team from site to site without loss of time between villages.
- Conduct household visits with the field staff and motivate participation of subjects.
- Ensure daily calibration of equipment and appropriate measures when required
- Ensure data quality, completeness and accuracy
- Responsible for checking all equipment are in working condition and take suitable action when required
- Keep an inventory of all consumables and procure all requisite material on time
- Daily mechanism of reporting fieldwork to the Project Manager and together working on any logistic issues in the field work, including co-ordinating staff suitably in case of absenteeism so work is not affected in the field.
- Report to the local PI and PI at Bristol.

Medical officer (Senior research fellow) At NIN

- Complete the medical proforma, where incomplete**.**
- Measure carotid intima media thickness.
- Measure augmentation index and pulse wave velocity when the fieldworker is not available.
- Assist in DEXA studies, where necessary.

- Communicate results of medical examination (blood pressure) and offer advice to the participants
- Manage any medical emergencies or referrals that may be required
- Accompany the field team in case need arises, as when Field doctor is absent.

Field Medical officer (Senior research fellow)

- Accompany the field team to the village clinics
- Complete the medical proforma**.**
- Measure Blood Pressure.
- Collect/assist in blood sample collection in case biochemist has difficulty or is unavailable
- Assist in Pulmonary Function Tests, where necessary.
- Communicate results of medical examination (blood pressure, Hemoglobin and fasting glucose) and offer advice to the participants
- Manage any medical emergencies or referrals that may be required
- Overall assistance in field clinic for other measurements.

Fieldworkers (X12)

- Arrange appointments for participants
- Explain nature of study to participants
- Provide information sheets to participants
- Send reminders to participants
- Organise clinics, maintain equipment and records
- Involved in all aspects of the fieldwork including questionnaire completion, anthropometry, blood pressure, body composition (TANITA) and Pulmonary function measurement.
- Collect participants and transport to NIN and back for NIN component of study.
- Report to the Project Manager
- Along with the field manager and village helper, will be responsible for household visits and motivation of subjects to attend the clinics.

Fieldworkers (X2)

- Collect participants and transport to NIN and back for NIN component of study.
- Measure augmentation index and pulse wave velocity
- Involve in all aspects of the fieldwork including questionnaire completion, anthropometry, blood pressure, body composition (TANITA) and Pulmonary function measurement if needed.
- Report to the Project Manager

Field Biochemist (2)

- Accompany the field team to village clinics, collect blood samples, centrifuge in the field and ensure timely and accurate processing/analyses of samples
- Analyse for glucose and haemoglobin if the NIN Biochemist is not available
- Maintain equipment and necessary supplies of consumables
- Assist biochemist at NIN in arranging transport of samples and quality control with the coordinating laboratory in Delhi
- Report to the Project Manager and Dr Ruby Gupta/ Dr. Vipin Gupta in Delhi

Biochemist NIN (1)

- Store the samples collected in the field with the help of Field Biochemist and clinic helper
- Analyse for glucose and haemoglobin on the same day.
- Give the results to the Field Manager.
- Maintain equipment and necessary supplies of consumables
- Arrange transport of samples and sample dispatch sheets and quality control with the coordinating laboratory in Delhi
- Report to the Project Manager and Dr Ruby Gupta/ Dr. Vipin Gupta in Delhi

DXA technician (NIN staff)

- Conduct whole body, lumbar spine, and hip scan.
- Conduct analyses to estimate adiposity and abdominal adiposity from whole body scan
- Report to the Project Manager and local PI

Data Manager (2)

- Data entry and check data completion, maintain databases
- Enter data from DXA and TANITA printouts into database
- Enter census codes on the questionnaires using special software
- Generate and print labels and lists for fieldwork.
- Maintain records and paperwork, progress spreadsheets, electronically transmit the required files at timely intervals
- Report to the Project Manager.

Field Clinic helpers (2)

- Set up clinics, instruments and organise the flow of participants and arrange refreshments
- Help and support the activities of the team

NIN Clinic helpers (2)

- Set up clinics, instruments and organise the flow of participants and arrange refreshments.
- Help the DXA technician during the scans.
- Help and support the activities of the team
- Help in cleaning the lab glassware.

General Outline of Plan for Fieldwork and Data Collection

The APCAPS work will involve the conduct of clinics in each of 29 villages which formed the base of studies conducted since 1987 – 90, as part of the Hyderabad Nutrition Trial. Children from these study households who were enrolled in the original study are currently being followed up as part of the Hyderabad DEXA study. They form the index subjects. Parents and siblings of these index subjects will be invited to participate in the APCAP study, along with the DEXA subjects.

After carrying out a household –level survey to track the availability of household members, a detailed action plan will be charted out to conduct clinics in each of these villages. Preliminary visits to establish a rapport with the subjects and communicate with the Village Head/ Gram Panchayat and obtain his/her/their permission to conduct the clinics in a suitable, convenient and centrally located site will be done by the Field Manager and field workers.

These subjects will also undergo DEXA studies for body composition and vascular studies at the NIN location.

A detailed protocol is set out for each phase of the study - Tracking exercise, Clinics, NIN arm of study and transport of biochemical samples.

Protocol for Participant Tracking:

1. The Team Leader plans the village to be visited each day, alternating between Intervention and Control sites.
2. A list of names and addresses of the Hyderabad DEXA study subjects in each village is prepared by the Field Manager. Appropriate labels containing the Historical ID (Family ID) and village ID are placed on the tracking form.
3. A team of 4 Field workers will conduct a household-level survey in all study villages using the available sampling list.
4. The field staff visits each subject's household in the designated village and records information about the parents and siblings in the Tracking Form. This includes the names, addresses, current status (dead, alive, moved) of parents and details of birth, including-name, date of birth, gender and status of siblings. They will also use this opportunity to explain to them the nature and purpose of the tentative study and clinics to be carried out in the near future in the villages.
5. A brief meeting is conducted with the Village head / Panchayat leader to request permission to conduct the clinic at the Panchayat office premises/village school/ other appropriate site in the village.
6. An attempt will also be made to enlist support of the Anganwadi teacher and helper in mobilising support for the study. Where appropriate, help is sought from one of the index subjects in the village to garner support and enthusiasm for participation from his/her own and other families.
7. The field staff revisits people not available at previous visits to complete all information on every household.

8. Fieldworker continues down the list until everyone in the village who is eligible has been visited, and has either been contacted or refused participation or cannot be identified.
9. The information collected from this exercise is entered into a spreadsheet and a weekly count of villages covered with number of households and status sheet of participants available in each household for the APCAPS is prepared.
10. This information will then be used to generate a participant list for the APCAPS.

Participant Information Sheet - APPENDIX 2

At the time of first contact, the fieldworker reads the participant information sheet (translated into Telugu) to the participant and answers any questions.

The fieldworker makes sure he has covered all the points in the checklist for participation.

Benefits of the study (to be communicated to the potential participant by the Field staff at first contact, and while inviting to clinic for participation).

Tests will be conducted for the following, free of cost:

- Blood pressure
- DXA scan to measure body fat
- Heart and blood vessel function (Augmentation Index, Pulse Wave Velocity, Carotid Intima Media thickness)
- Lung function
- Blood tests, including glucose

You will be told if a problem is detected and will be given your blood tests

For examination, please remember the following:

- Be on time for the clinic.
- You will be reimbursed for your time and food is provided
- Come fasting overnight, unless you are pregnant or diabetic
- Avoid metal on clothes. Preferably do not wear gold or silver jewellery
- No calcium for 2 days before hand
- Avoid alcohol and fatty food one day before
- Do not smoke from the evening before the examination
- For women, come within 10 days of last menstrual period unless ceased childbearing
- Bring proof of ID-household ration card, voter ID or other
- Bring any medication that you use

Informed consent. Completed by research assistants – APPENDIX 3

One of the field workers who ever is the field in charge for that village, will explains the study using the prepared information sheet and takes consent. He/she must make sure that the participants have been able to ask any questions or address any concerns. The Telugu versions are also available for those subjects who prefer to sign this version.

Protocol for Village Clinic:

1. Village clinics will be carried out alternately in the intervention and control villages so as to cover all households in the list in each village selected for study at a time.
2. Appointments will be given in the selected village to about 6 households using the participant list generated by the Field Manager / Field-in-charge. The parents and all siblings of the index subject from the DEXA study will be invited to participate in APCAPS, along with the index DEXA subject.
3. Inclusion Criteria: All mothers who participated in the Hyderabad Nutritional Trial in either Intervention or Control arm, their spouses and all children born to them, will be included in the study.
4. Exclusion Criteria: There are no overall exclusion criteria as all the members of the family are invited to participate. Only in some cases as outlined below, some participants will be excluded for some components of the study.
    i. Subjects with structural limb / spinal deformities will be excluded from the anthropometry protocol, but encouraged to complete other sections of the protocol.
    ii. Children below 10 years will be excluded from blood sampling protocol.
    iii. Subjects who are mentally disabled, Deaf & Dumb can be excluded from Questionnaire, but encouraged to complete other sections of the protocol with the help of a family attendant.
    iv. Pregnant women will be excluded from the DEXA examination.
5. The field staff will visit them 2-3 days before, explain the nature and purpose of the study and hand out the Participant information sheet. Any questions the subjects may have at this point are answered. Appropriate instructions for fasting overnight are given. A log book will be also maintained and the fieldworker records information on how many subjects were contacted and whether they have agreed to attend. Reasons for non-attendance and refusal are also noted.
6. The field staff will also enlist the help of the Anganwadi teacher and village helper to remind the subjects of the clinic in the village.
7. The Field Manager or DEO supervises and prepares all the questionnaire folders, sampling kits, etc with appropriate labels that have been printed with barcodes and sent from the SANCD office by Dr. Vipin Gupta / Ms. Gagandeep in New Delhi.
8. On the morning of the clinic, the team consisting of the Field Manager, one Medical Officer (SRF), 6 Field workers, one Biochemist and the clinic helper will leave in the morning with all necessary equipment from the Project office at NIN. A checklist of equipment to be transported for the clinics is maintained and checked before departure.

9. On arrival at the clinic site in the village, the equipment is set up by the clinic helper and the field staff and daily calibrations are carried out.
10. The biochemist sets up the field centrifuge and ensures sampling kits are in place and ready.
11. The clinic helper meanwhile also ensures that the contact person in the village has arrived with the subjects for the day. He will visit the households if necessary to remind the subjects scheduled for the day. The Field Manager and one Field –in-charge will also conduct household visits to motivate participation at the beginning and end of each day.
12. All subjects who attend the clinic are then requested to sign the Consent Forms after ensuring all information in the Participant information sheet is understood and any outstanding questions are answered.
13. The clinic helper arranges for breakfast for all subjects in the meantime.
14. Height, weight, blood pressures are recorded. After ensuring the subjects have fasted overnight, the biochemical and saliva samples are collected. The biochemist then centrifuges the samples while the saliva samples are stored in the ice box. In the event that there is a power shutdown, either a generator is used to ensure continuous supply or the biochemist will have to return to NIN to process the samples and carry out analyses of HB and fasting glucose. The biochemist will ensure the proper storage of samples in the freezers at NIN. A set protocol will be followed for regular transfer of samples to the Central lab at SANCD in Delhi. Where subjects refuse blood sampling, an attempt at explaining and reassurance is tried but no subject will be pressurised for the same. Instead, he/she is encouraged to complete other measurements and the questionnaire.
15. Body composition measurements using TANITA are carried out. Subjects are then provided breakfast. They will then move sequentially through the process of answering the questionnaire, undergo remaining anthropometry measurements and pulmonary function tests.
16. Accelerometers for recording Physical activity are also given to the subjects and appropriate instructions are provided about use and precautions.
17. At the completion of all requisite tests, a check is made to confirm completion of the questionnaire and all procedures.
18. The participant is then thanked for their time and participation, reimbursement is provided and appropriate vouchers with signatures are retained for the same. The ID proof is checked, details to be noted such as school registration / voter ID/ pan card / ration card number in the questionnaire. The ID proof will then be scanned electronically using a scanner and the jpeg image will be saved under the subject ID. If two or more subjects from the same family have a single ID, duplicate copies of the scanned image will be made and renamed with the respective subject ID.
19. Appointments are provided as and when possible for the subjects to travel to NIN for the DEXA and vascular studies.
20. When all the subjects have been covered for the day, the field staff checks the questionnaires, files the folders and the clinic helper dismantles and stores all the equipment in the vehicle for return to NIN. Where possible and if a safe and secure location is available, anthropometry and TANITA equipment as well as the centrifuge and generator are stored in the site of the village clinic for use during the remaining clinics in the village.

21. The Field Workers with the help of the Field Manager and Field-in-charge prepare the participant list and folders for the next day. The Biochemist prepares the vene puncture material, sampling kits with appropriate labels and transport box for the next day.
22. Appropriate phone calls and reminders are made to subjects attending the clinic next day. One phone call is always made to the Anganwadi Teacher/helper/contact person identified in the village who will then ensure attendance at the clinic in the morning.
23. The Field Manager will spend much of the day planning and organising fieldwork and ensuring overall smooth running of the fieldwork, participant attendance, data collection and complete measurements.
24. The Database person (DEO) will assist and support the Project Manager, manage the database, and enter data simultaneously as far as possible with the data collection.
25. A smooth mechanism for data checking, and transfer to the data manager at Bristol needs to be in place once data entry actually begins.

Exclusion of pregnant women from DXA scanning

People are exposed to a very low dose of radiation when they undergo a DXA scan. This means that we must ensure that pregnant women do not undergo DXA scanning.

Steps to ensure pregnant women are not screened using DXA.

1) Fieldworker visits female subjects together with Anganwadi worker two weeks before planned appointment.
2) Anganwadi worker asks subject about last menstrual period and regularity of period and estimated start of next menstrual period.
3) Female is given tentative appointment for 1-10 days after next menstrual period
4) One day before the appointment the female is visited by fieldworker and Anganwadi worker to ask about the start of the last menstrual period. If last menstrual period was not in the last 9 days, they are given a new appointment.
5) Women who are pregnant are given appointments for other components of the study in the same ways as the men, but are not given DXA scans.

Blood Sampling

Blood samples will be taken by the biochemist. The Medical officer will assist / help the biochemist whenever required. For all subjects a fasting sample will be taken. **Pregnant women and people with diabetes will not be asked to fast, but blood samples will still be taken. Blood samples will not be taken for children less than 10 years old.**
Answers to the following questions are recorded before the blood sampling.

SECTION B: Blood Sampling

	Blood sampling		
**2.1**	Any illness within the last week?	☐ [1=Yes; 2=No]	
**2.2**	If yes, specify what illness: Cold ☐ Cough ☐ Headache ☐ Fever ☐ Body aches ☐<br>Pain Abdomen ☐ Diarrhoea ☐ Vomiting ☐ Others ☐ Specify_____		
**2.3**	(a) Was this illness or some other reason responsible for reduction in food intake over the last week?	☐ 1=No reduction<br>2=Minor reduction<br>3=Major reduction	
	(b) Do you have diabetes?	☐ [1=Yes; 2=No]	
	(c) Are you pregnant?	☐ [1=Yes; 2=No]	
**2.4**	Day of last meal	☐ [1=Today; 2=Yesterday]	
**2.5**	Time of last meal	☐☐ : ☐☐ [Hours: minutes; 24-hour clock]	
**2.6**	Time blood sample taken:	☐☐ : ☐☐ [Hours: minutes; 24-hour clock]	
**2.7**	Saliva sample taken	☐ [1=Yes; 2=No]	
**2.8**			
**2.9**			
	**Success in blood sampling**	**(a) Volume**	**(b) Clot formation**
**2.10**	Red capped tube	☐ [1=No; 2=Partial; 3=Complete]	☐ [1=Yes; 2=No]
**2.11**	Purple capped tube 1	☐ [1=No; 2=Partial; 3=Complete]	☐ [1=Yes; 2=No]
**2.12**	Grey capped tube	☐ [1=No; 2=Partial; 3=Complete]	☐ [1=Yes; 2=No]
**2.13**	Purple capped tube 2	☐ [1=No; 2=Partial; 3=Complete]	☐ [1=Yes; 2=No]
**2.15**	(a) Any other comments on blood sample	☐ [1=Yes; 2=No]	
	(b) If yes, specify		

Labelling

Bar code labels will be used to label all the sample tubes, storage vials, boxes, questionnaire and all the paper documents of the project. Labels with barcodes will be printed in SANCD, Delhi and will be dispatched to NIN, Hyderabad. Kindly intimate the SANCD staff well in advance so that labels could be printed and posted well in advance to avoid last hour rushes and back logs.

Contact Person: Dr. Vipin Gupta (Mobile No. 09899119242)

SANCD, New Delhi

As soon as the subject will arrive in the field clinic, the subject ID will be recognized from the village list and the corresponding labels will be identified. The box sheets, report forms and reimbursement sheets need not be labeled. There will be 24 labels for each subject in the following sequence:

**Type**	**Number**	**Purpose**
General (APCAPS)	12	Questionnaire, 4 Vacutainers, Daily Worksheet, Dispatch Sheet, NIN Record Sheet
Serum (S)	3	Storage Vials for serum aliquots aspirated from 4 ml red capped plain vacutainer
Plasma(P)	3	Storage Vials for fluoride plasma aliquots aspirated from 2 ml grey capped vacutainer
E-Plasma (EP)	3	Storage Vials for EDTA plasma aliquots aspirated from 3 ml and 6 ml purple capped vacutainers
Saliva (SL)	3	Saliva tube
**Total**	**24**	

Protocol for Blood sampling

1. Preparation of Subjects:
    - Inform your subject at least two days before about sample collection.
    - To come after 10-12 hours of fasting.
    - Inform the 'Place' and 'Venue' of sample collection

2. Prepare kits with the following contents for sampling:
    - Vacutainers: **1** Red capped **1** Grey capped and **2** Purple capped (All vacutainers should be labelled)
    - Accessories: Tourniquet, Needle, Holder, Swab.
    - Pasteur pipette
    - Storage vials (9 in nos.) (labelled)

3. Items not provided in the kit but required for sampling
    - Centrifuge machine
    - Test-tube stand
    - Gloves
    - Needle destroyer & bio-waste disposal packets

4. Preparation for transportation and Storage
    - Ice-bucket
    - Ice-packs (Keep overnight in deep freezer or in the ice compartment of the normal refrigerator)

5. Enter the id no., Name, Age, Sex and Date in a logbook.

Blood sample collection

- Draw blood from the subject in the recumbent position, where possible. Make the subject sit for 10 min. before starting sample collection.
- Keep ready one holder, one needle, one red-capped vacutainer, one grey-capped vacutainer and two purple-capped vacutainers.
- Attach needle to the holder.
- After applying the tourniquet insert the needle into the vein. **(Avoid application of tourniquet for more than one minute)**
- Insert red-capped vacutainer. Collect blood up to the capacity.
- Take out the red-capped vacutainer and insert grey-capped vacutainer. Ensure that the tube is filled to the mark.

- Take out the grey-capped vacutainer and insert purple-capped vacutainers (2). Ensure that the tube is filled to the mark.
- Mix grey capped vacutainer (containing fluoride) and purple capped vacutainer (containing EDTA) by inversion for 7-8 times **(Do not shake vigorously)** and keep them in an ice- bucket (at 4 degree temperature).
- Allow the blood in red-capped vacutainer (Plain tube) to stand for 30 min at room temperature and allow blood to clot. Centrifuge the tube at **3500 rpm for 15 min**.
- Hemoglobin estimations to be performed simultaneously with fresh blood from EDTA vacutainer.

It is important that serum is separated as soon as possible and at no cost later than 45 minutes after blood collection.

- Aspirate the serum using the given plastic pipette into three labelled storage vials provided (Vials labelled as 'S' for serum)
- Similarly centrifuge the grey-capped vacutainers for **15 minutes at 3500 rpm**. Aspirate the plasma and transfer into three labelled vials provided in the kit (Vials labelled as 'P' for Plasma)
- **Ensure that the storage vials are closed tightly.**
- Do not centrifuge the purple-capped vacutainer.
- Mix well by inversion and Estimate Hemoglobin
- Centrifuge the tubes at **3500 rpm for 15 min**
- Transfer EDTA plasma into two storage vials labelled 'EP'.
- The storage vials (all 'S', 'P'& 'EP') and packed cells in purple tube are then transported in ice bucket to the Lab at NIN.
- Transfer three labelled 'S', two labelled 'P' and three labelled 'EP' vials to –20 ^{0} C deep freezer immediately. (To be despatched to the coordinating lab)

One vial of 'P' should be used for glucose estimation without much delay.

- **Needle should be destroyed by needle destroyer and vacutainers collected as bio-waste material should be sent to incinerator.**

Storage

Daily Activity

Properly labeled vacutainers and storage vials should reach NIN, Hyderabad at the end of each field and should be stored at -20°C in the deep freezer. The labelled questionnaire should be stored at the A-7 flat systematically in the order of subject recruitment and data manager should be well versed with their storage sequence. After data entry the questionnaires should be stored back in the same order. The storage vial containing fluoride plasma aliquot which is used for estimating fasting glucose level should be stored back in the deep freezer in the appropriate storage box along with its other aliquots after use

as soon as possible. The location of storage vials in the boxes should be recorded and stored with the lab and field technician. Field manager will be responsible to overview and supervise all these daily activities from time to time. The village leader (FW) will be responsible to report the total number of samples collected on each day to the field manager and confirm the completion of the daily activities mentioned above.

At the end of completing one village

After the field work of one village is complete (after reaching atleast 80% response rate), all the questionnaires and samples (saliva tubes, vacutainers, storage vial) will be finally arranged in such a manner that samples can be transferred on dry ice to SANCD, New Delhi via reliable courier service. The exact procedure given below should be followed to arrange the samples of a village. Field manager and project manager will be responsible to supervise and conduct these activities to ensure the completeness and precision of the sample information.

Questionnaires

After the completion of one village (data entry should continue simultaneously), all the questionnaires will be arranged serially (in the order of family ID) by the data manager along with the NIN helper under the supervision of field manager and project manager.

Confirm the subject ID applied on the questionnaires using the information from the village list, reimbursement sheet and daily worksheets (made by field technician and data manager) to check any missing questionnaires.

Separate all the questionnaires into four lots based on the subject type.

After separating all the questionnaires of DXA index children (subject ID starting with 1), their sibs (subject ID starting with 3), the mothers (subject ID starting with 5) and the fathers (subject ID starting with 6); arrange them in increasing order of their subject ID's.

After the four lots are arranged serially, all the questionnaires will be arranged according to the village list, i.e., in order of their family Id in the order of father, mother, sibs and DXA subjects. This step will be performed after insuring the completeness of data in each questionnaire.

All the subject ID (in the order stated in step 3) will then be noted on the dispatch sheets to make a record of total number of subjects covered on the village along with their other essential details.

Vacutainers

Once the serum is separated, the red capped plain 4 ml vacutainers can be discarded at NIN in appropriate manner. After the completion of one village, the lab technician will arrange and pack all the grey and purple capped vacutainers with the help of field technician, field helper and other field workers

under the supervision of field manager and project coordinator. The grey capped 2 ml sodium fluoride coated vacuatiners containing whole blood will be permanently stored in NIN while the purple capped 3ml and 6 ml EDTA coated vacutainers containing whole blood will be transferred to SANCD, New Delhi.

Separate all the vacutainers in four lots based on the subject type. All the grey and purple capped vacutainers of one individual or subject should be kept together.

After separating all the vacutainers of DXA index children (subject ID starting with 1), their sibs (subject ID starting with 3), the mothers (subject ID starting with 5) and the fathers (subject ID starting with 6); arrange them in increasing order of their subject ID's.

After the four lots are arranged serially on the thermocol stands, all the purple capped vacutainers of the members of one family will be tied together with the rubber band and will be then kept in a transparent plastic lock bags. Keep the list of the village handy to confirm the subject ID's of the members of each family.

While tying with the rubber band, note the missing vacutainers and other comments regarding the purple capped vacutainers in the appropriate column of the dispatch sheet.

Repeat steps 3 and 4 for each family, such that different bundles representing families are made and kept in the plastic bags.

Once the plastic bag is full note down the village name, date of packing, packet number and the list of family ID of the vacutainers kept in that bag on a sheet of paper and place it inside the plastic bag such that information is visible from outside.

Lock the plastic bag and pack it with the cello tape. Take care not to over full the plastic bags to avoid its tearing.

Repeat Steps 3-6, till all the purple capped vacutainers are packed.

Make a record of total number of purple capped vacutainers of a village.

Repeat the same procedure (steps 3 to 9) for the grey capped vacutainers. The missing vacutainers and other comments regarding the grey capped vacutainers will be recorded in the NIN record sheet while repeating step 4.

Storage Vials

After the completion of one village, the lab technician will arrange and pack all the storage vials in the storage boxes with the help of field technician, field helper and other field workers under the supervision of field manager and project coordinator. Two storage vials each of serum, fluoride plasma and EDTA plasma will have to be transferred to SANCD, New Delhi and one storage vial each of serum, fluoride plasma and EDTA plasma will be stored permanently at NIN, Hyderabad in the deep freezer.

Information regarding the location of each subject's storage vials should be with both the field and lab technician as the vials will be stored inside the boxes in the order of date of collection.

The storage vials (in the order of 'S', 'P' and 'EP') will be rearranged according to the increasing order of family ID by consulting the village list such that members of one family are stored together (father, mother, sibs, DXA subject) as done in case of saliva tubes and vacutainers.

Three aliquots will be permanently stored in three different boxes; such that each box has only one storage vial of serum, fluoride plasma and EDTA plasma of an individual. Out of these, two boxes will be transferred to SANCD, New Delhi and remaining one box will be permanently stored in NIN, Hyderabad.

The fluoride plasma aliquot used for fasting glucose estimates will be kept back in the NIN storage box.

Note down the details of the samples on the box sheet along with the volumes with the help of previous records given by field technician as soon as the storage box is full.

Label the box properly as NIN or SANCD box along with the village name and box number with the help of adhesive tape and cello tape.

Simultaneously, record any missing information and other comments in the appropriate columns of dispatch sheet and NIN record sheet.

Record the total number of boxes to be transferred to SANCD, New Delhi and also to be permanently stored at NIN, Hyderabad.

FLOW CHART

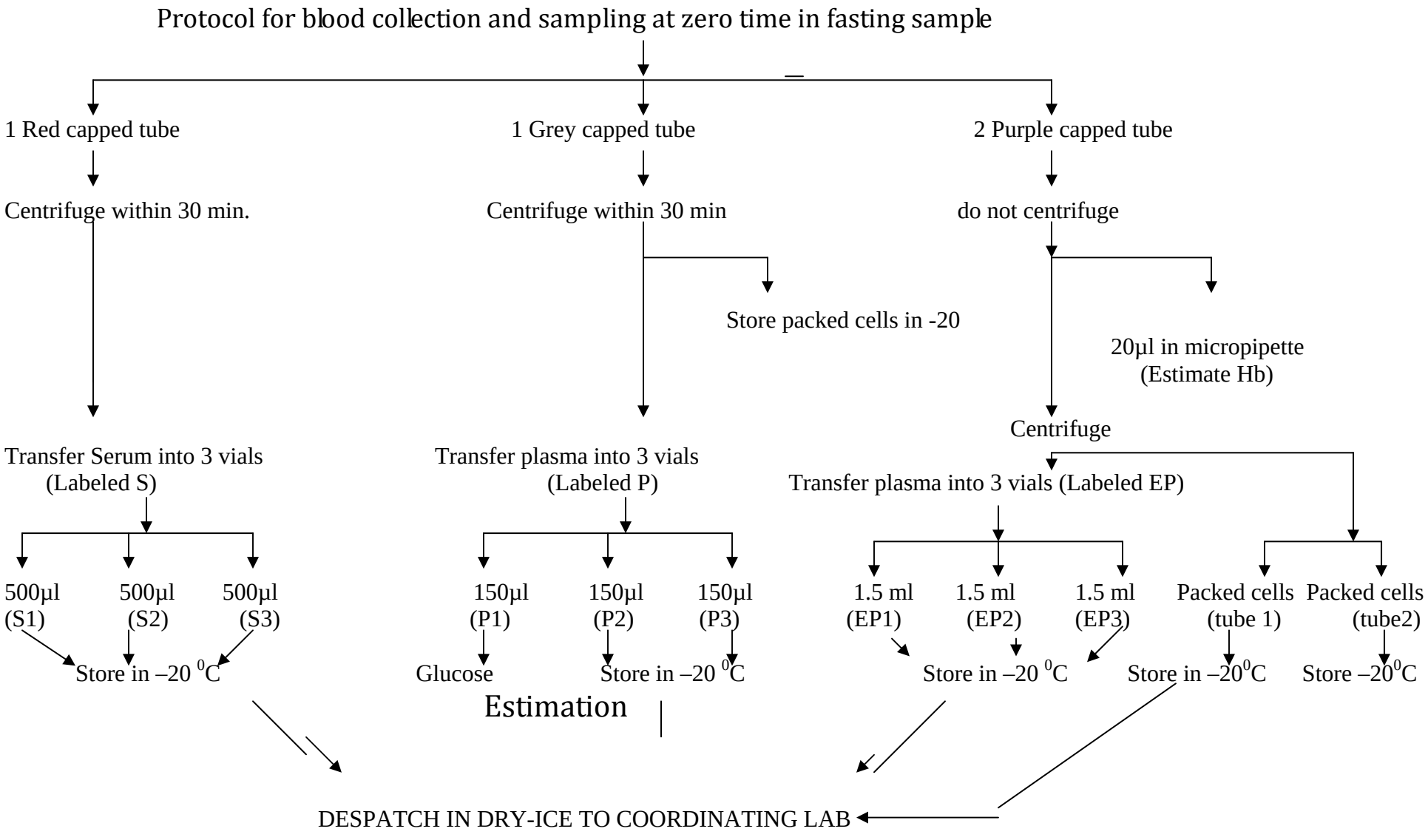
Protocol for blood collection and sampling at zero time in fasting sample
1 Red capped tube
1 Grey capped tube
2 Purple capped tube
Centrifuge within 30 min.
Centrifuge within 30 min
do not centrifuge
Store packed cells in -20
20µl in micropipette
(Estimate Hb)
Centrifuge
Transfer Serum into 3 vials
(Labeled S)
Transfer plasma into 3 vials
(Labeled P)
Transfer plasma into 3 vials (Labeled EP)
500µl (S1)
500µl (S2)
500µl (S3)
150µl (P1)
150µl (P2)
150µl (P3)
1.5 ml (EP1)
1.5 ml (EP2)
1.5 ml (EP3)
Packed cells (tube 1)
Packed cells (tube2)
Store in –20 0C
Glucose
Store in –20 0C
Store in –20 0C
Store in –200C
Store –200C
Estimation
DESPATCH IN DRY-ICE TO COORDINATING LAB

Name of the Village: **Date of Transfer:**

NIN Box No. **Total no. of Boxes:**

	1 S1	2 P1	3 EP1	4 S1	5 P1	6 EP1	7 S1	8 P1	9 EP1
Subject ID									
Volume									
	10 S1	11 P1	12 EP1	13 S1	14 P1	15 EP1	16 S1	17 P1	18 EP1
Subject ID									
Volume									
	19 S1	20 P1	21 EP1	22 S1	23 P1	24 EP1	25 S1	26 P1	27 EP1
Subject ID									
Volume									
	28 S1	29 P1	30 EP1	31 S1	32 P1	33 EP1	34 S1	35 P1	36 EP1
Subject ID									
Volume									
	37 S1	38 P1	39 EP1	40 S1	41 P1	42 E P1	43 S1	44 P1	45 EP1
Subject ID									
Volume									
	46 S1	47 P1	48 EP1	49 S1	50 P1	51 EP1	52 S1	53 P1	54 EP1
Subject ID									
Volume									
	55 S1	56 P1	57 EP1	58 S1	59 P1	60 EP1	61 S1	62 P1	63 EP1
Subject ID									
Volume									
	64 S1	65 P1	66 EP1	67 S1	68 P1	69 EP1	70 S1	71 P1	72 EP1
Subject ID									
Volume									
	73 S1	74 P1	75 EP1	76 S1	77 P1	78 EP1	79 S1	80 P1	81 EP1
Subject ID									
Volume									

Signature:

Name of the Village: **Date of Transfer:**

SANCD Box No. **Total no. of Boxes:**

	1 S2	2 P2	3 EP2	4 S2	5 P2	6 EP2	7 S2	8 P2	9 EP2
Subject ID									
Volume									
	10 S2	11 P2	12 EP2	13 S2	14 P2	15 EP2	16 S2	17 P2	18 EP2
Subject ID									
Volume									
	19 S2	20 P2	21 EP2	22 S2	23 P2	24 EP2	25 S2	26 P2	27 EP2
Subject ID									
Volume									
	28 S2	29 P2	30 EP2	31 S2	32 P2	33 EP2	34 S2	35 P2	36 EP2
Subject ID									
Volume									
	37 S2	38 P2	39 EP2	40 S2	41 P2	42 E P2	43 S2	44 P2	45 EP2
Subject ID									
Volume									
	46 S2	47 P2	48 EP2	49 S2	50 P2	51 EP2	52 S2	53 P2	54 EP2
Subject ID									
Volume									
	55 S2	56 P2	57 EP2	58 S2	59 P2	60 EP2	61 S2	62 P2	63 EP2
Subject ID									
Volume									
	64 S2	65 P2	66 EP2	67 S2	68 P2	69 EP2	70 S2	71 P2	72 EP2
Subject ID									
Volume									
	73 S2	74 P2	75 EP2	76 S2	77 P2	78 EP2	79 S2	80 P2	81 EP2
Subject ID									
Volume									

Signature:

Name of the Village: **Date of Transfer:**

SANCD Box No. **Total no. of Boxes:**

	1 S3	2 P3	3 EP3	4 S3	5 P3	6 EP3	7 S3	8 P3	9 EP3
Subject ID									
Volume									
	10 S3	11 P3	12 EP3	13 S3	14 P3	15 EP3	16 S3	17 P3	18 EP3
Subject ID									
Volume									
	19 S3	20 P3	21 EP3	22 S3	23 P3	24 EP3	25 S3	26 P3	27 EP3
Subject ID									
Volume									
	28 S3	29 P3	30 EP3	31 S3	32 P3	33 EP3	34 S3	35 P3	36 EP3
Subject ID									
Volume									
	37 S3	38 P3	39 EP3	40 S3	41 P3	42 E P3	43 S3	44 P3	45 EP3
Subject ID									
Volume									
	46 S3	47 P3	48 EP3	49 S3	50 P3	51 EP3	52 S3	53 P3	54 EP3
Subject ID									
Volume									
	55 S3	56 P3	57 EP3	58 S3	59 P3	60 EP3	61 S3	62 P3	63 EP3
Subject ID									
Volume									
	64 S3	65 P3	66 EP3	67 S3	68 P3	69 EP3	70 S3	71 P3	72 EP3
Subject ID									
Volume									
	73 S3	74 P3	75 EP3	76 S3	77 P3	78 EP3	79 S3	80 P3	81 EP3
Subject ID									
Volume									

Signature:

Dispatch Sheet

(Format of transferring each sample)

**APCAPS**	**Name of Field-supervisor**		**Date of Transfer**		
	**Signature**				

				**Number of Vials**				**No. of Tubes for Blood and Saliva**						
**S. No.**	**Barcode**	**Name**	**Age / Sex**	**Box**	**Serum**	**Plas**	**E-P**	**G-2ml**	**P-3ml**	**P-6ml**	**Saliva**	**Fasting Glucose**	**HB**	**Remark**
1.														
2.														
3.														
4.														
5.														
6.														

Protocol for Collection of Saliva samples

Saliva collection should be done only at a place where chances of dust contamination are zero or least. After putting the label/bar-code on the collection tube (ensure that the label matches the one on the questionnaire and the blood sample tubes), follow these steps:

1. Ensure that the subject has not had anything to eat or drink 30 minutes before the sample collection.
2. In case of subjects who are smokers, ensure they have not smoked over last 30 minutes before sample collection.
3. Inform the subject that you are about to collect a saliva sample and explain the procedure involved.
4. First ask the subject to rinse the mouth thoroughly and then to spit out the first saliva that came in their mouth and wait for atleast 10 minutes.
5. Ask the subject to spit into the tube until the liquid saliva is between the two lines (2ml saliva till upper line). If they spit just over the upper line, then there is no need to throw the sample.
6. If the sample is too much, then carefully discard the extra saliva sample with the help of a pipette. Please discard the tip of the pipette once used – using the same tip again will contaminate the saliva sample.
7. Once 2ml of saliva sample has been collected, ask the subject to hold the container in the upright position and place the large cap over the funnel. Closing this cap will allow the plastic cover over the large cap to rupture and allow the DNA stabilizer to enter the tube and mix with the saliva sample. Place the tube in the test tube stand for 1 minute.
8. Thereafter, again hold the tube upright and unscrew the funnel from the tube.
9. Finally, tightly close the tube with the small cap and gently mix 6 times.
10. Place it in the ice-box in a test –tube stand.
11. Discard the funnel and large cap in the bio-waste polybag.

Saliva Tubes

After the completion of one village, the lab technician will arrange and pack all the saliva tubes to be transferred to SANCD, New Delhi with the help of field technician, field helper and other field workers under the supervision of field manager and project coordinator.

1. Separate all the saliva tubes into four lots based on the subject type.
2. After separating all the saliva tubes of DXA index children (subject ID starting with 1), their sibs (subject ID starting with 3), the mothers (subject ID starting with 5) and the fathers (subject ID starting with 6); arrange them in increasing order of their subject ID's.
3. After the four lots are arranged serially on the thermacol stands, all the saliva tubes of the members of one family will be tied together with the rubber band and will be then kept in a transparent plastic lock bags. Keep the list of the village handy to confirm the subject ID's of the members of each family.
4. While tying the saliva tubes note missing information, if any and other comments regarding the saliva tubes in the appropriate column of the dispatch sheet.
5. Repeat steps 3 and 4 for each family, such that different bundles representing families are made and kept in the plastic bags.

6. Once the plastic bag is full note down the village name, date of packing, packet number and the list of family ID of the tubes kept in that bag on a sheet of paper and place it inside the plastic bag such that information is visible from outside.
7. Lock the plastic bag and pack it with the cello tape. Take care not to over full the plastic bags to avoid its tearing.
8. Repeat Steps 3-6, till all the saliva tubes are packed.
   Make a record of total number of saliva tube packets of a village

Sample Transfer

All the samples will be transferred to SANCD, New Delhi from time to time (depending on the temporary storage capacity available in NIN, Hyderabad) by the lab and field technicians with the help of field helper and other field workers under the supervision of field manager and project coordinator. Please inform the SANCD staff well in advance to make arrangements for sample transfer.

Contact Person: Dr. Ruby Gupta – g_ruby2123@yahoo.co.in
Ms. Aastha Aggarwal (Mobile: 09873300590)
SANCD, New Delhi

Purple capped vaccutainers will be transferred to SANCD, New Delhi for DNA and RNA extraction. Two storage vials each for serum, fluoride plasma and EDTA plasma will also be transferred to SANCD, New Delhi for biochemical analysis. Grey capped vaccutainers and one storage vial each for serum, fluoride plasma and EDTA plasma will be stored at NIN, Hyderabad for back-up. This practice will be followed so that if something goes wrong with the samples stored in either NIN or SANCD, then there should be back up at the other centre.

Material to be transferred to SANCD, New Delhi	Material to be permanently stored at NIN, Hyderabad
Saliva Tubes	Questionnaires
Purple Capped 3 ml and 6 ml EDTA coated Vaccutainers	Grey Capped 2ml Sodium Fluoride coated vaccutainer
Two Storage Vials containing Serum	One Storage Vial containing Serum
Two Storage Vials containing Fluoride Plasma	One Storage Vial containing Fluoride Plasma
Two Storage Vials containing EDTA Plasma	One Storage Vial containing EDTA Plasma
Dispatch Sheet	NIN Record Sheet
SANCD Box Sheet	NIN Box Sheet
Village List	Village List
Copy of Daily Work Sheet	Daily Worksheet
Copy of QC Data	QC Data

While all the samples are being finally packed by the courier service providers, all the details regarding the sample transfer (number of boxes, number of packets, etc.) will be recorded by the field manager and mailed to SANCD lab in-charge. The soft copies of the NIN box sheets and NIN record sheets will also be mailed to SANCD lab in-charge for reference.

CLINIC QUESTIONNAIRE

Clinic questionnaire completed by fieldworker

- Filled in biro.
- It is only permissible to leave a section blank if it is in a shaded region.
- If a section is blank then cross through section.

Guide for conducting structured interviews

Structured interviews capture vital information. The aim of a structured interview is to measure facts and people's attitudes accurately and in such a way that if one were to repeat it at another time one would get the same answer.

It is, therefore, very important to get the structured interview right. Respondents need to be able to provide truthful answers which reflect their lives. Careful and precise interviewing techniques are essential to ensure the collection of complete, standardised, unbiased and accurate data. Much research has been undertaken to identify what works in getting the best from a structured interview and what does not.

For example:

- Interviewers who adopt a business like manner (wearing a dark suit) are less likely to gain trust- responders tend not to give truthful answers.
- Young interviewers obtain less reliable results than older interviewers.
- Male interviewers obtain less full information from male respondents than female interviewers and vice versa.

Response and accuracy is likely to be increased if the interviewer looks happy, appears positive. It is likely to be decreased if the interviewer looks tense. Matching up respondents with interviewers is particularly difficult. It is, therefore, vital that the questions in a structured interview are asked in the same way and that the responses are recorded in the same way by the interviewer.

The following provide essential steps and aids to help conduct the structured interview.

The interviewer

Foremost the interviewer needs to:

- Know and understand what the study is about- inside out.
- Understand the questionnaire
- Be familiar with the pre- coded questions and those which are open
- To be able to conduct the questionnaire in a uniform manner

Good interviewers

- Are sensitive and trustworthy
- Have an ability to establish a rapport with a wide range of people
- Are friendly and positive
- Are good listeners and do not interrupt people before they have finished speaking
- Are committed and persevering
- Have the ability to adopt a neutral manner (showing neither approval or disapproval)
- Have legible handwriting
- Are adept at leaving the respondent happy
- Are good planners
- Are able to ensure and maintain confidentiality
- Dress neutrally- suitable for any kind of home/clinic
- Have good intuition about when it is appropriate to approach respondents or not.

The interview

The interviewer must approach potential respondents in a positive manner in order to encourage them to participate. The critical moment is when the interviewer introduces themselves.

Always present your ID Card. Be honest about the study's aims and let them know how long the interview will take. Always provide respondents with details about the study and information on who to contact if/ should they want to.

Find a place where the interview can be conducted in private without interruptions.

Most respondents want to be seen in the best possible light and will want to give answers that reflect this. They will want to answer in such a way as to please the interviewer. The reliability of the information collected will be dependent on the rapport and satisfactory relationship established during the introduction. If a respondent feels anxious or uneasy they may be less likely to want to provide personal information.

When asking questions, the interviewer must always

- be sensitive to the needs of the respondent; e.g. not sitting in sunlight, comfortable seats etc.
- be encouraging
- use the exact words printed in the a questionnaire, and in the exact order:

  changing words or sentence orders will introduce serious bias to the study
- speak in a non- judgmental manner
- express polite interest
- read the questions and pre-coded answers out in reasonable volume and speed ensuring the respondent has heard and understood the question
- look at the respondent after each question to pick up on any visual clues of embarrassment or unease
- <u>accurately</u> record the answer on the questionnaire

NEVER

- appear surprised or disapproving
- express opinions or beliefs
- make assumptions about respondents likely answers
- hurry the respondent for answers
- ask questions in a biased or leading way
- ring/circle a category that comes nearest to the respondent's reply. If the code does not exist, record the exact words used by the respondent in their response
- allow long silences to become embarrassing
- apologise for asking personal or embarrassing questions

If the interviewer appears hesitant, reluctant, unconfident, negative or thinks a question is too personal, then the respondent will be influenced and will decline to give accurate answers. In fact they will encourage negative responses.

It is important to maintain the respondent's interest and motivation throughout the interview.

It is common for respondents to go off the topic and talk about other issues not relevant in the questionnaire. The interviewer has to be skilled to bring respondents back to the point. If this is done firmly from the onset, further problems are less likely to recur. The following phrases may be helpful:

"This is important information that we can cover a little later-can we now focus on"

"What you say is most interesting but could I just ask you now on"

Another problem with respondents who go off the topic is that they may have imparted some information that is useful in the questionnaire. Asking them again may give rise to irritability. BUT, it is very important never to assume the answer. To avoid potential irritability-prefix the question with:

"I know you have already mentioned this, but can I just check"

"Now you have already said something about this, but I would like to ask you this question".

It is never a good idea to break off the interview- it compromises the rapport and relationship already established. It is important to ensure that the respondent is fully aware of how long they are committing themselves to the interview.

If interviewers are seriously worried about a respondent (e.g. someone admitting suicidal thoughts or an elderly person being abused), the interviewer should offer to put the respondent in contact with a suitable professional. If accepted, ensure that this is put in writing and signed by the respondent. If the respondent refuses, only contact a professional if it is seen as a case of emergency.

Probing and Prompts

A probe is a stimulus which is used to obtain information form respondents who experience discomfort, hesitation, or feel unhappy to reveal information about themselves. Directive probing techniques are acceptable if one is eliciting factual information. Here it is important to motivate respondents in an undemanding and understanding tone. The aim is to encourage respondents to give accurate information. Probes listed below may help.

"Can you tell me more about"
"In what way"
"Can you describe"
"what sort of (office work do you do)"
"Before writing your answer down, can I just check?"

Third Parties

Caution would be exercised when other people want to sit in on the interview. The presence of a third party will always influence the respondent and will lead to biased answers. This is best avoided.

Hesitancy, misunderstanding and non-response

Some respondents may seem hesitant in answering questions and the interviewer could affirm this with "there is no right or wrong answer on this- we just trying to get your ideas. Some will ask the interviewer for their opinion and here it is important not to give opinions but to explain that it is the respondent's opinion that matters. Some may want to answer with a "don't know" and this can have many different meanings E.g. It may mask misunderstanding of the question, (see next paragraph), may not want to impart the information (use probes), or may genuinely not know. It is important to record such information on the questionnaire.

When respondents genuinely do not understand, they may ask the interviewer to explain it. In this case, the interviewer should repeat the question and if no answer is forthcoming make a note of it on the questionnaire. It is important not to succumb explaining the question. Interviewers will give different explanations evoking different answers.

If a respondent is very reluctant to answer a question, it may help if the interviewer

- Confirms the information given is confidential
- That replies will be aggregated in tables so that individual responses cannot be identified.

If the respondent still feels unwilling to respond, a note should be made of it and the next question pursued.

Ultimately, if respondents refuse to take part or answer any question, their wishes must be respected. The interviewer should apologise for the inconvenience. Most interviewers achieve 8/10 response rate.

Often a respondent will change their minds and a follow-up letter may help.

End of the interview

Interviewers must leave their respondents in a positive frame of mind. After the interview, interviewers must:

- Check the questionnaire to ensure all parts are completed
- Must be prepared to spend time explaining the study further
- Emphasise how the information obtained is vital to the study
- Ensure complete confidentiality
- Thank respondents for their willingness to share their experiences
- Ensure the respondent has information and a contact point about the study should they require further information

QUESTIONNAIRE

	**Interview details**		**COMMENTS**
**3.1**	Date of quest. Completion	___ ___ / ___ ___ / ___ ___ [DD/MM/YY]	
**3.2**	Time of quest. starting	☐☐:☐☐ [Hours:minutes; 24-hour clock]	Must be entered; use 24 hour clock
**3.3**	Interviewer code	☐☐	Each interviewer given codes starting from '1' to each of the team members
**3.4**	Interviewer initials	☐☐☐	These should decided at the start of the study and used consistently (e.g. don't flip between 2/3 alphabets
	First of all I would like to collect some details about you and where you live at present		
	**Contact details**		
**4.1**	Family name	_____ [Surname]	
**4.2**	First name/middle name	_____[Forename/other name]	Forename followed by middle name if any
**4.3**	Current house address (if any) [House No./Street/Locality]	_____ _____	This relates to person's current residence defined as place where the person spent most nights in the 12 months preceding the interview
**4.4**	Place name	_____ [Name of Village/Town/City]	
**4.5**	PIN Code	☐☐☐☐☐☐	6 digit postal information code; first digit can be 1-8
**4.6**	Sub-district	_____ [Tehsil/Taluk/Mandal//Municipality]	Names of sub-district vary according to state; these three cover the four states in the study, for other states prompt the subject by appropriate name (see list at the back)
**4.7**	District	_____	
**4.8**	Nearest railway station	_____	Railway station closest to the place and not the main junction.
**4.9**	Nearest big town	_____ [In case of village only]	
**4.10**	State	_____ [Name of country if abroad]	Total 35, see list at the back if unsure
**4.11**	Type of place	☐[1=Village; 2=Town; 3=Small city; 4=Large city]	Ask the subject; if unsure, negotiate on the basis of the definition provided; for large city, confirm from the list at the back if unsure (35 large cities); include urban outgrowths

**4.12**	Census code	☐☐☐☐☐☐☐☐☐☐	To be completed at base, leave blank
**4.13**	Home telephone number (landline)	(☐☐☐☐) ☐☐☐☐☐☐☐☐<br>[Area code] [Phone number]	Leave blank if no landline
**4.14**	Mobile number	☐☐☐☐☐☐☐☐☐☐	Leave blank if no mobile number

	Now I would like to collect some personal information about you		
	Personal details		
**5.1**	Age last birthday	☐☐ [In completed years]	
**5.2**	Day of birth	☐☐ [DD]	Put as 01 if unknown
**5.3**	Month of birth	☐☐ [MM]	Put as 07 if unknown
**5.4**	Year of birth	☐☐☐☐ [YYYY]	Check that year of birth corresponds to age at last birthday
**5.5**	Sex	☐ [1=Male; 2=Female]	
**5.6a**	**Sibling history**		
	(a) How many brothers (alive) do you have?	☐☐ [Enter 00 if None]	Only for alive brothers
	(b) How many sisters (alive) do you have?	☐☐ [Enter 00 if None]	Only for alive sisters
	(c) What was , your birth order in your family?	☐☐ [Enter 00 if None]	Only for alive siblings
	(d) Do you have a twin brother or sister?	☐ [1=Yes; 2=No]	
**5.6b**	**Ethnicity and religion**		
	(a)What is your place of origin?	_____	This would refer to the subjects ancestral origin – so state where the parents / grand parents came from – same/ different district, or same / different state.
	(b)Which category do you belong to?	☐ 1-General, 2-SC, 3-ST, 4-OBC, 5- Others	
	(c) What religion do you belong to?	☐ 1-Muslim, 2-Hindu, 3-Christian, 4-Other	
	(d) In case of Hindu General category, what caste do you belong to?	☐ 1- Brahmin,2- Kshatriya, 3-Vaish, 4- Other	
	(e) In case you are a Muslim, which category do you belong to?	☐ 1-Shia, 2- Sunni, 3- Other	
	(f) If belonging to a Tribe, which one do you belong to?	_____	Enter the name as appropriate
**5.6c**	**Consanguinity**<br>(a) If married, is your spouse a close relative before marriage? ☐ 1-Yes 2- No<br>(b) If yes, what is the relation? ☐ 1-Sibling , 2-First cousin(paternal/maternal), 3-Second cousin(paternal/maternal), 4-Uncle (maternal/paternal) 5- Niece (Paternal/maternal) 6.Other…….		

**5.7**	Current marital status		☐ 1=Never married<br>2=Married<br>3=Widow/widower<br>4=Separated/divorced	
**5.8**	*If ever married*:			
	(a) How old were you when you first started living with your spouse after your marriage?		☐☐ [Age in completed years]	
	(b) Does your spouse normally live with you now?		☐ [1=Yes; 2=No]	Do not ask for those with marital status 3 or 4 – leave blank
**5.9**	How many (live) sons do you have?		☐☐ [Enter 00 if None]	Only alive sons
**5.10**	How many (live) daughters do you have?		☐☐ [Enter 00 if None]	Only alive daughters
**5.10a**<br><br>**5.10b**	What was the index child/children's birth order (BO)?<br><br>Please recall whether the child received nutritional supplementation from the Anganwadi Yes=1 and No=2<br>i. If yes, at what age.		a. ☐☐☐☐<br><br>BO b. Supplemented i. Age<br>1.☐ ☐ _____<br>2.☐ ☐ _____<br>3 ☐ ☐ _____<br>4.☐ ☐ _____	a. Record birth order of index child/children<br><br>b. Record nutritional supplementation information for all children,<br>i. Record age in months
**5.10c**	What was the index child's gender?		☐☐☐☐ 1-male,2-female	
**5.10 d**	Was the child breast-fed?		☐☐☐☐ Yes =1 and No=2	Ask for each child
**5.10 e**	What was the duration of ***EXCLUSIVE*** breast-feeding?		☐☐☐☐ months	This refers to exclusive breast-feeding i.e. no other foods/'top-feeds'/complementary foods introduced.
	Primary occupation			
**5.11**	(a) Respondent: ☐		(b) Spouse (if married): ☐	Do not ask (b) if person unmarried
	1=At home doing housework	4= Student/ training	8=Skilled non-manual	If unsure, check with job descriptions in table.
	2=Unemployed, not seeking work: retired/ disabled	5=Unskilled manual 6=Semi-skilled manual	9=Semi-Professional	
	3=Unemployed, seeking work	7=Skilled manual	10=Professional	

**5.12**	Briefly describe your job:	_____	Enter short description of person's job
	Highest educational level attained		
**5.13**	(a) Respondent: ☐	(b) Spouse (if married): ☐	Do not ask (b) if person unmarried
	1=Illiterate	4=Secondary school (ITI course, class X/XII, Intermediate)	
	2=Literate, no formal education	5=Graduate (BA, BSc, BCom, Diploma)	
	3=Up to primary school (class IV)	6=Professional degree/postgraduate (MA, MSc, MBBS, MSW, BTech, PhD)	

	Now I am going to ask you some questions about your household		**COMMENTS**
	**Current household circumstances**		Questions below refer to a household. It is important to define the household before proceeding with the following questions. A household is a group of people (related or unrelated) who live together and take their meals together from a common kitchen unless the exigencies of work prevent them from doing so. The key element is kitchen.
**6.1**	What kind of household do you currently live in?	☐	
	1=Single<br>2=Hostel/shared accommodation<br>3=Nuclear family (married couple & offspring)<br>4=Extended family (2 related married couples of different generations i.e. married couple with one of the parents)	5=Joint family (two related married couples from same generation (i.e. two married siblings)<br>6=Joint-extended<br>7=Any other	A hostel here refers to any institutional household (group of unrelated persons taking their meal from a common kitchen). Persons living in a common building but not taking their meals from a common kitchen are classed as single/family type as appropriate. Common kitchen refers to joint cooking, not just sharing of a room used as kitchen).
**6.2**	What is the material used in the construction of the house?	☐ 1=Kutcha<br>2=Semi-pucca<br>3=Pucca	Kutcha (made from mud, thatch, or other low quality material);<br>Semi-pucca (partly low quality and high quality material);<br>Pucca (high quality material used throughout including roof, walls, floor)
**6.3a.**	What is the main source of lighting for your household?	☐ 1=Electricity<br>2=Kerosene<br>3=Gas<br>4=Oil<br>5=Other	For multiple sources, record the predominant one

**6.3b.**	What is the main source of cooking fuel?	☐ 1=Electricity<br>2=Kerosene<br>3=Gas<br>4=Oil<br>5=Other	For multiple sources, record the predominant one
**6.4**	What is the main source of drinking water for members of your household?	☐ 1=Pipe, hand pump, well (in residence/ plot)<br>2=Pipe, hand pump or well (public)<br>3=Other	For multiple sources, record the predominant one
**6.5**	What kind of toilet facility does the household have?	☐ 1=Own flush toilet<br>2=Own pit toilet/latrine<br>3=No facility/field/bush<br>4=Other	
**6.6**	Do you collect rations from a ration card?	☐ [1=Yes; 2=No]	
	**SKIP QUESTIONS 6.7-6.10 IF LIVING IN HOSTEL/SHARED ACCOMMODATION**		
**6.7**	Including yourself, how many people normally live in your household?	☐☐ [Number of People]	
**6.8a.**	How many rooms are there in your household? (count all rooms including kitchen, bathroom, etc)	☐☐ [Number of Rooms]	Include rooms normally available for use to the person. In case of person living in a hostel with access to a shared kitchen, common room and bathroom, this number would be three.
**6.8b.**	Do you have a separate kitchen at home?	☐ 1- Yes, 2 – No	
**6.8c.**	If yes, for how many years?		
**6.9**	Does this household own any agricultural land?	☐ [1=Yes; 2=No]	In case of institutional household (hostel), complete only for that individual
**6.10**	**Does the household own any of the following:**		Availability for use (e.g. provided by the employer) should be regarded as yes.
	(a) Clock/Watch	☐ [1=Yes; 2=No]	
	(b) Radio/Transistor/Tape recorder	☐ [1=Yes; 2=No]	
	(c) Television	☐ [1=Yes; 2=No]	
	(d) Bicycle	☐ [1=Yes; 2=No]	
	(e) Motorcycle/scooter/moped	☐ [1=Yes; 2=No]	
	(f) Car	☐ [1=Yes; 2=No]	
	(g) Refrigerator	☐ [1=Yes; 2=No]	
	(h) Telephone	☐ [1=Yes; 2=No]	
	(i) Water pump	☐ [1=Yes; 2=No]	
	(j) Bullock cart	☐ [1=Yes; 2=No]	
	(k) Thresher	☐ [1=Yes; 2=No]	
	(l) Tractor	☐ [1=Yes; 2=No]	

	Now thinking back to when you were a child, say 10-12 years old, please answer the following questions about the household where you lived at that time			**COMMENTS**
	**Household circumstances in childhood (at age 10-12 years)**			
**7.1**	What was your father's occupation at the time? ☐			
	1=At home doing housework	4= Student/ training	8=Skilled non-manual	If unsure, check with job descriptions in table.
	2=Unemployed, not seeking work: retired/ disabled	5=Unskilled manual<br>6=Semi-skilled manual	9=Semi-Professional<br>10=Professional	
	3=Unemployed, seeking work	7=Skilled manual	11=Died, left family	
**7.2**	What was the highest educational level attained by your mother? ☐			
	1=Illiterate	4=Secondary school (ITI course, class X/XII, Intermediate)		
	2=Literate, no formal education	5=Graduate (BA, BSc, BCom, Diploma)		
	3=Up to primary school (class IV)	6=Professional degree/postgraduate (MA, MSc, MBBS, MSW, BTech, PhD)		
**7.3**	What was the highest educational level attained by your father? ☐			
	1=Illiterate	4=Secondary school (ITI course, class X/XII, Intermediate)		
	2=Literate, no formal education	5=Graduate (BA, BSc, BCom, Diploma)		
	3=Up to primary school (class IV)	6=Professional degree/postgraduate (MA, MSc, MBBS, MSW, BTech, PhD)		
**7.4**	What was your mother's occupation at the time? ☐			
	1=At home doing housework	4= Student/ training	8=Skilled non-manual	If unsure, check with job descriptions in table.
	2=Unemployed, not seeking work: retired/ disabled	5=Unskilled manual<br>6=Semi-skilled manual	9=Semi-Professional<br>10=Professional	
	3=Unemployed, seeking work	7=Skilled manual	11=Died, left family	
**7.5**	What kind of household did you live in? ☐			
	1=Single<br>2=Hostel/shared accommodation<br>3=Nuclear family (married couple & offspring)<br>4=Extended family (2 related married couples of different generations i.e. married couple with one of the parents)	5=Joint family (two related married couples from same generation (i.e. two married siblings)<br>6=Joint-extended<br>7=Any other		A hostel here refers to any institutional household (group of unrelated persons taking their meal from a common kitchen). Persons living in a common building but not taking their meals from a common kitchen are classed as single/family type as appropriate. Common kitchen refers to joint cooking, not just sharing of a room used as kitchen).

**7.6**	What was the material used in the construction of the house?	☐ 1=Kutcha<br>2=Semi-pucca<br>3=Pucca	Kutcha (made from mud, thatch, or other low quality material);<br>Semi-pucca (partly low quality and high quality material);<br>Pucca (high quality material used throughout including roof, walls, floor)
**7.7a.**	What was the main source of lighting for your household?	☐ 1=Electricity<br>2=Kerosene<br>3=Gas<br>4=Oil<br>5=Other	For multiple sources, record the predominant one
**7.7b.**	What was the main source of cooking fuel?	☐ 1=Electricity<br>2=Kerosene<br>3=Gas<br>4=Oil<br>5=Other	For multiple sources, record the predominant one
**7.8**	What was the main source of drinking water for members of your household?	☐ 1=Pipe, hand pump, well (in residence/ plot)<br>2=Pipe, hand pump or well (public)<br>3=Other	For multiple sources, record the predominant one
**7.9**	What kind of toilet facility did the household have?	☐ 1=Own flush toilet<br>2=Own pit toilet/latrine<br>3=No facility/field/bush<br>4=Other	
**7.10**	Did you collect rations from a ration card?	☐ [1=Yes; 2=No]	
	**SKIP QUESTIONS 7.11- 7.14 IF LIVING IN HOSTEL/SHARED ACCOMMODATION**		
**7.11**	Including yourself, how many people normally lived in your household?	☐☐<br>[Number of People]	
7.12a	How many rooms were there in your household? (count all rooms including kitchen, bathroom, etc)	☐☐<br>[Number of Rooms]	Include rooms normally available for use to the person. In case of person living in a hostel with access to a shared kitchen, common room and bathroom, this number would be three.
7.12b.	Did you have a separate kitchen at home?	☐ 1- Yes, 2 – No	
7.12c	If yes, for how many years?		
7.13	Did this household own any agricultural land?	☐ [1=Yes; 2=No]	In case of institutional household (hostel), complete only for that individual
7.14	**Did the household own any of the following:**		Availability for use (e.g. provided by the employer) should be regarded as yes.
	(a) Clock/Watch	☐ [1=Yes; 2=No]	
	(b) Radio/Transistor/Tape recorder	☐ [1=Yes; 2=No]	

	(c) Television	☐ [1=Yes; 2=No]	
	(d) Bicycle	☐ [1=Yes; 2=No]	
	(e) Motorcycle/scooter/moped	☐ [1=Yes; 2=No]	
	(f) Car	☐ [1=Yes; 2=No]	
	(g) Refrigerator	☐ [1=Yes; 2=No]	
	(h) Telephone	☐ [1=Yes; 2=No]	
	(i) Water pump	☐ [1=Yes; 2=No]	
	(j) Bullock cart	☐ [1=Yes; 2=No]	
	(k) Thresher	☐ [1=Yes; 2=No]	
	(l) Tractor	☐ [1=Yes; 2=No]	

Now I will ask you a few questions about your health and lifestyle
Health and lifestyle

Comments

8.1	(i) Have your ever used tobacco on a REGULAR basis (at least weekly)?	(ii)Age at starting	(iii) Duration of use	(iv) Number of days per week	(v) Number of use or smoked per day	(vi) Time of day when first cigarette is smoked	Leave ii, iii, iv , v,vi blank if answer to i is never
(a) Smoked	1=Never ☐ 2=Former (stopped >6months) 3=Current (in last 6 months	☐☐ [Yrs]	☐☐ [Yrs]	☐ [Days]	☐☐	☐☐.☐☐ a.m/p.m	
(b) Chewed	1=Never ☐ 2=Former (stopped >6months) 3=Current (in last 6 months	☐☐ [Yrs]	☐☐ [Yrs]	☐ [Days]	☐☐	☐☐.☐☐ a.m/p.m	Ask specifically about paan masala, gutkha, khaini and zarda as the subject may not be aware that it contains tobacco.
(c) Snuffed	1=Never ☐ 2=Former (stopped >6months) 3=Current (in last 6 months	☐☐ [Yrs]	☐☐ [Yrs]	☐ [Days]	☐☐	☐☐.☐☐ a.m/p.m	

**8.2**	(a) Is there someone in your household who smokes tobacco at home? **[If no, skip to 8.3]**	☐ [1=Yes; 2=No]	
	If yes,<br>(b) How many cigarettes or beedis does this person smoke per day?	☐☐☐ beedis/ cigarettes per day	Do not include chewed or snuffed
**8.3**	(a) Has an indoor open fire with wood, crop residues or dung been used in your home as a primary means of cooking for more than 6 months in your life? **[If no, skip to 8.4]**	☐ [1=Yes; 2=No]	
	If yes,<br>(b) For how many years has wood, crop residues or dung been used for cooking in your home?	☐☐[Years]	Leave blank if answer to a is no
	(c) On average for how many hours a day have you personally spent cooking using wood, crop residues or dung?	☐☐[Hours] [00 if none]	
	(d) Is wood, crop residues or dung still used for cooking in your home?	☐ [1=Yes; 2=No]	
	(e) Was your stove or fire vented to the outside?	☐ [1=Yes; 2=No]	
**8.4**	Would you describe your present alcohol intake as?	☐ 1= Daily/most days<br>2= Weekends only<br>3= 1-2 times/month<br>4= Special occasions<br>5= Never	
**8.4a**	Measures or glasses per occasion<br>a. Locally made spirits<br>b. Branded spirits<br>c. Beer<br>d. Wine	No. of glasses<br>☐☐<br>☐☐<br>☐☐<br>☐☐	ml<br>☐☐☐<br>☐☐☐<br>☐☐☐<br>☐☐☐

	Now I will ask you a few questions about how you have been feeling in general. I will read out a list of statements, please tell me which one best describes your health state today.			**COMMENTS**
	**Quality of life**			
**9.1**	Mobility	☐	1= I have no problems in walking around;<br>2= I have some problems in walking around;<br>3=I am confined to bed	These questions refer to the subjects general health state. For each question read out each of the statements *<u>exactly as they are written.</u>*
**9.2**	Self care	☐	1= I have no problems with washing and dressing myself;<br>2= I have some problems with washing or dressing myself;<br>3=I am unable to wash and dress myself	
**9.3**	Usual activities	☐	(e.g. work, study, housework, family or leisure activities)<br>1= I have no problems with performing my usual activities;<br>2= I have some problems with performing my usual activities;<br>3=I am unable to perform my usual activities	

**9.4**	Pain/discomfort	☐	1= I have no pain or discomfort;<br>2= I have moderate pain or discomfort;<br>3=I have extreme pain or discomfort	
**9.5**	Anxiety/Depression	☐	1= I am not anxious or depressed;<br>2= I am moderately anxious or depressed;<br>3= I am extremely anxious or depressed	
**9.6**	We have drawn a scale on which the best state you can imagine is marked 100 and the worst state you can imagine is marked 0. Please indicate on this scale how good or bad your own health is today, in your opinion		☐☐☐%	Show them the scale. We want to know how they rate their own health

**Depression**			
	Over the last 2 weeks, how often have you been bothered by any of the following problems?	1=Not at all<br>2=Several days	3=More than half the days<br>4=Nearly every day
**9.7**	Little interest or pleasure in doing things		☐
**9.8**	Feeling down, depressed, or hopeless		☐
**9.9**	Trouble falling or staying asleep, or sleeping too much		☐
**9.10**	Feeling tired or having little energy		☐
**9.11**	Poor appetite or overeating		☐
**9.12**	Feeling bad about yourself, or that you are a failure, or have let yourself or your family down		☐
**9.13**	Trouble concentrating on things, such as reading the newspaper or watching television		☐
**9.14**	Moving or speaking so slowly that other people could have noticed. Or the opposite – being so fidgety or restless that you have been moving around a lot more than usual		☐
**9.15**	Thoughts that you would be better off dead, or of hurting yourself in some way		☐
**9.16**	In the last 4 weeks, have you had an anxiety attack – suddenly feeling fear or panic		☐ [1=Yes; 2=No]

IF NO, SKIP QUESTIONS 9.17-9.20 AND GO TO QUESTION 9.21

	If yes,	
**9.17**	Has this happened before?	☐ [1=Yes; 2=No]
**9.18**	Do some of these attacks come suddenly out of the blue – that is, in situations where you don't expect to be nervous or uncomfortable?	☐ [1=Yes; 2=No]
**9.19**	Do these attacks bother you a lot or are you worried about having another attack?	☐ [1=Yes; 2=No]

**9.20**	During your last bad anxiety attack, did you have symptoms like shortness of breath, sweating, your heart racing or pounding, dizziness or faintness, tingling or numbness, or nausea or upset stomach?	☐ [1=Yes; 2=No]
**9.21**	If you checked off any problems on this questionnaire so far, how difficult have these problems made it for you to do your work, take care of things at home, or get along with other people?	☐ 1=Not difficult at all<br>2=Somewhat difficult<br>3=Very difficult<br>4=Extremely difficult
*In the **last 4 weeks**, how much have you been bothered by any of the following problems?*		1=Not bothered<br>2=Bothered a little<br>3=Bothered a lot
**9.22**	Worrying about your health	☐
**9.23**	Your weight or how you look	☐
**9.24**	Difficulties with husband/wife, parents, or other relatives	☐
**9.25**	The stress of taking care of children, parents or other family members	☐
**9.26**	Stress at work outside of home or at school	☐
**9.27**	Financial problems or worries	☐
**9.28**	Having no one to turn to when you have a problem	☐
**9.29**	Something bad that happened **recently**	☐
**9.30**	Thinking or dreaming about something terrible that had happened to you **in the past** – like your house being destroyed, a severe accident, being hit or assaulted, or being forced to commit a sexual act	☐

	Now I will ask you questions relating to your usual sleep patterns.				**COMMENTS**
**10.1**	How many hours do you usually sleep per day (including sleep at night and during the day) on a typical day when you have school or work the next day?	☐☐.☐ [Completed half hours]			Total hours slept at night with during day
**10.2**	How many hours do you usually sleep per day (including sleep at night and during the day) on a typical day when you **do not** have school or work the next day?	☐☐.☐ [Completed half hours]			Total hours slept at night with during day
**10.3**	(a) Do you undertake shift work that interrupts your usual sleep patterns?	☐ [1=Yes; 2=No]			
	(b) *If yes,* how often is the shift work (over the last month)?	☐ 1=Daily<br>2=5-6 times/week	3=2-4 times/week<br>4=Once a week	5=2-3 times/month<br>6=Once a month	Leave blank if answer to a is no. Ask to recall over last month
**10.4**	In the past month, how often have you experienced difficulties in getting to sleep?	☐ 1=Daily<br>2=5-6 times/week	3=2-4 times/week<br>4=Once a week	5=2-3 times/month<br>6=Once a month<br>7=Never	Ask to recall over last month
**10.5**	In the past month, how often have you been bothered by awakening during night?	☐ 1=Daily<br>2=5-6 times/week	3=2-4 times/week<br>4=Once a week	5=2-3 times/month<br>6=Once a month<br>7=Never	Ask to recall over last month

Physical activity questionnaire

INSTRUCTIONS FOR COMPLETION OF PAQ

The physical activity questionnaire aims to assess the habitual physical activity of the individual, by ascertaining recall over the one week.

PHYSICAL ACTIVITY QUESTIONNAIRE

Ask the subject, how many hours he/she spends working (Q1). **This will be easy to ascertain if the person has regular working hours, as is the case with a factory worker on the shop floor. On the other hand executives may tend to work irregular hours and you may have to resort to asking them *"On average, how many hours do you work?"***

Once you have determined how many hours they spend at work, ask them how many hours they spend in 'standing', 'sitting', and 'walking'. Please use prompt by listing the sample activities provided under these headings in the questionnaire. The distribution of time spent, will depend on the occupation of the individual. Executives for instance may spend a substantial amount of their time sitting. Manual workers in a factory may spend substantial times 'walking' or on activities more strenuous than walking.

Ask whether the participant participated in each activity listed under the category "more strenuous than walking". If they participated in the activity, record frequency and duration.

<table>
<tr><td></td><td colspan="4">Now I am going to ask you questions about the time you spent doing different types of physical activity. Please recall the activities that you did in the LAST TYPICAL WEEK.

In case you travelled for a long duration to reach this place, or stayed back in this place for a few days, please recall the activities of the week before you left to this place.

The first questions are about your work/college. This includes paid jobs, working in your farm, study/training, any volunteer work or college activities.

Do not include unpaid work you might do around your home, like housework, garden work, and caring for your family. I will ask you about these later.</td></tr>
<tr><td></td><td colspan="2">Work related activity</td><td colspan="2"></td></tr>
<tr><td>11.1</td><td colspan="2">Do you currently have a job or do any unpaid work or study/training? Do not include household work, we will ask about this later.</td><td colspan="2">☐ [1=Yes; 2=No]
[IF NO, SKIP TO 11.8]</td></tr>
<tr><td>11.2</td><td colspan="2">How many days did you work at the job or unpaid work in the last week?</td><td colspan="2">☐ [In completed days]</td></tr>
<tr><td>11.3</td><td colspan="2">In the last week, how many hours per day did you spend at this work?</td><td colspan="2">☐☐.☐
[In completed half hours]</td></tr>
<tr><td></td><td colspan="4">Of the hours you spend at work in a day during the last week I am going to ask you how many hours you spend in standing, sitting, walking and other strenuous activities (completed half hours):</td></tr>
<tr><td></td><td>(a) Standing: E.g. talk, lab work, supervise, mild cleaning, cattle grazing done standing.</td><td>(b) Sitting: E.g. typing, computer work, cleaning grains, eating lunch, driving for your work, etc</td><td colspan="2">(c) Walking: E.g. walking around, strolling, walking with light loads</td></tr>
<tr><td></td><td>☐☐.☐ [hours]</td><td>☐☐.☐ [hours]</td><td colspan="2">☐☐.☐ [hours]</td></tr>
<tr><td>11.4</td><td colspan="4">If you spend any time at work on activities more strenuous than walking, please list these:</td></tr>
<tr><td></td><td></td><td>(i) Took part in this activity</td><td>(ii) Days per week</td><td>(iii) Total duration per day</td></tr>
</table>

	(a) Carrying/walking with loads (15-25 kg)	☐ [1=Yes; 2=No]	☐ days	☐☐☐ [mts]
	(b) Carrying/walking with heavy load (>25 kg)	☐ [1=Yes; 2=No]	☐ days	☐☐☐ [mts]
	(c) Lifting / loading of weights	☐ [1=Yes; 2=No]	☐ days	☐☐☐ [mts]
	(d) Pushing cart with a load	☐ [1=Yes; 2=No]	☐ days	☐☐☐ [mts]
	(e) Ploughing	☐ [1=Yes; 2=No]	☐ days	☐☐☐ [mts]
	(f)Digging	☐ [1=Yes; 2=No]	☐ days	☐☐☐ [mts]
	(g) Watering / weeding fields	☐ [1=Yes; 2=No]	☐ days	☐☐☐ [mts]
	(h) Cut / chop wood or stones	☐ [1=Yes; 2=No]	☐ days	☐☐☐ [mts]
	(i) Harvesting	☐ [1=Yes; 2=No]	☐ days	☐☐☐ [mts]
	(j) Any others?	☐ [1=Yes; 2=No]		
	(k)_____		☐ days	☐☐☐ [mts]
	(l)_____		☐ days	☐☐☐ [mts]
	(m)_____		☐ days	☐☐☐ [mts]
	**Travel to and from work**<br>*Now think about how you travelled to and from work over the LAST WEEK. Please do not include travelling activities if you have already mentioned while we discussed your work/college activities.*			
			(a) Days per week	(b) Total duration per day
**11.5**	During the last week, how many days did you travel on a motorised vehicle, like a car, bus, auto-rickshaw or motorcycle to and from work?		☐ days	☐☐☐ [mts]
**11.6**	During the last week, on how many days did you cycle to and from work?		☐ days	☐☐☐ [mts]
**11.7**	During the last week, on how many days did you walk to and from work?		☐ days	☐☐☐ [mts]
	**Travel apart from to and from work**<br>*Now think about how you travelled from place to place over the LAST WEEK, including places like stores, movies, visiting relatives etc but excluding to and from work. Please do not include travelling activities if you have already mentioned.*			
			(a) Days per week	(b) Total duration per day
**11.8**	During the last week, how many days did you travel to places on a motorised vehicle, like a car, bus, auto-rickshaw or motorcycle except to and from work?		☐ days	☐☐☐ [mts]
**11.9**	During the last week, on how many days did you travel to places on a bicycle except to and from work?		☐ days	☐☐☐ [mts]
**11.10**	During the last week, on how many days did you did you travel to places by walking except to and from work ?		☐ days	☐☐☐ [mts]
	Now I am going to ask you some questions about how you spent your time, apart from work outside of the home over the LAST WEEK			
**11.11**	**Sports / games / exercise**<br>*Now think about all the physical activities that you did in the last 7 days solely for sport, exercise of leisure. Please do not include any activities you have already mentioned.*			

	Name of activity	(i) Took part in this activity	(ii) Days per week	(iii) Total duration per day
	(a) Walking normal speed for leisure	☐ [1=Yes; 2=No]	☐ days	☐☐☐ [mts]
	(b) Walking brisk speed for leisure	☐ [1=Yes; 2=No]	☐ days	☐☐☐ [mts]
	(c) Jogging/Running	☐ [1=Yes; 2=No]	☐ days	☐☐☐ [mts]
	(d) Badminton	☐ [1=Yes; 2=No]	☐ days	☐☐☐ [mts]
	(e) Cricket	☐ [1=Yes; 2=No]	☐ days	☐☐☐ [mts]
	(f) Yoga	☐ [1=Yes; 2=No]	☐ days	☐☐☐ [mts]
	(g) Swimming	☐ [1=Yes; 2=No]	☐ days	☐☐☐ [mts]
	(i) Volleyball	☐ [1=Yes; 2=No]	☐ days	☐☐☐ [mts]
	(j)			
	(k)			
	(m) Any others?	☐ [1=Yes; 2=No]		
	(n)_____		☐ days	☐☐☐ [mts]
	(o)_____		☐ days	☐☐☐ [mts]
	(p)_____		☐ days	☐☐☐ [mts]

**11.12**	**Household activities**<br>*Now think about activities you do at home such as housework, gardening and hobbies. Please do not include any activities already mentioned.*			
	Name of activity	(i) Took part in this activity	(ii) Days per week	(iii) Total duration per day
	(a) Cooking	☐ [1=Yes; 2=No]	☐ days	☐☐☐ [mts]
	(b) Washing vessels	☐ [1=Yes; 2=No]	☐ days	☐☐☐ [mts]
	(c) Mopping	☐ [1=Yes; 2=No]	☐ days	☐☐☐ [mts]
	(d) Sweeping	☐ [1=Yes; 2=No]	☐ days	☐☐☐ [mts]
	(e) Wash clothes manually	☐ [1=Yes; 2=No]	☐ days	☐☐☐ [mts]
	(f) Dusting / cleaning	☐ [1=Yes; 2=No]	☐ days	☐☐☐ [mts]
	(g) Ironing and folding clothes	☐ [1=Yes; 2=No]	☐ days	☐☐☐ [mts]
	(h) Child care	☐ [1=Yes; 2=No]	☐ days	☐☐☐ [mts]
	(i) Collecting fuel/fodder/water	☐ [1=Yes; 2=No]	☐ days	☐☐☐ [mts]
	(j) Animal care	☐ [1=Yes; 2=No]	☐ days	☐☐☐ [mts]
	(k) Gardening	☐ [1=Yes; 2=No]	☐ days	☐☐☐ [mts]
	(l) Any others?	☐ [1=Yes; 2=No		
	(m) Washing clothes by machine	☐ [1=Yes; 2=No	☐ days	☐☐☐ [mts]
	(n)_____		☐ days	☐☐☐ [mts]
	(o)_____		☐ days	☐☐☐ [mts]

11.13	**Sedentary activities**<br>*The last question is about time spent sitting in the last 7 days. Do not include time spent sitting at work Please do not include any activities already mentioned.*			
	Name of activity	(i) Took part in this activity	(ii) Days per week	(iii) Total duration per day
	(a) Reading for leisure	☐ [1=Yes; 2=No]	☐ days	☐☐☐ [mts]
	(b) Computer/computer games/internet for leisure	☐ [1=Yes; 2=No]	☐ days	☐☐☐ [mts]
	(c) Watching TV/ movies	☐ [1=Yes; 2=No]	☐ days	☐☐☐ [mts]
	(d) Indoor games (e.g. chess, carom, playing cards)	☐ [1=Yes; 2=No]	☐ days	☐☐☐ [mts]
	(e) Prayer/meditation	☐ [1=Yes; 2=No]	☐ days	☐☐☐ [mts]
	(f) Listening to music/radio	☐ [1=Yes; 2=No]	☐ days	☐☐☐ [mts]
	(g) Sewing/embroidery/knitting	☐ [1=Yes; 2=No]	☐ days	☐☐☐ [mts]
	(h) Socialising (talking outside working hours or on phone)	☐ [1=Yes; 2=No]	☐ days	☐☐☐ [mts]
	(i) Any others?	☐ [1=Yes; 2=No]		
	(j) Sitting idle	☐ [1=Yes; 2=No]	☐ days	☐☐☐ [mts]
	(k)_____		☐ days	☐☐☐ [mts]
	(l)_____		☐ days	☐☐☐ [mts]

FOOD FREQUENCY QUESTIONNAIRE

INSTRUCTION TO SUBJECT: *I am now going to ask you about the food that you have eaten over the last year. If you have not heard of an item please answer **"No".***

	CEREALS	**Portion Size**	**(a) Average consumption**	**(b) Per Day[1]**	**(b) Per Week[2]**	**(b) Per Month[3]**	**(b) Per Year/ Never[4]**
**12.1**	Chapathis / roti	No					
**12.2**	Parathas / naan	No					
**12.3**	Jowar roti	No					
**12.4**	Poori, bhatura	No					
**12.5**	Plain rice	Bowl					
**12.6**	Mutton, chicken biriyani	Bowl					
**12.7**	Lime rice, puliogare, veg biriyani	Bowl					
**12.8**	Bhagar	Bowl					

**12.9**	Upma	Bowl					
**12.10**	Idlis	No					
**12.11**	Dosa / uthappam	No					
**12.12**	Pesarattu	No					
**12.13**	Attakalu	Bowl					
**12.14**	Rice, ragi porridge	Bowl					
**12.15**	Corn flakes	Bowl					
**12.16**	Bread, Toast, Rolls, Buns	No					
**12.17**	Noodles, pasta etc	Bowl					
	**LENTILS / DHALS / GRAVIES**						
**13.1**	Plain dhal sambar	Ladle					
**13.2**	Dhal sambar with vegetables	Ladle					
**13.3**	Channa, rajma, dry peas etc. curry	Ladle					
**13.4**	Green leafy vegetable curry	Ladle					
**13.5**	Rasam, all types	Ladle					
		**Portion Size**	(a) Average consumption	**(b) Per Day[1]**	**(b) Per Week[2]**	**(b) Per Month[3]**	**(b) Per Year/ Never[4]**
	**CHUTNEYS / SALAD / PAPAD**						
**14.1**	Raw vegetable salad	Tbsp					
**14.2**	Vegetable Raitha	Tbsp					
**14.3**	Pickle	Tsp					
**14.4**	Papad	No					
**14.5**	Coconut chutney	Tbsp					
**14.6**	Groundnut chutney	Tbsp					
**14.7**	Tomato chutney	Tbsp					
	**NON – VEGETARIAN**						
**15.1**	Chicken curry	Bowl					
**15.2**	Chicken fry/grilled	No					
**15.3**	Mutton/ pork/beef curry or fry	Bowl					

**15.4**	Fish curry	Bowl					
**15.5**	Fish fry	No					
**15.6**	Organ meats (Liver, brain, kidney etc.)	Tbsp					
**15.7**	Prawn, crab, shell fish etc.	Bowl					
**15.8**	Egg (boiled, poached, omelettes)	No					
	**MILK & BEVERAGES**						
**16.1a.**	Tea –with milk	Glass					
**16.1b.**	Tea –without milk	Glass					
**16.2 a.**	Coffee – with milk	Glass					
**16.2 b.**	Coffee-without milk	Glass					
**16.3**	Plain milk	Glass					
**16.4**	Flavored milk (horlicks, bournvita etc)	Glass					
**16.5**	Curds	Bowl					
**16.6**	Buttermilk/Lassi	Glass					
		**Portion Size**	(a) Average consumption	**(b) Per Day[1]**	**(b) Per Week[2]**	**(b) Per Month[3]**	**(b) Per Year/ Never[4]**
**16.7**	Lime/ orange/ other fresh fruit juice	Glass					
**16.8**	Fanta, pepsi, coca cola etc.	250ml bottle					
**16.9**	Beer	Bottle					
**16.10**	Spirits (whiskey, gin, rum, arrack)	30ml peg					
**16.11**	Other local alcoholic drinks	30ml peg					
	**MISCELLANEOUS**						
**17.1**	Ghee/ butter	Tsp					
**17.2**	Jam	Tsp					
**17.3**	Sugar	Tsp					
**17.4**	Salt	Tsp					
	**SNACKS/ SWEETS/DESSERTS**						

**18.1**	Mixture, namkeen, chiwda, khara boondi, dalmoth	Tbsp					
**18.2**	Vada, all types	No					
**18.3**	Nuts (groundnuts, cashewnuts etc.)	Tbsp					
**18.4**	Chips/salted packed snacks (bingo, kurkure etc)	Bowl					
**18.5**	Samosa,bajji ,bonda, cutlet, patties	No					
**18.6**	Salted biscuits (krackjack, bakery biscuits)	No					
**18.7**	Sweet biscuits (Marie/good day/cream biscuits)	No					
**18.8**	Murukku , chakli, sakinalu	No					
**18.9**	Cakes or sweet pastries	No					
**18.10**	Payasam, kheer	Bowl					
		**Portion Size**	(a) Average consumption	**(b) Per** Day[1]	**(b) Per Week[2]**	**(b) Per Month[3]**	**(b) Per Year/ Never[4]**
**18.11**	Ice cream	Bowl					
**18.12**	Jamoon, Jilebi, Jangir etc.	No					
**18.13**	Mysore pak, ladoo, barfis	No					
**18.14**	Baksham	No					
**18.15**	Dried fruits (dates, figs, raisins etc)	Tbsp					
**18.16**	Chocolates	Small Bar					

	**FRUITS**	*Portion size*	**(a) Average consumption**	**(b) Per Day[1]**	**(b) Per Week[2]**	**(b) Per Month[3]**	**(b) Per Year/ Never[4]**	(c) Seasonal **(cross if seasonal)**
**19.1**	Banana	No						
**19.2**	Apple	No						
**19.3**	Orange	No						
**19.4**	Mango	No						
**19.5**	Guava (amrood)	No						
**19.6**	Grapes (angoor)	Bowl						
**19.7**	Pineapple	Slice						
**19.8**	Papaya (papita)	Slice						
**19.9**	Pomegranate (anar)	No						
**19.10**	Sapota (Chikoo)	No						
**19.11**	Watermelon(tarbooj)	Bowl						
**19.12**	Musk melon (kharbooj)	Bowl						
**19.13**	Custard apple	No						
**19.14**	Zizyphus (ber)	No						
**19.15**	Sugarcane (ganaa)	Pieces						
**19.16**	Palmyra	No						
	**VEGETABLES**							
		**Portion size**	(a) Average consumption	**(b) Per** Day[1]	**(b) Per Week[2]**	**(b) Per Month[3]**	**(b) Per Year/ Never[4]**	(c) Seasonal **(cross if seasonal)**
**20.1**	Palak, methi, other leafy vegetables	Tbsp						
**20.2**	Potato, sweet potato	Tbsp						
**20.3**	Beetroot/ radish	Tbsp						
**20.4**	Cabbage	Tbsp						
**20.5**	Beans, cluster beans	Tbsp						
**20.6**	Ladies finger	Tbsp						
**20.7**	Cauliflower	Tbsp						
**20.8**	Bottlegourd (lauki), ashgourd, Ridgegourd (turai), snakegourds, etc.	Tbsp						
**20.9**	Brinjal	Tbsp						
**20.10**	Kovai	Tbsp						

**20.11**	Capsicum/ green pepper	Tbsp						
**20.12**	Drumstick	Pieces						
**20.13**	Raw plantain	Tbsp						
**20.14**	Colacasia (arvi)	Tbsp						

21.1	Which type of oil is consumed most by your family? State in order of decreasing quantity of use.	☐<br>☐<br>☐<br>☐<br>☐<br>☐	Quantity in Kg/month<br>1=Sunflower oil ☐☐.☐☐<br>2=Groundnut oil ☐☐.☐☐<br>3=Coconut oil ☐☐.☐☐<br>4=Palm oil ☐☐.☐☐<br>5=Mustard oil ☐☐.☐☐<br>6=Dalda /vanaspathi ☐☐.☐☐	7=Butter ☐☐.☐☐<br>8=Ghee ☐☐.☐☐<br>9=Olive oil ☐☐.☐☐<br>10=Corn ☐☐.☐☐<br>11=Rice bran oil ☐☐.☐☐<br>12=Soya bean oil ☐☐.☐☐<br>13=Cotton seed oil ☐☐.☐☐
**21.2**	How many coconuts do you use for cooking in a month?	☐☐ [No / month] [00 if none]		
**21.3**	(a) What type of milk do you regularly consume?	☐	1=Whole milk<br>2=Skimmed milk<br>3=Toned milk	4=Skimmed milk powder<br>5=Whole and toned milk<br>6=Other
	(b) If other, then specify_____			
**21.4**	(a) Do you consume any vitamin or mineral supplement ☐ [1=Yes; 2=No] at least once a week?			

	If Yes, (b) **Brand name / Type**	(c) **Dosage(mg)**	(d) **No. / week**

**21.5**	Are you vegetarian?	☐ [1=Yes; 2=No]
**21.6**	Are you on any of the following special diets?	
	(a) Diabetic diet	☐ [1=Yes; 2=No]
	(b) Low fat diet	☐ [1=Yes; 2=No]
	(c) High fiber diet	☐ [1=Yes; 2=No]
	(d) Low salt diet	☐ [1=Yes; 2=No]
	(e) Weight reducing diet	☐ [1=Yes; 2=No]
	(f) Other	☐ [1=Yes; 2=No]
	(g) If other, please specify	1.<br><br>2.
	If yes,<br>(h) Since how many years are you on this special diet?	1. ☐☐ [completed years]<br>2. ☐☐ [completed years]

INSTRUCTIONS FOR COMPLETION OF FFQ

The aim of the questionnaire is to obtain the average food intake of an individual over a one year period. The questionnaire, after entry into a computer, will provide details of consumption of various nutrients – energy, carbohydrates, fat, proteins, vitamins and minerals. Entry of each food item correctly is essential as different foods provide different nutrients. Please use the portion size provided as they guide the subject in reporting the quantity he / she consumes.

COMPONENTS OF THE QUESTIONNAIRE:

The questionnaire comprises two components:

1. A food list of 98 food items
2. A set of questions about diet.

FOOD LIST

The food items are grouped under the following headings to help the subject recall the foods better:

- Cereals
- Lentils/Dhals/Gravies
- Chutneys / salad/ papad
- Non-vegetarian
- Milk and Beverages
- Miscellaneous
- Snacks / sweets/ desserts
- Fruits
- Vegetables

DIET RELATED QUESTIONS:

This comprises a set of questions aimed at eliciting further information on the food pattern of an individual. Information on the type of oil used for cooking of food, coconuts used for cooking, type of milk used, usage of vitamins and mineral tablets/capsules and any special diet adhered to are recorded in this section.

ADMINISTERING THE QUESTIONNAIRE:

Please familiarise yourself with the food frequency questionnaire before administering it.

INTERVIEWING THE SUBJECT:

Some of the food items may not be familiar to the subject. If the subject is not aware of the food item, please proceed to the next item in the list.

Please place the standard serving sizes e.g. katori etc. in front of the subject before you start the food frequency questionnaire. This will help the subject to estimate the food quantities that he/she is reporting.

PORTION SIZES:

Standard serving measure with volumes

Bowl = 220ml

Glass = 125 ml

Teaspoon = 5 ml

Tablespoon - 10 ml

Ladle = 56 ml

Inform the subject before beginning the questionnaire:

"We are doing this study at a national level and there may be several food items in the list that you may not have heard of but are eaten in other places .If we name an item you have not heard please answer'no'so that we proceed to the next question.

Then proceed to ask the questions and make entries in the method specified below to obtain and document information in the **food frequency** questionnaire:

1. *Ask the subject if they consume each of the food items listed in the questionnaire. For instance,* ***"Do you consume chapathis?"*** *If yes,*
2. *Ask the subject* ***"During the past one year, how often have you consumed this? Daily, weekly or monthly?"*** *…… If daily,*
3. *Ask* ***"On average how many times would you consume chapathis each day?"*** *If the answer is two times (twice), enter '2' under the "Per Day" column.*

   This provides the 'frequency of consumption''of the food item.
4. *Then ask, "****How many chapathis on average do you eat each time?" ….*** *If the answer is '3', enter '3' under the "Average consumption" column. This example is illustrated below:*

This is the 'average intake' of the food item.

**CEREALS**	**Portion size**	**(a) Average consumption**	**(b) Per Day**	**(c) Per Week**	**(d) Per Month**	**(e) Per Year / Never**
Chapathis/parathas/naan	No	3	2			

5. **If there are any foods that a subject does not consume, enter, '0' under the "average consumption" column and '0' in the "Per Year/ Never" column (two entries).**
For example if a person never eats Pizza's or Burgers, the entry would be as shown below:

**CEREALS**	**Portion size**	**(a) Average consumption**	**(b) Per Day**	**(c) Per Week**	**(d) Per Month**	**(e) Per Year / Never**
Pizza, Burger	No	0				0

PLEASE NOTE, THEREFORE, THAT *EVERY* LINE MUST HAVE AN ENTRY

6. When the subjects reports an average portion size as:
   2 to 3 chapathis
   Record as 2 ½ chapathis and NOT 2 - 3 or 2.5
   If for example an individual reports that they have 2-3 chapathis twice a day, your entry will be:

**CEREALS**	**Portion size**	**(a) Average consumption**	**(b) Per Day**	**(c) Per Week**	**(d) Per Month**	**(e) Per Year / Never**
Chapathis	No	2½	2			

7. For Miscellaneous foods, ask the subject if they add these foods on the table.
   For instance,
   - Butter, jam, cheese added to bread or toast would come under this category as would tomato sauce added to food on the table
   - Ghee added to rice or other food at the table would come under this category. However, ghee added during preparation/cooking as for instance during the preparation of sweets would not come under this category.
   - For sugar, the amount of additional sugar added to a glass of tea/ coffee or milk at the table is considered. If the subject reports that no sugar is added enter '0'.
     a. *For this reason ask "Do you add additional sugar to tea and coffee. If the answer is YES,*
     b. *Ask "How much for each glass?" If the answer is "2 teaspoons"*
     c. *enter '2' under the "Average consumption" column,*
     d. *if a person has 1 glass of tea twice a day, and 1 glass of coffee twice a day, enter '4' for the total number of times sugar is added to beverages per day under the "Per day" column*. Enter this information as indicated below:

**MISCELLANEOUS**	**Portion size**	**(a) Average consumption**	**(b) Per Day**	**(c) Per Week**	**(d) Per Month**	**(e) Per Year / Never**
Sugar	Tsp	2	4			

8. For foods where multiple items are listed, ask the individual how often they consume all these foods
   *For example, "*Lime rice, puliogare, curd rice, veg biriyani*".*
   If the subject reports that he eats one bowl of lime rice once a week and one bowl of veg biriyani once a week record as '2' under the 'Per Week' column (i.e. both Lime rice and veg biriyani together)

**CEREALS**	**Portion Size**	**(a) Average consumption**	**(b) Per Day**	**(c) Per Week**	**(d) Per Month**	**(e) Per Year / Never**
Lime rice, puliogare, veg biriyani.	Bowl	1		2		

9. Use general items where ever specific foods are not mentioned. For instance, coconut biscuits, cream biscuits will be entered under "sweet biscuits". Similarly, groundnuts, cashew nuts and other type of nuts will be entered under "nuts".
10. If individuals consume a combination of preparations eg. paneer mutter masala OR mixed groundnut and coconut chutney, enter the food under that item which is in greater proportion.
11. For fruits and vegetables ask the subject if they consume that food only in season or year round. If seasonal put a cross for the item in the appropriate column.

    For example if a person eats one banana a day throughout the year and 1 apple a day when apples are in season, your entry will be:

**FRUIT**	**Portion size**	**(a)Average consumption**	**(b)Per Day**	(c)**Per Week**	**(d)Per Month**	**(e)Per Year / Never**	**(f) Seasonal (cross if seasonal)**
Banana	No	1	1				
Apple	No	1	1				X

AVERAGE PORTION SIZES OF FOODS NOT LISTED IN THE QUESTIONNAIRE:

If individuals report consumption of foods below regularly, use the measure provided below.

1 big (hotel) idli = 1 ½ servings of medium size idli

1 set dosa = 1 ¼ servings of dosa

1 bhatura = 1 ½ servings of medium size poori

1 small ragi ball = ½ serving of a medium size ball

1 bun = 4 servings of bread slices

50 gms mixture/ namkeen = 3 servings of 1 tbsp mixture

1 bakery sweet / salt biscuit OR Cream Biscuit OR Cookie = 1 ½ serving of sweet/salt biscuits

1 small banana = ½ serving of medium banana

1 cup vegetables = 8 servings of vegetables in tablespoons

1 peg = 30 ml (made up to 1 glass with water or soda)

1 large peg = 45 ml (made up to 1 glass with water or soda) = 1 ½ 30 ml peg

FOOD LIST DESCRIPTIONS:

All the food items listed in the food list in the FFQ have been described in the following section, so that the interviewers understand the foods items listed. All food items have been listed and described in the same order listed in the questionnaire.

CEREALS:

12.1 Chapathis/roti: Refers to those Indian breads made of wheat flour prepared on a tawa without oil.
12.2 Parathas/naan: Refers to all types of Indian breads and parathas made of processed flour with added oil, prepared on a tawa.
12.3 Jowar roti: Indian rotis made with jowar flour
12.4 Poori, bhatura: Refers to Indian breads fried in oil.
12.5 Plain rice: Cooked raw/parboiled rice.
12.6 Mutton, chicken pulao/ biriyani: Includes all non-vegetarian fried rice.
12.7 Lime rice, puliogare, veg biriyani : refers to rice based preparations that are seasoned and/or flavored in oil. Included are foods such as vangi bath, tamarind rice, coconut rice, fried rice with vegetables (veg biriyani) etc.
12.8 Bhagar : Recipe containing rice cooked with potato and tomato.
12.9 Upma: Prepared with rava (sooji)/vermicelli/rice cooked with seasonings with or without vegetables.
12.10 Idlis: Steamed preparation made with batter of rice/ vermicelli/rava and black gram (urad) dhal.
12.11 Dosa, uthappam: Indian pancake made with the batter of rice/rava/wheat flour/rice flour with or without dhal (mainly black gram dhal). Includes rava dosa, set dosa, dosa with filling etc.
12.12 Pesarattu: Indian pancakes made on tawa with a batter of green gram (moong) dhal and spices.
12.13 Attakalu: Prepared with beaten rice seasoned with a little oil.
12.14 Rice, ragi porridge: Porridge (kanji) made with rice or ragi, without milk
12.15 Corn flakes: Includes cornflakes consumed with milk.
12.16 Bread, Toast, Rolls, Buns: Refers to all types of breads, buns, rolls etc. plain, toasted, sandwiched, or grilled.
12.17 Noodles, pasta etc: Includes the ready-to-use (e.g. Top Ramen, Maggi, Pasta Treat etc) and prepared vegetarian and non-vegetarian noodles and macaroni and other pastas.

LENTILS / DHALS / GRAVIES

13.1 Plain dhal sambar: Refers to sambar or dhal prepared with red gram dhal (tur dhal) **without** any vegetables.
13.2 Dhal with vegetables: Refers to sambar or dhal prepared with red gram dhal (tur dhal) **with** vegetables.
13.3 Channa, rajma, dry peas etc. curry: Refers to curry prepared with any whole gram/pulses.
13.4 Green leafy vegetable curry: Refers to any gravy preparation that includes any green leafy vegetable such as amaranth, palak, etc., with or without dhal.
13.5 Rasam, all types: Refers to all types of rasam e.g. tamarind and tomato rasam.

CHUTNEYS / SALAD / PAPAD

14.1 Raw vegetable salad: All types of fresh salad prepared with one or more vegetables (e.g. cucumber, carrots, lettuce, onions, tomato etc)

14.2 Vegetable Raitha: Prepared with vegetables (e.g. onions, tomatoes, cucumber etc) and fresh curds.

14.3 Pickle: Includes all types of pickles used.

14.4 Papad: Refers to papads fried in oil. Papads are thin crisp sun-dried disc shaped wafers of dhal or cereal flour.

14.5 Coconut chutney: Refers to chutneys prepared with mainly coconut.

14.6 Groundnut chutney: Refers to chutney prepared with mainly groundnut.

14.7 Tomato chutney: Refers to chutney prepared with mainly tomato.

NON – VEGETARIAN

15.1 Chicken curry: Refers to chicken prepared in gravy from.

15.2 Chicken fry/grilled: Chicken fried or grilled form.

15.3 Mutton/ pork/beef curry or fry: Refers to all red meats in gravy form, fried or grilled form.

15.4 Fish curry: Refers to fish prepared in gravy from

15.5 Fish fry: Fish fried or grilled form

15.6 Organ meats (Liver, brain, kidney etc.): Refers to preparations containing these organs.

15.7 Prawn, crab, shell fish etc.: Preparations made with prawn, crab, shrimp or any shell fish.

15.8 Egg (boiled, poached, omelettes): Eggs prepared in any form – boiled, fried, poached, omelette, egg gravy, scrambled.

MILK & BEVERAGES

16.1 Tea: a. Refers to tea prepared with milk. b. Tea without milk

16.2 Coffee: a.Refers to coffee prepared with milk. b.Coffee without milk

16.3 Plain milk: Refers to plain milk with sugar without any other additions.

16.4 Flavored milk (horlicks, bournvita etc): Refers to milk with additions/supplements such as bournvita, milo, cocoa, horlicks, badam milk, milk shake etc.

16.5 Curds: Fermented milk

16.6 Buttermilk/Lassi: Diluted beaten curds with or without seasonings.

16.7 Lime/ orange/ other fresh fruit juice: Refers to any fresh fruit juice.

16.8 Fanta, pepsi, coca cola etc.: Refers to all aerated soft drinks available in bottles or cans.

16.9 Beer: Includes all forms of beer such as draught beer, etc.

16.10 Spirits (whiskey, gin, rum): Includes all other alcoholic beverages such as rum, whisky, vodka etc.

16.11 Other local alcoholic drinks: Refers toddy/arrack or any other locally available fermented alcohol taken from palm tree or any other source.

MISCELLANEOUS

17.1 Ghee/ butter: Refers to the addition of ghee or butter to any foods while eating (e.g. bread with butter, ghee with rice, idly etc.)

17.2 Jam: Includes all jams and marmalades (e.g. Kissan)

17.3 Sugar: Refers to additional sugar added to tea or coffee regularly.

17.4 Salt: Refers to any additional salt added to food regularly.

SNACKS/ SWEETS/DESSERTS

18.1 Mixture, namkeen, chiwda, khara boondi, dalmoth: Refers to all deep-fried salted snacks prepared from besan and cereal flour.
18.2 Vada, all types
18.3 Nuts (grounduts, cashewnuts etc.): Includes all nuts such as groundnuts (moongphali, kadalekayi), badam, cashewnuts, pista, walnut (akrut) etc.
18.4 Chips/ salted packed snacks (bingo, kurkure etc): Includes all types of chips and French fries.
18.5 Samosa,bajji ,bonda, cutlet, patties: Includes all deep-fried items with potatoes and/or vegetables dipped in batter or filled in a dough.
18.6 Salted biscuits (krackjack, bakery biscuits): Refers to all forms of salted biscuits such as krackjack, monaco, bakery salt biscuits etc.
18.7 Sweet biscuits (Marie/goodday/cream biscuits): Refers to all forms of sweet biscuits such glucose biscuits, marie, coconut, cookies, cream biscuits etc.
18.8 Murukku , chakli, sakinalu: Refers to deep fried savories prepared with any dhal and/or cereal flour made into a dough and fried in different shapes (usually rounds or circles)
18.9 Cakes or sweet pastries: Includes all cakes – plain or cream- and pastries like Black Forest etc.
18.10 Payasam, kheer: A sweet dish with cereal (sometimes with or without dhal) cooked in milk.
18.11 Ice cream: Refers to all ice creams
18.12 Jamoon, Jilebi, Jangir etc.: Refers to all fried sweet preparations dipped in sugar syrup.
18.13 Mysore pak, ladoo, barfis: Refers to all Indian besan and/or cereal based sweet preparations shaped into small squares/rectangles or round balls.
18.14 Baksham: Sweets prepared using cereal and/or dhal flour.
18.15 Dried fruits (dates, figs, raisins etc): Refers to all dried fruit
18.16 Chocolates: Includes all chocolates available in the market.

FRUITS

19.1 Banana
19.2 Apple
19.3 Orange
19.4 Mango
19.5 Guava (amrood): Amrood, Seebe, Peru, Jami pandu
19.6 Grapes (angoor): Angoor, draksha
19.7 Pineapple: Ananas
19.8 Papaya (papita)
19.9 Pomegranate (anar) : Anar, Dalimbari, Danimma pandu
19.10 Sapato (chikoo)
19.11 Watermelon (tarbooj) : Tarbooj, Kallangadi, Puchakayi
19.12 Musk melon (kharbooj) : Kharbooj
19.13 Custard apple: Seethaphal
19.14 Zizyphus (ber): Bor, Ber, Regu pandu
19.15 Sugarcane (ganaa)
19.16 Palmyra: Tar, Thati nungu, Thati pandu

VEGETABLES

20.1 Palak, methi, other leafy vegetables: Sag/ soppu. Includes all types of locally available green leafy vegetables.

20.2 Potato, sweet potato

20.3 Beetroot/ radish/ knol-khol: Includes beetroot, radish/turnip/mooli/shaljam, knol-khol

20.4 Cabbage

20.5 Beans, cluster beans: Includes French beans/semifalli, cluster beans/gowarfalli, broad beans/avarakkai

20.6 Ladies finger: Okra/ bhendi/ benda kayi/ bende kayi.

20.7 Cauliflower

20.8 Bottlegourd(lauki),ashgourd,Ridgegourd(turai), snakegourds, etc.: Includes all gourds like bottle gourd/dudhi/sorekai/kaddu/ sorekayi, ashgourd/petha/budagumbala kayi, ridge gourd/turai/heeraikayi, beera kayi, snake gourd/padavala, bitter gourd/karela/hagal kayi/kakara kayi.

20.9 Brinjal

20.10 Kovai

20.11 Capsicum or green pepper

20.12 Drumstick: Mungana phalli

20.13 Raw plantain

20.14 Colacasia (arvi): Colacasia/arvi/samagadde/chama dumpa

ENTRY OF RESPONSES FOR THE QUESTIONS (QUESTIONS 21.1 to 21.8):

21.1. Record the type used most often for the whole family.

21.2. Only number of coconuts used for the family per month is to be entered.

21.3. Definition of the different milks available:

Whole Milk

Includes all fresh milk given by vendors and supplied by milk booths. Whole milk can be only cow's milk OR milk with a combination of cow's milk and buffalo milk OR buffalo milk. It also includes whole milk powders and long life milk available in the market.

Products available:

Red packet government dairy milk
Heritage
Jersey
Nestle, Amul and Vijaya liquid whole milk and whole milk powder

Skimmed milk

Includes whole milk from which cream has been removed at home and the liquid skimmed milk available in the market.

Products available:

Nestle and Amul skimmed milk
Blue packet government milk

Toned milk

Includes dried skim milk added to a high –fat milk such as buffalo milk ,to reduce the fat content but maintain the total solids.

Products available:

Green packet government dairy milk

Skim milk powder:

Products available:

Sagar non fat milk powder .
Anikspray
Milk man dairy whitener (Britannia)-18% fat
Milkana –19% fat
Amulya dairy whitener – 20 g fat

21.4 Vitamins and mineral tablets or capsules if taken should be documented. 1=yes and 2=no.
If yes, details of the tablet/capsule taken should be documented.

21.5 Record if the subject perceives himself/herself to be a vegetarian.

21.6 This question is for people who have consciously adopted a diet that is different from their normal household diets for health or personal reasons.
a-e: If any special diets are taken, for example low fat diet, diabetic diet etc, then it should be documented as '1'=yes and '2' = no.
f: Any other diet apart from that listed in question12.10 is entered here.
h: The number of years the subject is on this type of diet is documented.

MEDICAL HISTORY

	Now I am going to ask you questions about your family history of illness, and your medical history					**COMMENTS**
	**Medical history**					
**22.16**	Is your father still alive?	(a) ☐ [1=Yes; 2=No]		(b) *If no, his* age at death ☐☐ [years]		Leave b blank if a is no.
**22.17**	(a) *If no*, what was the cause of his death?	☐ 1=Heart disease	2=high blood pressure	3=stroke	4=lung	Leave blank is father still alive
		5=cancer	6=accident/injury	7=other	8=don't know	
	(b) If "other" specify:					
	Did/does your father suffer from any of the following?					
**22.18**	Diabetes	☐ [1=Yes; 2=No; 3=Don't know]				
**22.19**	High blood pressure	☐ [1=Yes; 2=No; 3=Don't know]				
**22.19a**	Stroke	☐ [1=Yes; 2=No; 3=Don't know]				
**22.20**	Heart disease	☐ [1=Yes; 2=No; 3=Don't know]				

**22.21**	Overweight/obesity	☐ [1=Yes; 2=No; 3=Don't know]		
**22.22**	Lung disease	☐ [1=Yes; 2=No; 3=Don't know]		
**22.23**	Is your mother still alive?	(a) ☐ [1=Yes; 2=No]	(b) *If no,* her age at death ☐☐ [years]	Leave b blank if a is no.
**22.24**	(a) *If no*, what was the cause of his death?	☐ 1=Heart disease 2=high blood pressure 3=stroke 4=lung 5=cancer 6=accident/injury 7=other 8=don't know		Leave blank is mother still alive
	(b) If "other" specify:			
	Did/does your mother suffer from any of the following?			
**22.25**	Diabetes	☐ [1=Yes; 2=No; 3=Don't know]		
**22.26**	High blood pressure	☐ [1=Yes; 2=No; 3=Don't know]		
**22.26a**	Stroke	☐ [1=Yes; 2=No; 3=Don't know]		
**22.27**	Heart disease	☐ [1=Yes; 2=No; 3=Don't know]		
**22.28**	Overweight/obesity	☐ [1=Yes; 2=No; 3=Don't know]		
**22.29**	Lung disease	☐ [1=Yes; 2=No; 3=Don't know]		
	Did/do any of your brothers or sisters suffer from any of the following?			Answer for any brothers or sisters.
**22.30**	Diabetes	☐ [1=Yes; 2=No; 3=Don't know; 4=no brothers]		
**22.31**	High blood pressure	☐ [1=Yes; 2=No; 3=Don't know; 4=no brothers]		
**22.31a**	Stroke	☐ [1=Yes; 2=No; 3=Don't know; 4=no brothers]		
**22.32**	Heart disease	☐ [1=Yes; 2=No; 3=Don't know; 4=no brothers]		
**22.33**	Overweight/obesity	☐ [1=Yes; 2=No; 3=Don't know; 4=no brothers]		
**22.34**	Lung disease	☐ [1=Yes; 2=No; 3=Don't know; 4=no brothers]		
**23.1**	(a) Have you had wheezing or whistling in your chest at any time in the last year?		☐[1=Yes; 2=No]	These questions try to assess whether the participant has asthma.
	If yes, (b) In the last year have you had this wheezing or whistling only when you have a cold?		☐[1=Yes; 2=No]	
	(c) In the last year have you ever had an attack of wheezing or whistling that has made you feel short of breath?		☐[1=Yes; 2=No]	
**24.1**	(a) Do you usually cough when you don't have a cold? **[If no, skip to 24.2]**		☐ [1=Yes; 2=No]	**COMMENTS**
	If yes (b) Are there months when you cough most days		☐ [1=Yes; 2=No]	Skip if answer to a is no
	(c) Do you have a cough on most days for as much as three months each year?		☐ [1=Yes; 2=No]	
	(d) For how many years have you had this cough?		☐☐ [Years]	
**24.2**	(a) Do you usually bring up phlegm from your chest, or do you usually have phlegm in your chest that is difficult to bring up when you don't have a cold? **[If no, skip to 24.3]**		☐ [1=Yes; 2=No]	
	If yes, (b) Are there months in which you have this phlegm on most days?		☐ [1=Yes; 2=No]	Skip if answer to a is no

	(c) Do you bring up this phlegm on most days for as much as 3 months per year?	☐ [1=Yes; 2=No]	
	(d) For how many years have you had this phlegm?	☐☐ [Years]	
**24.3**	(a) Are you unable to walk due to a condition other than shortness of breath? **[If no, skip to 24.4]**	☐ [1=Yes; 2=No]	Examples include paralysis, amputations
	(b) *If yes*, name of condition_____		
**24.4**	**If able to walk:** (a) Are you troubled by shortness of breath when hurrying on the level or walking up a slight hill? **[If no, skip to 24.5]**	☐[1=Yes; 2=No]	
	If yes,<br>(b) Do have to walk slower than most people of your age on level ground because of shortness of breath?	☐[1=Yes; 2=No]	Skip if answer to a is no
	(c) Do you have to stop for breath when walking at your own pace on level ground?	☐[1=Yes; 2=No]	
	(d) Do you ever have to stop for breath after walking about 100 yards on level ground?	☐[1=Yes; 2=No]	
	(e) Are you too short of breath to leave the house or short of breath on dressing of undressing?	☐[1=Yes; 2=No]	
**24.5**	(a) Have you ever had any pain or discomfort in your chest? **[If no, end section]**	☐ [1=Yes; 2=No]	
	If yes,<br>(b) Do you get it when you walk uphill or hurry?	☐ [1=Yes; 2=No]	
	(c) Do you get it when you walk at an ordinary pace on the level?	☐ [1=Yes; 2=No]	
	**If no pain on walking, end section. Otherwise ask d-g**		
	(d) What do you do if you get it while you are walking?	☐ 1=Stop/slow down; 2=Carry on	
	(e) If you are standing still, what happens to it?	☐ 1=Relieved<br>2=Not relieved	
	(f) How soon?	☐1=10 minutes or less; 2=Over 10 minutes	
	(g) Will you show me where it is (record all places)? [SHOW PICTURE]	☐, ☐, ☐, ☐	Mark all the points where pain is felt

ANTHROPOMETRY MEASUREMENT

	**Weight and height**	**a) First reading**	**b) Second reading**	
**25.1**	Weight	☐☐☐ . ☐ [kg)	☐☐☐ . ☐ [kg]	To 0.1 kg (100 g)
**25.2**	Scale number	☐		
**25.3**	Standing height	☐☐☐☐ [mm]	☐☐☐☐ [mm]	To 1 mm (0.1 cm)

**25.4**	Sitting height	☐☐☐☐ [mm]	☐☐☐☐ [mm]	To 1 mm (0.1 cm)
**25.5**	Stool height	☐☐☐☐ [mm]	☐☐☐☐ [mm]	To 1 mm (0.1 cm)
**25.6**	Stadiometer number	☐		
	**Circumferences**	**a) First reading**	**b) Second reading**	
**25.7**	Waist circumference	☐☐☐☐ [mm]	☐☐☐☐ [mm]	To 1 mm (0.1 cm)
**25.8**	Hip circumference	☐☐☐☐ [mm]	☐☐☐☐ [mm]	To 1 mm (0.1 cm)
**25.9**	Mid-arm circumference	☐☐☐☐ [mm]	☐☐☐☐ [mm]	To 1 mm (0.1 cm)
**25.10**	Calf circumference	☐☐☐☐ [mm]	☐☐☐☐ [mm]	To 1 mm (0.1 cm)
**25.11**	Head circumference	☐☐☐☐ [mm]	☐☐☐☐ [mm]	To 1 mm (0.1 cm)
**25.11i**	Chest circumference at end-inspiration	☐☐☐☐ [mm]	☐☐☐☐ [mm]	To 1 mm (0.1 cm)
**25.11ii**	Chest circumference at end-expiration	☐☐☐☐ [mm]	☐☐☐☐ [mm]	To 1 mm (0.1 cm)

**25.12**	Triceps skinfold	☐☐.☐ [mm]	☐☐.☐ [mm]	☐☐.☐ [mm]	To 0.2 mm
**25.13**	Biceps skinfold	☐☐.☐ [mm]	☐☐.☐ [mm]	☐☐.☐ [mm]	To 0.2 mm
**25.14**	Subscapular skinfold	☐☐.☐ [mm]	☐☐.☐ [mm]	☐☐.☐ [mm]	To 0.2 mm
**25.15**	Suprailiac skinfold	☐☐.☐ [mm]	☐☐.☐ [mm]	☐☐.☐ [mm]	To 0.2 mm
**25.16**	Calf skinfold	☐☐.☐ [mm]	☐☐.☐ [mm]	☐☐.☐ [mm]	To 0.2 mm
**25.17**	Caliper number	☐			

	**Muscle strength**	**Reading1**	**Reading2**	**Reading3**	**Reading4**	
**25.17**	Right hand	☐☐.☐ [kg]	☐☐.☐ [kg]	☐☐.☐ [kg]	☐☐.☐ [kg]	To 0.5 kg
**25.18**	Left hand	☐☐.☐ [kg]	☐☐.☐ [kg]	☐☐.☐ [kg]	☐☐.☐ [kg]	To 0.5 kg
**25.19**	Dominant hand	☐ [1=Right, 2=Left]				Hand usually used, e.g. for writing
**25.20**	Grip strength machine	☐				

	General information: anthropometry measurements		
**25.22**	Researcher code	☐☐	Each interviewer given codes starting from '1' to each of the team members
**25.23**	Researcher initials	☐☐☐	These should decided at the start of the study and used consistently (e.g. don't flip between 2/3 alphabets
**25.24**	Left sided measurements	☐ [1=Yes; 2=No]	
**25.25**	If not, specify	_____	
**25.26**	All measurements adequate	☐ [1=Yes; 2=No]	Adequacy refers to any problems in taking the reading that could affect their validity e.g. bandage or any obvious deformity. Where problems arise, every effort should be made it overcome them (i.e. remove bandage if possible) rather than simply proceeding with recording as such measurements are largely useless.
**25.27**	If not, specify	_____	

Anthropometry Protocol

The methodology for anthropometrical assessments was adapted from standard reference texts on the subject.

General principles

- A digital weighing machine (Seca scales) with an accuracy of 100 gms will be used to measure weight.
- Height will be measured by a portable plastic stadiometer with a base plate, accurate to 1 mm (Seca Leicester height measure).
- For sitting height, a standard table (of known height) high enough for the legs of the participant to dangle over the edge when seated will be used.
- A skinfold calliper accurate to 0.2 mm (Holtain skinfold calliper;) will be used to measure the thickness of the skinfolds.
- Lengths and circumferences will be measured with a non-stretch metallic tape with a narrow blade and a blank lead-in (ADE metallic tape;).
- The measurements (where relevant) will be made on the **left side**. If for some reason this is not possible, measurements should be taken on the opposite side and this change recorded.
- Measurements should be conducted in normal indoor clothing (basic clothes minus shoes, sweaters, socks, etc).
- Weight and height will be recorded twice, circumferences twice and skinfolds readings will be taken three times.

Notes:

(a) Extreme care should be taken to avoid digit preference when taking the recordings

(b) Any problems at the time of measurement that can affect their accuracy (such as those due to physical deformities, postural problems or bandages on the limbs) are recorded. However, in many cases the problem may be partially rectifiable e.g. removal of bandage in case of one. A common problem is that of hunching in case of older subjects. If so, check if maximum of the back is in contact with the wall. As the inadequate measurements cannot be used, it is strongly advisable to try and address the problem rather than record inadequate measurements.

Weight

The weighing machine should be placed on the most level part of the floor and calibrated at the start of every clinic. At the time of each reading, the scale should be turned again to ensure that the monitor reads 'zero'. The participant should be asked to stand on the scale reasonably straight and looking ahead. Weight should be recorded only when the reading on the monitor had settled. Avoid taking the reading as the participant is coming off the weighing machine.

Height and sitting height

The stadiometer should be set up on the most level part of the floor and calibrated at the start of every clinic. Where the floor is not level (as detected with the help of a spirit level), a suitable standard wooden block with appropriate wedges set into place should be used to deal with the faulty floor. The stadiometer is then placed on this block and measurements done as stated below.

The participant should be asked to stand on the stadiometer, while the observer checks the following points:

(a) feet flat on the centre of the base plate, ankles together, heels resting on the bar at the back, and the inner borders of the feet at an angle of 60 degrees;

(b) Back as straight as possible, preferably against the rod but not leaning on it;

(c) Arms resting by sides, not behind or in front; and

(d) Head in the Frankfurt plane (eyes looking straight ahead such that the lower edge of orbit was in line with external auditory meatus i.e. ear hole).

The participant should be instructed to keep his/her eyes focused on a point straight ahead and to breathe easily. The headrest should be lowered while checking at the same time that the participant does not stand on tiptoe. The observer should be level with the scale at the time of reading to avoid errors due to parallax. To ensure this, the observer may have to stand on a stool or a thick book.

The table should be set up on a firm flat surface ensuring that it is stable and horizontal. The participant should be asked to sit on the table, and the measurement of height repeated using the same procedure as described for height above: head in the Frankfort plane, the back straight, and the thighs horizontal so that the hollow of the knee is approximately 1 cm clear of the table. The participant should be sitting on the table with the legs hanging unsupported over the edge, hands resting on the thighs with the buttocks and shoulders relaxed. The participant should be asked to sit up tall and headpiece slid down until it touches the head.

Waist circumference

The participant should be asked to stand straight with feet close together and abdomen relaxed, weight evenly balanced on both feet, and the arms hanging loosely at the sides. The measurement should be carried out on the bare skin. The observer should face the participant and place the tape around the participant, in a horizontal plane, at the level of the natural waist, which is the narrowest part of the abdomen between the ribs and the iliac crest (top of the hip bone), as seen from the anterior aspect. With the participant breathing out gently looking straight ahead, the reading should be taken at the end of a normal expiration.

General notes for measuring circumferences:

Circumferences should be recorded with the zero end of the tape held in the left hand above the remaining part of the tape held by the right hand. The plane of the tape should be perpendicular to the long axis of that part of the body, and parallel to the floor. For all circumference measurements, the tape should not be pulled too loose or too tight, held snugly around the body part but not indenting it. In some individuals there may be gaps between the tape measure and the skin, in such cases attempting to reduce the gap by increasing the tension of the tape is not recommended.

Hip (buttock) circumference

The measurement is carried out with the participant wearing normal indoor clothing. The tape measure is applied to the widest part of the buttock. This is ensured by the observer squatting at the side of the participant so that the level of the maximum extension of the buttocks can be seen. Attention should be paid that the hip muscles of the participant are not contracted, and the tape is horizontal and not compressing the skin.

Mid-arm circumference

The participant should stand with his/her back to the observer with the arm flexed at 90 degrees. The tip of the acromion (the point of the shoulder) is palpated and marked. Then with the participant's arm flexed at 90 degree, the olecranon (tip of the elbow) is palpated. The tape measure should be put on the mark on the shoulder and dropped down to the tip of the elbow by the side of the arm. The exact distance is read as if an imaginary horizontal line had been drawn from the bottom most point of the elbow to the tape measure, and a point halfway between the acromion and the olecranon marked. One way of doing this to measure the length as above and then fold the tape on to itself to identify the mid-point. The participant is then asked to relax, with the arm hanging by the side. The tape is placed around the upper arm such that its upper border was at the level of the marking. The tape should be horizontal all round, resting firmly on the skin but not indenting it.

Calf circumference

The calf circumference is measured with the participant standing, evenly balanced, with the feet shoulder width apart. The measurement should be taken on bare skin. The tape is positioned horizontally around the calf and moved up and down to locate the maximum circumference in a plane perpendicular to the long axis the calf. The observer should bend down at the time of the reading to avoid errors due to parallax. The level at which the measurement is taken should be marked at the medial aspect of the calf (inner border).

Head circumference

The head circumference is measured with the subject standing. Added objects, for example, pins are removed from the hair. The measurer stands facing the left side of the adult. The tape is passed around the head and positioned so that large amounts of cranial hair (braids) are excluded. Anteriorly, the tape is placed just superior to the eyebrows and posteriorly it is placed so that the maximum circumference is measured. The tape is pulled tightly to compress hair. The measurement is recorded to the nearest 0.1 cm.

Chest circumference

The chest circumference is measured with the subject breathing normally. The tape should be held horizontally at the level of the nipples, passing over the lower scapular angle. The arm of the subject may be raised before fixing the tape around the chest, but after that the arms should rest normally, by the side, while taking the measurement. In case of females, another circumference at the base of the xiphoid process, horizontal to the thorax may be taken.

Chest girth inspiration: The subject should be allowed to draw his breath in as much as possible and note the highest value.

Chest Girth Expiration: The subject should be asked to breathe out and the least value is noted.

General notes for measuring skinfolds*:* The sites should be marked before taking these readings. Palpation of the site prior to measurements helps familiarise the participant with contact in the area. The callipers should be held in the right hand, while the thumb and the index finger of the left hand are used to elevate a double fold of skin and subcutaneous adipose tissue about 1 cm proximal to the site at which the skinfold will be measured. Place the thumb and the index finger on the skin about 3 inches apart on a line perpendicular to the long axis of the skinfold, and gently pull the skinfold away from the body by drawing the thumb and index finder towards each other. The fold needs to be grasped firmly, and the amount of tissue must be sufficient to form a fold with approximately parallel sides. Care must be taken so that only skin and adipose tissue are elevated (but not the muscle). The calliper head should perpendicular to the skinfold (with the dial facing), placed halfway between the crest and base of the fold. Gently and fully release the calliper pressure (the release of the pressure should be gradual to avoid discomfort). The pinch should be maintained for 5 seconds before taking the measurement. It is important to keep this timing accurate as otherwise the reading will vary. Errors due to parallax should be avoided by correct positioning over the dial (this may involve standing on something to be at the right height).

Triceps skinfold thickness

The triceps skinfold is measured in the midline of the posterior aspect of the arm, over the triceps muscle, at a point midway between the lateral projection of the acromion process of the scapula and the inferior margin of the olecranon process of the ulna (identified as for the mid-arm circumference). A cross is marked with a vertical line drawn on the most dorsal part of the arm determined by 'eyeballing' the midpoint (the part that sticks out furthest posteriorly), and the horizontal line the same as that drawn for the mid-arm circumference. The skinfold is measured with the participant standing and the arm hanging loosely and comfortably at the side. With the calliper held in the right hand, the observer stands behind the subject and places the palm of his/her left hand on the subject's arm proximal to the marked level, with the thumb and index finger directed inferiorly. The triceps skinfold is picked up with the left thumb and the index finger, approximately 1 cm proximal to the marked level, and the tips of the callipers are applied to the skinfold at the marked level. The readings should be taken 5 seconds after the application of the callipers jaws.

Biceps skinfold thickness

Biceps skinfold thickness is measured as the thickness of a vertical fold raised on the anterior aspect of the arm, over the belly of the biceps muscle. The skinfold is raised 1 cm superior to the line marked for the measurement of triceps skinfold thickness and arm circumference, on a vertical line joining the anterior border of the acromion and the center of the antecubital fossa. The subject stands, facing the measurer, with the upper extremity relaxed at the side and the palm directed anteriorly. The calliper jaws are applied at the marked level. The thickness of the skinfold is recorded to the nearest 0.1 cm.

Subscapular skinfold thickness

The lowermost tip of the scapula is identified. If it is difficult to appreciate this, the participant should be asked to place the back of his/her hand on the lumbar region. The medial border of the scapula is followed downwards until the inferior angle is felt. Once it is identified, the participant was asked to relax with arms hanging by the side before a mark is applied to the skin immediately below the lower most tip (angle) of the scapula. The skinfold is picked up obliquely above the mark with the fold slightly inclined downward and laterally (at about 45 degree to horizontal), in the natural cleavage of the skin. The calliper jaws are applied below the fingers, such that the marked cross is at the apex of the fold. Readings should be taken after 5 seconds.

Suprailiac skinfold thickness

The suprailiac skinfold is measured in the anterior axillary line immediately superior to the iliac crest. The subject stands with feet together and in an erect position. The arms hang by the sides. An oblique skin fold is grasped just posterior to the anterior axillary line following the natural cleavage lines of the skin. It is aligned inferomedically at 45 ^{o} to the horizontal. The calliper jaws are applied about 1 cm from the fingers holding the skinfold, and the thickness is recorded to the nearest 0.1 cm.

Calf medial skinfold thickness

The participant stands with the foot of the side being measured on a platform, so that the knee and hip are flexed to about 90 degrees. The skinfold is measured at the level of the maximum calf circumference, already marked as before. From a position in front of the participant, the observer raises a skinfold parallel to the long axis of the calf on its medial aspect, when viewed from the front, at a level slightly proximal to the marked site. The calliper head should be perpendicular to the skinfold with the dial facing up at the marked point on the medial border of the calf.

Grip strength

1. Before this test is administered, the handle of the dynamometer must be adjusted for the size of each individual subject. The handle should fit comfortably in the hand with enough allowance for a good grip. Record the setting found on the inside gauge if follow-up testing is to occur.
2. Place the subjects arm at their side keeping it away from the body with the elbow bent slightly (approximately 20^{0}). Illustrate the use of the instrument to the subject prior to testing.
3. The test is to be administered with dominant hand first and then with the non-dominant hand. The examiner should be confident that the subject's maximum grip strength is being measured. Emphasis on "squeeze as hard as you possible can" and other forms of encouragement may be necessary for maximum effect. Allow three trials with each hand, right and left hand alternately, but introduce a brief pause of about 10 to 20 seconds between each trial to avoid excessive fatigue.

4. Record the amount of strength registered at each trial. If the difference between the scores of each is within 3 kilograms, (considering the scores of each hand separately) the test is complete. If a difference of more than 3 kilograms is noted, the test is repeated after a sufficient rest period. Calculate the averages for each hand separately. It is important that the dials be returned to the “0” position after every trial. Readings are taken to the nearest whole kilogram.

Blood Pressure

	Blood pressure			
**26.1**	Room temperature	☐☐ . ☐ [degree Celsius]		
		a) First measure	b) Second measure	b) Third measure
**26.2**	Systolic BP (brachial)	☐☐☐ [mmHg]	☐☐☐ [mmHg]	☐☐☐ [mmHg]
**26.3**	Diastolic BP (brachial)	☐☐☐ [mmHg]	☐☐☐ [mmHg]	☐☐☐ [mmHg]
**26.4**	Pulse rate	☐☐☐ [bpm]	☐☐☐ [bpm]	☐☐☐ [bpm]
**26.5**	Cuff size used	☐ [1=Small; 2=Medium; 3=Large]		
**26.6**	BP apparatus number	☐		
**26.7**	Right arm measurements	☐ [1=Yes; 2=No]		
**26.8**	Measurements adequate	☐ [1=Yes; 2=No]		
**26.9**	If not, specify	_____		

Blood pressure measurement

Blood pressure (BP) will be measured with OMRON HEM 7300 (Omron, Matsusaka Co., Japan). This instrument meets the AAMI and International Protocol accuracy criteria. Three sized cuffs are available: (a) small cuffs (arm circumference 15-22 cm), (b) normal cuff (arm circumference 22-32 cm); and extra large cuff (arm circumference 32-42 cm). A digital thermometer (model name, company, country) will be used to measure room temperature. BP will be measured in right upper arm in sitting position.

Note: All those expected to be involved in the BP measurements should go through the instruction manual beforehand to familiarise themselves with the issues, possible problems and care of the equipment.

Steps in blood measurement

1) Check the subject has not undertaken following activities for at least 30 minutes preceding the examination: strenuous exercise, eating, drinking of anything other than water, smoking or drugs that affect the BP. Ask the subject to sit on the chair with right arm on the table. *Important:* Upper arm (cuff when applied) should be at the level of the heart, neither too high nor low. If required use a pillow or some other support to ensure this.

2) Apply the correct size cuff. This should be determined by the arm circumference (see above), which should be read from the anthropometry section. You should also keep a measuring tape handy to check in case the subject has not been to the anthropometry section (in case you do measure the arm circumference for assessment of the cuff size, avoid the temptation to note it in the anthropometry datasheet at this stage). The green coloured band (indicating the centre of the bladder) should be positioned 1-2 cm above the elbow joint on the inside of the arm. Close the cuff with the fabric fastener. The green area of the cuff must cover the brachial artery. The cuff should not be too tight or too loose; it should fit snugly so that it is just possible to fit two fingers between the arm and the cuff. Do not place the cuff on thick clothes or roll up the sleeve if it is too tight. *Important:* The sleeve of the dress should not constrict the blood flow in the arm. If so (either because of tight fitting shirt/blouse or rolling up of tight sleeve), take steps to address this (e.g. asking the subject to take off shirt over that arm or in case of female subject provide them with a loose gown to change into).

3) After the cuff is applied, wait for five minutes before taking the first reading. The subject should be made to relax during this time by making them sit comfortably, asking them to breathe easily and to relax the body and arm, and explaining the procedure. Stress that they should not move, talk or touch the device during measurement. This period should also be used to note down information in the datasheet: researcher code and initials, BP apparatus number, cuff size used, and arm side on which the measurement is being made. *Important:* The BP apparatus will give accurate readings at temperature range of 10 – 40 degree Celsius. If room temperature is outside this range, take steps to address this (use room heater if too cold or cooler or table fan with cloth soaked in water if cooler not available if too warm).

4) Press the START button on the machine. Wait for the machine to complete the measurement. This is confirmed by the flashing of a single downward arrow symbol. When all the air has been released the 'heart' symbol on the display panel and the readings are displayed for the next five minutes. Note down the systolic and diastolic blood pressure and the pulse rate. *Important:* If the subject moves or talks during the measurement or the measurements appear inaccurate or the symbol 'E' (indicating error) is displayed on the screen, repeat the measurement.

5) Wait for 1 minute before taking the second reading so that blood circulation can resume. This can be ensured by presence of 'heart' symbol on display panel. If you need to interrupt a measurement for any reason, you can do so by pressing the ON/OFF button. Record the second set of readings on the datasheet. Note down any problems (such as subject appearing too anxious despite reassurance or if the readings had to be taken on the left arm for some reason). Switch off after measurement to conserve batteries. The monitor will also switch off automatically after five minutes.

Respiratory function

	**Respiratory function**	
**27.1a**	In the past three months have you had any surgery on your chest or abdomen?	☐ [1=Yes; 2=No]
**27.1b**	Have you had a hernia problem at any time?	☐ [1=Yes; 2=No]
**27.2**	Have you had a heart attack within the past three months?	☐ [1=Yes; 2=No]
**27.3**	Do you have a detached retina or have you had eye surgery within the past three months?	☐ [1=Yes; 2=No]
**27.4**	Have you been hospitalized with any other heart problem within the past month?	☐ [1=Yes; 2=No]
**27.5**	Are you in the last trimester of pregnancy?	☐ [1=Yes; 2=No]
**27.6**	Are you currently taking medication for TB?	☐ [1=Yes; 2=No]
**27.7**	Have you coughed up blood within the past month?	☐ [1=Yes; 2=No]
**27.8**	Does the participant have a resting pulse of greater than 120 beats per minute?	☐ [1=Yes; 2=No]

	**If any of the questions 27.1 to 27.8 is "yes", do NOT proceed with the test**					
**27.9**	(a) Have you taken medication for breathing in last 6 hours?					☐ [1=Yes; 2=No]
	If yes, name of medication: _____					
**27.10**	Have you had a respiratory infection (cold) in the last three weeks?					☐ [1=Yes; 2=No]
	**TAKE VERBAL CONSENT TO DO THE TEST**					
		**a) Blow 1**	**b) Blow 2**	**c) Blow 3**	**d) Blow 4**	**e) Blow 5**
**27.11**	FEV_1	☐.☐☐[l]	☐.☐☐[l]	☐.☐☐[l]	☐.☐☐[l]	☐.☐☐[l]
**27.12**	FVC	☐.☐☐[l]	☐.☐☐[l]	☐.☐☐[l]	☐.☐☐[l]	☐.☐☐[l]
**27.13**	If unable to obtain satisfactory spirometry (check one):			☐ 1 = Participant did not understand instructions<br>2 = Participant medically excluded<br>3 = Participant unable to physically cooperate<br>4 = Participant refused		

Spirometry test

Pulmonary Function Assessment (PFTs)

The PFTs will be measured using the Card Guard SpiroPro device-a portable spirometer that accurately measures lung ventilatory functions during Vital Capacity (VC) and Forced Vital Capacity (FVC) tests. The system records the Volume-time and Flow-volume curves.

Verbal consent

1. Step 1. Before administering the test, explain to the participant that you are going to ask them to do a breathing test, which involves them breathing into a tube. Ask if they are happy with this. If they say yes, then proceed with the questionnaire. If they say no, then explain test further and attempt to persuade them.

Respiratory function questionnaire

2. Step 2. Administer respiratory function questionnaire (below). If any of questions 23.1 to 23.8 is "yes" do not proceed with test.

Preliminary checks

3. Step 3. Check person has passed urine before test.
4. Step 4. Check weight and height without shoes are recorded.
5. Step 5. Wash hands.
6. Step 6. Check participant is sitting upright on chair with both feet flat on floor.

Describe spirometry test

7. Describe spirometry test to participant. Say to the participant:
"After breathing normally, slowly blow out until your lungs are empty.
Then take a big deep breath in filling your lungs completely.
Place the mouthpiece inside mouth and close lips tightly around the mouthpiece
Blow out as hard and as fast as you can.
Keep going until you cannot push anymore air out."

8. Step 8. Fieldworker demonstrates test with own mouthpiece.
   a) Move the switch to 'BLOW'
   b) Attach nose clip with new piece of plastic
   c) Demonstrate the test
9. Step 9. Check that participant understands test

Participant performs spirometry test.

10. Participant performs spirometry test with new mouthpiece and new piece of plastic on noseclip. Fieldworker talks to them as they do test:
"Feet flat on the floor, sit upright….
Breathe out……
Deep breath in….
Mouthpiece inside mouth…
Breath out as hard and as fast as you can….
Keep going, keep going, keep going etc…..(encourage vigorously for 6 seconds)
And stop.
Relax
Well done"

Record test results

11. Step 11. Check if blow is acceptable. If any of the following happens the blow is **not** acceptable, so do not record, and repeat the blow.
    Unacceptable blow conditions
    - The mouthpiece is blocked
    - There is a leak around the mouthpiece
    - The mouthpiece does not go inside the mouth
    - The participant coughs during the blow
    - The effort is not maximal
    - The participant blows twice
    - The participant stops before 6 seconds
12. Step 12. If blow is acceptable then record results for Blow 1 on questionnaire
    a) FEV_1 will be shown on the display. Write this down.
    b) Move switch to the 'VIEW' position, FVC will be displayed. Write this down. Do not write down PEF and PER.

Repeat test for Blows 2 and 3

13. Step 13. Switch off unit and then back on again. Repeat test at least 2 more times and write down the FEV1 and FVC for blows 1, 2 and 3. Check if test is acceptable after each blow.

Criteria for stopping the test.

14. Step 14. After three acceptable sets of results have been obtained, check that the:
    - two largest values of FVC are within 0.15 L of each other
    - two largest values of FEV1 are within 0.15 L of each other
    - (Note these don't need to be from the same blows)

    If both of these criteria are met, stop the test.
    If both of these criteria are not met, perform a further two blows and write these down as Blows 4 and 5.

    Repeat blows until
    - Both of the above criteria are met
    - Or, a total of eight tests have been performed
    - Or, the subject cannot continue

15. Step 15. Discard mouthpiece at end of test.

<u>Potential problems which may affect test:</u>

- Not putting mouthpiece inside mouth (needs to go between the teeth but not so they bite on it)
- Embarrassment and laughing during test which makes effort not be maximal
- Oral phobia so refuses to put it in their mouth or cannot open mouth wide enough to do the test
- Stress incontinence in women so effort will not be maximal. Make sure people pass urine before the test to try and stop this happening.

<u>Other important points:</u>

- Fieldworkers must write their name on their mouthpiece and keep it for the duration of the day.
- Fieldworkers must write down only the FEV and FVC (do not record PEF and PER when they move the switch to VIEW position)
- Check spare batteries are available

Step-wise guide to using Card Guard Spiro-Pro

1. Ensure that your Spiro Pro device is bonded by Blue-tooth to a PMP^4 Application compatible PC/Laptop.
2. This ensures that the test data will not be displayed on the Spiro Pro screen, but on the laptop from where readings/graphs can be stored in a folder for each subject.
3. Start the PMP^4 Application by tapping the icon on the dedicated laptop.
4. Verify that Bluetooth is set for the PDA communication mode.
5. Verify that Bluetooth is set as the communication mode for PMP4 Application.
6. Enter given password.
7. Add patient –click on pictorial icon in bottom left.
8. Enter patient information –Last name, first name, Father's name and Mother's name, Birth date and patient ID-16 digit. Copy the ID to the next column as well. Click on Validate.
9. Add all remaining information, height and weight.
10. Click on "Save Patient".
11. Enter subject name in the search column...this should show up subject's name in the database. Click on synchronise.
12. Prepare the Spiro Pro by the following steps: a. Insert a disposable mouthpiece into the mouthpiece adapter of the flow meter. b. Hold the Self Check Spiro Pro by grasping the handle. c. Place the laptop with PMP ^{4} application in a position close by so that it is possible to access the device.
13. Start the test by clicking on the "Tests" button on the PMP^4 Application "Home" page. Note: In each screen you can use the PMP^4 application toolbar icons "Back" to return to the previous screen or "Home" to the main screen or select to exit the PMP^4 application by clicking on the "Exit" icon on the toolbar.
14. At this point, place the Spiro Pro device on a flat surface until the calibration (signalled by a beep) is finished. The PMP^4 Application start screen will prompt you to start the test by pressing the "start" (left) button on the Spiro and tap the PMP^4 Application check icon. The Spiro Pro will now start a calibration (a few seconds). Wait until the beep is finished before starting the test.
15. A Welcome screen will be displayed on the Spiro Pro.
16. A main screen then opens with a Bluetooth icon blinking (searching for the Bluetooth bonded device. Wait for the icon to stop blinking and for the next screen.
17. A heart will be displayed on the screen indicating it is ready for testing.
18. Instruct the subject to blow into the Spiro Pro mouthpiece as hard and long as possible.
19. The PMP^4 Application will display that the data is being transferred till the end if the test.
20. The test results are transmitted automatically and displayed on the screen of the PMP^4 Application.
21. To stop the test: Press the left button on the Spiro Pro device (turns off the device) or Tap the 'Close" icon in the PMP^4 Application screen.
22. At the end of the test the PMP^4 Application will display the final results on the screen.
23. At this point the user can return to "Home", use "Back' or "Display chart" options.
24. Save the test results by tapping the save icon.
25. This will open the comment screen, enter comments and again tap on the 'Save" icon.
26. Click on Confirm-test saved.
27. To check the spirograph again, go to medical history on L bottom, click on Spiro icon and click on show graph.

28. Save the graphs in the folder created for the subject.
29. The team leader will be responsible for sending the recordings in Batches of 50 to Dr. Raghupathy Anchala who will read the graphs and interpret recordings for the APCAPS study in consultation with Dr. Yoav Ben Shlomo and Dr. John Henderson in University of Bristol.

Spirometry flow chart

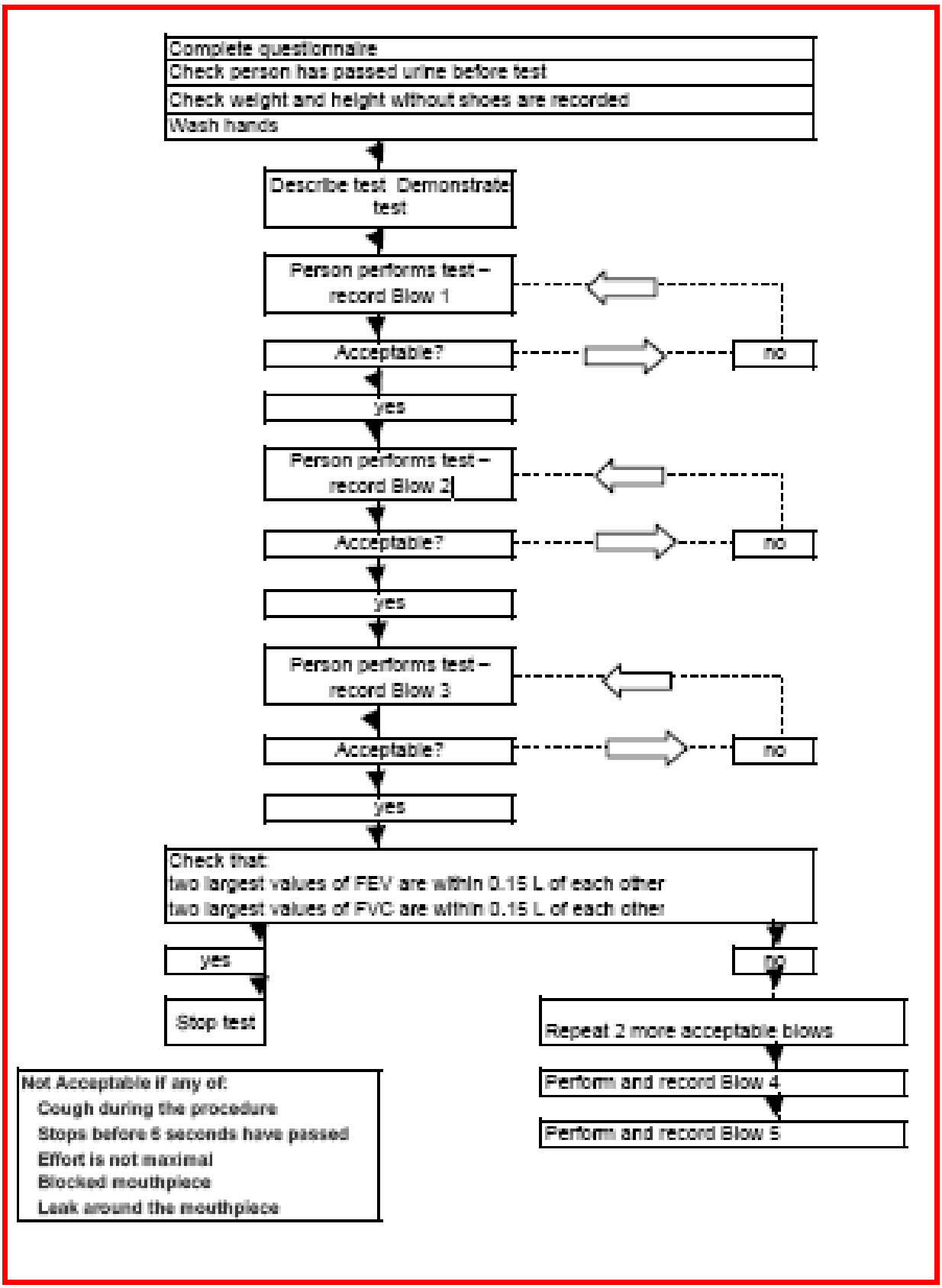

Instructions for Accelerometer

1. Check and set time on the computer so that accelerometer takes up the same time.
2. Accelerometer to be given when available to consenting subjects among parents and siblings.
3. Initialize the accelerometer right after download of the previous recording (no need to set a starting time)
4. Put belt on the subject at the end of the clinic itself.
5. Instruct subjects to keep the accelerometer on for 5 days and return.
6. Instruction sheet to explained to all the subjects and then given.
7. For illiterate subjects, sheet completed on return of the accelerometer by asking subject (approximate times should be okay).
8. Accelerometer to be given to those keen and reliable.
9. Give preference to repeats if available, at the same time not to wait.
10. Ensure that the accelerometer sheet is completed- record start/end times, times taken off and details of travel if person has travelled for more than 2 hours.

ACCELEROMETER INSTRUCTION SHEET

Accelerometer Number:_____ Study ID No _____

Name: _____ Weight _____kg

Sex: _____

Date of accelerometer applied: _____

Time of accelerometer applied: _____

[*The above to be filled by the team and needs to be entered at the end of clinic but could be different if subject insists on starting later at home in which case subject could fill it].*
Date of final accelerometer removal: _____

Time of final accelerometer removal: _____

[To be filled by team/subject and team to confirm the timings with the subject once again when they return the accelerometer].

Please ask the subject to keep the accelerometer on at all times, including during sleep. If they need to remove the accelerometer at any point, please ask them to fill the information in on the sheet below.

	**Date of removal**	**Time of removal**	**Date put back on**	**Time put back on**	**Reasons for removal**
1.					
2.					
3.					
4.					
5.					
6.					
7.					
8.					
9.					
10.					

Notes for special information:

(e.g. in cases where the subject has travel of more than 2 hours, please ask subject to specify timings of start and end of both way travel and also mode of travel).

ONLY FOR WOMEN

	Now I will ask you a few questions about your reproductive history ***(women only)***		
	**Reproductive history**		
**28.1**	At what age did your periods start?	☐☐ [Age in completed years]	Record age at first period
**28.2**	(a) Do you still menstruate?	☐ [1=Yes; 2=No]	
	(b) *If no*, at what age did your periods stop?	☐☐ [Age in completed years]	If (a) is yes, leave blank
	(c) *If yes,* do you have irregular/infrequent menstrual cycles?	☐ [1=Yes; 2=No]	If (a) is no, leave blank
	(d) *If yes,* how many periods do you have in a year?	☐☐ [Number]	If (a) is no, leave blank
**28.3**	Do you have excess hair growth on your upper lip, chin, lower abdomen or inner thighs?	☐ [1=Yes; 2=No]	Self-report by woman only
**28.4**	(a) Have you ever taken the oral contraceptive pill?	☐ [1=Yes; 2=No]	
	(b) *If yes,* Which type of pill did you take	☐ 1=Combined pill<br>2=Progestogen only (mini pill)<br>3=Don't know	If (a) is no, leave blank
	(c) *If yes,* for how long did you take it?	☐☐ [Age in completed years]	If (a) is no, leave blank
**28.5**	(a) Have you ever been pregnant?	☐ [1=Yes; 2=No]	
	(b) *If yes,* at what age was your first pregnancy?	☐☐ [Age in completed years]	If (a) is no, leave blank
	(c) *If yes,* how many pregnancies have you had?	☐☐ [Total number, 00 if none]	If (a) is no, leave blank
	(d) *If yes,* how many live births have you had?	☐☐ [Total number, 00 if none]	If (a) is no, leave blank
	(e) *If yes,* how many miscarriages have you had?	☐☐ [Total number, 00 if none]	If (a) is no, leave blank
	(f) *If yes,* how many abortions have you had?	☐☐ [Total number, 00 if none]	If (a) is no, leave blank
		**[Check that c = d + e + f]**	**Add d, e, and f to ensure it equals c**
**28.6**	Have you ever tried to become pregnant during a period of one year or more without success?	☐ [1=Yes; 2=No]	
**28.7**	(a) Are you pregnant at the moment?	☐ [1=Yes; 2=No]	
	(b) *If yes*, which trimester of pregnancy are you in?	☐ [1, 2 or 3]	1=1-3 months<br>2=4-6 months<br>3=7-9 months
**3.2 b**	Time of Questionnaire completion	☐☐:☐☐<br>[Hours:minutes; 24-hour clock]	Must be entered; use 24 hour clock

Body Composition Measurements using TANITA (pictures to be referred from manual Appendix for each step)

Body Composition/Bio – Impedance

Step1: Turn on the Power: Press the ON/OFF key

Step 2: Enter Clothes Weight: Enter the approximate weight of clothes worn by the participant using the numerical keys

Step 3: Select the Body Type: Select the body type from Standard Male, Standard Female, Athletic Male and Athletic Female

Step 4 : Enter Age:

Step 5 : Enter Height

Step 6 : "STEP ON" while flashing arrow will appear next to "STEP ON"

Start Measurement: Ask the participant to step on the Weighing Platform with bare feet in a stable position without bending the knees so they touch the electrodes

Taking Measurement: Make sure heels are placed on the posterior electrodes, and the front parts of the feet are in contact with the anterior electrodes

Measure the Impedance: When the grips are grasped with both hands, "0000" will appear at the bottom of the display and the impedance measurement will begin. The "0000" marks will disappear one by one during the measurement; after five full cycles, the measurement will be complete.

The participant should not step off the Weighting Platform until the "0000" symbols disappear completely.

Measurement is completed:

Once the body weight and impedance measurements have been completed, the overall body fat percentage will be shown at the bottom of the display and a buzzer will sound.

The printer should be ON such that the measurement results are printed out.

When You Continue to Measure: After printing is completed, go back to step 3. Follow the same procedure mentioned above.

Finish Measurement: Press the "ON / OFF key and turn off the power.

The output record for each subject will be entered immediately in the appropriate section of the questionnaire and the record also attached to the questionnaire booklet for future reference if required.

What you SHOULD DO

- Ask the participant to remove the heavy outer clothing like coat, shawl, pull-over etc.
- Ask the participant to hold both arms straight down while taking measurements

- Ask the participant to urinate before taking measurements to get a more accurate measurement
- Ensure that the participant's arms are not touching the side of the body
- Ask the participant to hold the grips only after the body weight figure on the display has stabilized
- The inner thighs are not touching each other during measurements
- Make sure the soles of feet are free of excess dirt
- Place the equipment on a level ground – look at the air bubble on the base of the equipment to check
- whether it is placed properly

What you SHOULD NOT DO:

- Avoid taking measurement of participants after vigorous exercise until sufficiently rested
- Do not measure on a surface that is strongly vibrating
- Do not take measurements while using transmitters, such as mobile phones, which may affect readings
- Do not take the measurement on participants who have an implanted pacemaker .

TANITA MEASURES

**37. 1 Height** □□□ **cm**	**37.2 Weight** □□□ **.** □ **kg**
**37.3 BMI** □□.□	**37.4 BMR** □□□□ kJ<br>□□□□ **Kcal**

**37.5 Total Body Fat**	**37.6 Impedance**
i. Fat Percentage □□.□□ %	i. Whole body □□□ Ω
ii. Fat mass □□.□□ Kg	ii. Right leg □□□ Ω
iii. Fat free mass □□.□□ Kg	iii. Left leg □□□ Ω
iv. Total body water □□.□□ Kg	iv. Right arm □□□ Ω
	v. Left arm □□□ Ω

Segmental Analysis

**37. 7 Right Leg**	**37.8 Left Leg**
i. Fat Percentage □□.□□ %	i. Fat Percentage □□.□□ %
ii. Fat mass - □□.□□ Kg	ii. Fat mass - □□.□□ Kg
iii. Fat free mass □□.□□ Kg	iii. Fat free mass □□.□□ Kg
iv. Pred.muscle mass □□.□□ Kg	iv. Pred.muscle mass □□.□□ Kg
**37.9 Right Arm**	**37.10 Left Arm**
i. Fat Percentage □□.□□ %	i. Fat Percentage □□.□□ %
ii. Fat mass - □□.□□ Kg	ii. Fat mass - □□.□□ Kg
iii. Fat free mass □□.□□ Kg	iii. Fat free mass □□.□□ Kg
iv. Pred.muscle mass □□.□□ Kg	iv. Pred.muscle mass □□.□□ Kg
**37.11 Trunk**	
i. Fat Percentage □□.□□ %	iii. Fat free mass □□.□□ Kg
ii. Fat mass - □□.□□ Kg	iv. Pred.muscle mass □□.□□ Kg

NIN Component of study

All parents and children will be invited to participate in the NIN component of the study. Consenting subjects will be brought to NIN for undergoing DEXA and vascular measurements. A subset of parents and siblings will also undergo the pulmonary function tests as a small validation exercise for the measurements obtained in the field using Card-Guard Spiro-Pro. One SRF to conduct carotid IMT scan and help in DEXA scan and two FW's to conduct the vicorder component and two clinic helpers will be stationed at NIN.

Sampling Frame

The APCAPS Phase I study will cover 20 villages, 10 in Intervention and 10 in the Control arm. The sampling list will be the list of 1826 households that have been followed in the Hyderabad DEXA study.
The villages in each arm will be paired by number of households and a random selection of 10 villages with their closest pair in size will be chosen for the study. This will result in about 700 households in each arm. i.e about 1400 households for the total study.

January 2011

From January 2011 onwards, there will be two teams working independently in the field. A training workshop will be conducted in early January to ensure standardised methods of measurement and complete understanding of the entire protocol so that both teams work in an identical manner to reach the requisite target recruitment by June/July 2011.

Each team will consist of one Medical Officer (JRF), 6 research Assistants, one laboratory technician, and one clinic helper. The Project Manager and Field Manager will ensure overall coordination and functioning of fieldwork in the two arms. One laboratory technician will be appointed at NIN and will work for the latter part of the day and analyse all samples for haemoglobin and blood glucose at the NIN lab on the same day as collection. In the event of absenteeism of one or the other Medical Officer/technician, the Project manager and Field manager will ensure alternate arrangements so that field clinics are not disrupted.

DXA Measurements

	DXA Scan	
**29.1**	DXA machine	☐ [1=New; 2=Old]
**29.2**	Researcher initials	☐☐☐
**29.3**	Whole scan taken	☐ [1=Yes; 2=No]
**29.4**	Spine scan taken	☐ [1=Yes; 2=No]
**29.5**	Hip scan taken	☐ [1=Yes; 2=No]
**29.6**	If not, specify reason	_____ _____
**29.7**	First L1-L4 measure taken	☐ [1=Yes; 2=No]
**29.8**	Second L1-L4 measure taken	☐ [1=Yes; 2=No]
**29.9**	First L2-L4 measure taken	☐ [1=Yes; 2=No]
**29.10**	Second L2-L4 measure taken	☐ [1=Yes; 2=No]
**29.11**	If not, specify reason	_____

DXA protocol

DXA machine: Hologic Discovery A
Phantom: Hologic spine phantom 14855

1. The DXA machine should be calibrated at the start of each day using the spine phantom.
2. If the participant is a woman, please check that the "DXA needed" box has been filled with a "1". If there is a "2" DO NOT PERFORM THE DXA SCAN.
3. If the woman is below 35 ask her if she is pregnant. If the answer is yes, DO NOT PERFORM THE DXA SCAN.
4. Ask the participant to take any mobile phones off their person and remove any metallic items eg jewellery, watches, clothing with metal buttons
5. Ask the participant to change into light clothing. Pyjama trousers are provided for men. For women, full-length gowns are available if necessary but the scan can generally be performed with sari or salwar kameez.
6. In the Hologic programme, click on the PERFORM EXAM icon.
7. Click on NEW PATIENT.
8. Enter Last Name, First Name, subject ID number (from their questionnaire ID) Sex, DOB and name of study.
9. Ask the participant to lie supine on their back on the bed of the DXA machine, so that their left hand side is on the outer edge of the DXA scanner. Ask them to bend their feet inwards so that their big toes are touching. Arms should be by their sides with palms flat on the bed. Check the participant is comfortable and then ask them to remain still until the scan is complete.

10. Click on NEW SCAN.
11. Select TOTAL BODY from the drop down menu.
12. Click on START scan.
13. Once the total body scan is complete, ask the participant to lift their legs up and rest their calves on the purple support box with their knees bent so that their spine is flat on the bed. Check the participant is comfortable and then ask them to remain still until the scan is complete
14. Click on NEW scan.
15. For scan type, select LUMBAR SPINE from the drop down menu.
16. Under type of scan select FAST ARRAY option and untick the "use default settings" box.
17. Using the controls on the side of the DXA scanner, ask the scanner to show a red laser cross over the area it is about to scan. The participant's position can be adjusted using the controls on the DXA scanner to move the bed so that the cross is over the lumbar spine area
18. Click on START SCAN.
19. It may be necessary to reposition the scan if the image is not in the correct location. To do this click on REPOSITION SCAN. It is then possible to drag the image into the required position. To resume scanning, click on RESTART SCAN.
20. Once the scan is complete, remove the purple box from under the participant's legs. Ask the participant to rest their left foot against the triangular support and strap it in place using the velcro strap. Check the participant is comfortable and then ask them to remain still until the scan is complete.
21. Click on NEW SCAN.
22. For scan type, select LEFT HIP from the drop down menu. Make sure the "use default settings" box is ticked.
23. Using the controls on the side of the DXA scanner, get the scanner to show the red laser cross over the area it is about to scan. The participant's position can be adjusted so that the cross is over the left hip area using the controls on the DXA scanner.
24. Click on START SCAN.
25. It may be necessary to reposition the scan if the image is not in the correct location. To do this click on REPOSITION SCAN. It is then possible to drag the image into the required position. To resume scanning, click on RESTART SCAN.
26. Complete the first part of section E of questionnaire, indicating whether each DXA scan has been completed.
27. Enter a "1" into the DXA box on front of questionnaire.
28. Once all the participants for the day have been scanned, print out the results of the total body, lumbar spine and hip scans for each participant. These will be collected by the data entry officer each day.
29. On Monday, Wednesday and Friday afternoons, carry out the L1-L4 and L2-L4 calculations twice for each participant. Print the forms for each participant and staple together. These forms will be collected and entered into the database by the Data Entry Operator.
30. At the end of each week, save all the study files for that week to CD. This will be collected by the Data Entry Operator.

Coronary Measures and Medical History

	**Medical history**.(to be completed by the Medical Officer in the field)		
**30.1**	(a) Have you been diagnosed with any of the following conditions?		(b) *If yes*, age when diagnosed
**30.2**	High blood pressure	(a) ☐ [1=Yes; 2=No]	(b) ☐☐ [Age in completed years]
	(c) Are you on regular medication for your high blood pressure?		☐ [1=Yes; 2=No]
	(d) Name of medicine:	_____	
	(e) Who diagnosed condition	☐ [1=allopathic doctor; 2=homeopath; 3=ayurvedic doctor 4=RMP – registered medical practitioner; 5=Other]	
**30.3**	Diabetes (high blood sugar)	(a) ☐ [1=Yes; 2=No]	(b) ☐☐ [Age in completed years]
	(c) Are you on a regular diet for your diabetes?		☐ [1=Yes; 2=No]
	(d) Are you on regular tablets for your diabetes?		☐ [1=Yes; 2=No]
	(e) Name of medicine:	_____	
	(f) Are you on a regular treatment with insulin?		☐ [1=Yes; 2=No]
	(g) Do you attend a hospital or GP diabetic clinic?		☐ [1=Yes; 2=No]
	(h) Who diagnosed condition	☐ [1=allopathic doctor; 2=homeopath; 3=ayurvedic doctor 4=RMP – registered medical practitioner; 5=Other]	
**30.4**	Heart disease	(a) ☐ [1=Yes; 2=No]	(b) ☐☐ [Age in completed years]
	(c) Are you on regular medication for your heart disease?		☐ [1=Yes; 2=No]
	(d) Name of medicine:	_____	
	(e) Who diagnosed condition	☐ [1=allopathic doctor; 2=homeopath; 3= ayurvedic doctor 4=RMP – registered medical practitioner; 5=Other]	
	(f) Type of heart disease	☐ [1=angina; 2=heart attack; 3=heart failure 4=don't know; 5=Other]	
**30.5**	Stroke (paralytic attack)	(a) ☐ [1=Yes; 2=No]	(b) ☐☐ [Age in completed years]
	(c) Who diagnosed condition	☐ [1=allopathic doctor; 2=homeopath; 3=ayurvedic doctor 4=RMP – registered medical practitioner; 5=Other]	
**30.6**	Asthma, asthmatic bronchitis or allergic bronchitis?	(a) ☐ [1=Yes; 2=No]	(b) ☐☐ [Age in completed years]
	(c) Have you had an attack of asthma in the last year?		☐[1=Yes; 2=No]
	(d) *If you have asthma,* are you on regular medication for asthma? (tablets/inhaler)		☐[1=Yes; 2=No]
	(e) Name of medicine:		
	(f) Who diagnosed condition	☐ [1=allopathic doctor; 2=homeopath; 3= ayurvedic doctor 4=RMP – registered medical practitioner; 5=Other]	
**30.7**	Thyroid problem	(a) ☐ [1=Yes; 2=No]	(b) ☐☐ [Age in completed years]
	(c) Are you on regular medication for your thyroid problem?		☐ [1=Yes; 2=No]
	(d) Name of medicine:		
**30.8**	Tuberculosis	(a) ☐ [1=Yes; 2=No]	(b) ☐☐ [Age in completed years]

	(c) Are you on regular medication for your thyroid problem?			☐ [1=Yes; 2=No]
	Name of medicine:			
**30.9**	Depression	(a) ☐ [1=Yes; 2=No]	(b) ☐☐ [Age in completed years]	
	(c) Are you on regular medication for your depression?			☐ [1=Yes; 2=No]
	Name of medicine:			
**30.10**	Peptic ulcer	(a) ☐ [1=Yes; 2=No]	(b) ☐☐ [Age in completed years]	
**30.11**	COPD	(a) ☐ [1=Yes; 2=No]	(b) ☐☐ [Age in completed years]	
**30.12**	Emphysema	(a) ☐ [1=Yes; 2=No]	(b) ☐☐ [Age in completed years]	
**30.13**	Chronic bronchitis	(a) ☐ [1=Yes; 2=No]	(b) ☐☐ [Age in completed years]	
**30.14**	Cancer	(a) ☐ [1=Yes; 2=No]	(b) ☐☐ [Age in completed years]	
	(c) *If yes,* what type of cancer:			

**MEDICAL EXAMINATION**				
	**Carotid IMT**	(a) Far wall	(b) Near wall	
**31.1**	Right common carotid artery image taken	☐ [1=Yes; 2=No]	☐ [1=Yes; 2=No]	
**31.2**	Any problems taking images	☐ [1=Yes; 2=No]	☐ [1=Yes; 2=No]	
**31.3**	If yes, specify reason	_____	_____	
	**Pulse Wave Velocity**			
**32.1**	Room temperature	☐☐ . ☐ [degree Celsius]		
**32.1**	Have you had a meal in last 2 hours?	☐ [1=Yes; 2=No]		
**32.2**	Proximal distance (carotid to notch)	☐☐ . ☐ [cm]		
**32.3**	Distal distance (notch to upper thigh)	☐☐ . ☐ [cm]		
		**(a) First measure**	**(b) Second measure**	**(c) Third measure**
**32.4**	Systolic BP (supine)	☐☐☐[mmHg]	☐☐☐ [mmHg]	☐☐☐ [mmHg]
**32.5**	Diastolic BP (supine)	☐☐☐[mmHg]	☐☐☐ [mmHg]	☐☐☐ [mmHg]
**32.6**	Pulse rate (supine)	☐☐☐ [bpm]	☐☐☐ [bpm]	☐☐☐ [bpm]
**32.7**	Pulse wave velocity	☐☐.☐ [m/s]	☐☐.☐ [m/s]	☐☐.☐ [m/s]
**32.8**	Transit time	☐☐☐ [ms]	☐☐☐ [ms]	☐☐☐ [ms]
**33.6**	Any problems taking readings	☐ [1=Yes; 2=No]	☐ [1=Yes; 2=No]	☐ [1=Yes; 2=No]
**33.7**	If yes, specify reason			

Carotid measures

- Please make sure that no drinks are placed on the table with the carotid IMT, Arterial stiffness or PWV equipment
- Please ensure that there are no mobile phones switched on near the equipment when taking the measures. Please ask the subject to remove any phones from their person.

Protocol for Carotid IMT

1) Connect ultrasound box to Dell laptop using the white USB cable (not bifurcated cable).
2) Turn ultrasound machine on.
3) Ask the participant to lie down on the bed. Explain what you are going to do. Say that if it is causing any discomfort they should let you know.
4) Click on the Ethiroli icon on the desktop to open the ultrasound programme.
5) Enter the subject details by going into the open folder from the file icon seen on left top of the screen and then press NEW to start the new scan. This can be identified by looking at the name of the subject seen on the left hand side of the screen.
6) Put gel on probe.
7) Place probe on participant with marked side pointing downwards and unmarked side pointing upwards.
8) Working only on right side, scan the whole carotid area, first transversally to view the trachea, thyroid, jugular vein and carotid artery. Then turn probe to view carotid artery only.
9) Place probe to get clear image of the near wall of the distal common carotid artery (10 mm). Make sure that the white arrow on the left hand side of the screen (focal point) is at the same level as the wall you are taking the image of. The focal point can be adjusted using the keys with the up and down arrows on the ultrasound keypad. Make sure that the carotid artery appears horizontally on the screen.
10) When you have a clear view of the intima, press space bar on the laptop to freeze image.
11) To save the image press F5 on the key board of the laptop and a window will appear allowing you to choose the site that you have captured the image of(far wall or near wall or both). Tick the appropriate box. A label should then appear on the top right of the image with the study ID, name and site of the picture and the icon at the left bottom of the screen where far wall or near wall turns to white from red colour.
12) Repeat steps 5 to 13 and continue the same for the rest of the subjects for that day.
13) At the end of the day, open the APCAPS imt folder located in C:\Documents and Settings\DOCTOR\My Documents\. Open the ethiroli folder located on desktop as a shortcut to see the images folder. Copy the images folder and paste to APCAPS imt folder\ [year folder}\[month folder]\ and rename it with date (eg jan8).
14) Go to the ultrasound software and open the windows where the subject's names are entered. Click the CLEAR IMAGES icon. This opens a window asking a password, enter 1234 and press OK to clear the images in the images folder as well as on the subject details screen for that day.

NOTE: if it is possible to capture both near and far walls of the common carotid in the same image, save the same image twice, naming it first as a near wall image and second as a far wall image.

15) Give participant tissue to wipe off gel.

Storage

16) At end of day, wipe any gel off the probe and store in bubble wrap.
17) Every Friday, the images for the week should be saved on to a CD.

Problem spotting

- If you do not have a clear image, try putting on more gel.
- Do not press too hard (e.g. if participant coughs or complains).
- Make sure not to take videos by accident as these cannot be processed
- Do not clean probe with spirits or other agents. Gently wipe with soft cotton.
- Make sure probe is dry when it is stored.
- If the ultrasound machine loses power and you cannot get the laptop to recognise it, unconnect all the cables and restart the laptop. Reconnect the cables- you may need to use a different USB port on the laptop for the connection.
- If machine does not work call Haneef on 094-4000-2622 / Ravi Ganeshan 09944934354

Abbreviations for naming the files

nw: near wall

fw: far wall

cca: common carotid artery

To Save:

At the end of the day, go into the patient file and click on ARCHIVE. A "Save as" box will appear. Browse to find the *APCAPS IMT* file and save as a zip file. Name the file with the date eg 08 Jan 2011

Protocol for Pulse Wave Velocity

1. Connect vicorder USB cable to Wipro laptop.
    a. Bifurcation ends into 2 USB ports of the laptop
    b. Other end should be connected to the circular port (8 way socket) at the rear of the vicorder box with the red dots lined up
2. Connect the red and blue leads to the press 1 and press 2 ports on the vicorder box. The red lead goes into the red port and the blue lead into the blue port.
3. Switch on laptop. The blue light should light up on the Vicorder.
4. Double click Vicorder icon on desktop.
5. Get the subject to lie down in the supine position. Explain to the subject about the two cuffs (neck and thigh). Check that the subject is comfortable with the cuffs.
6. Place 10cm blue cuff as high as possible on the thigh, making sure it is not too tight or too loose. The tube on the thigh cuff should be pointing towards the head and should be in line with the femoral artery.

7. Feel the carotid pulse and place the 2.5cm cuff (white) attached to the black Velcro neck band on to the neck (right carotid), making sure it is not too tight and not too loose (you should be able to fit 1-2 fingers inside the band). The white pressure pad should be on the inside of the cuff with one end starting in the middle of the neck and the other (end with the tube) over the carotid area. Make sure that the tube is pointing downwards.
8. Connect the red lead to the proximal (neck) cuff and the blue lead to the distal (thigh) cuff.
9. Ensure that the subject has been resting in the supine position for TEN MINUTES before the PWV reading
10. Take the subject's blood pressure 3 times and record the values on the questionnaire.
11. Measure path lengths in cm with tape measure and enter into questionnaire.
    a. Carotid to suprasternal notch
    b. Suprasternal notch to thigh (upper part of cuff)
12. Click PWV icon on the quick launch tab on the right hand side of the screen. This will bring up the study folder.
13. Enter subject details in study folder
    a. Study Name (APCAPS), Patient ID, First name, Last name, Sex, DOB (to change DOB you need to highlight each section of the date and use the up and down arrow keys)
14. For the proximal site, select **carotid** from the drop down menu. For the distal site, select **right femoral** from the drop down menu.
15. Enter the following into the study folder:
    a. Distance from notch to thigh
    b. Systolic blood pressure (use the third reading)
    c. Diastolic blood pressure (use the third reading)
16. Explain to the subject that you are about to inflate the cuffs and that they will feel some pressure on their neck and thigh. Ask them to tell you if they feel any discomfort.
17. Press SPACEBAR to inflate the cuffs
18. Once inflated, acquire steady pulse (there should be 2 screens of even waveforms for both carotid and thigh). Capture data by pressing SPACEBAR.
19. Wait for a couple of seconds for the PWV and TT values to stabilize. Record PWV and TT values on questionnaire.
20. To save data
    a. Press ENTER.
    b. Browse to find the *PWV actual results* spreadsheet in the Real study PWV folder. Select this and Click OK. A message will appear asking if you want to overwrite this file, click Yes.
21. Press SPACEBAR for second reading. Repeat steps 17-20.

NOTE: For the second and third readings for a subject, the readings will automatically be saved to the same spreadsheet when you press ENTER.

22. Repeat for third reading
23. Press ESCAPE to close the PWV screen when you have completed the 3 readings.

Quality Control

To check the quality of the waveforms, for one subject per day, the images of the PWV waveforms should be recorded. To do this:

1. After saving a recording, press F4 to export the data. Browse to the *qc PWV* folder in the *real study PWV* folder.
2. Save the image as a jpeg and name it with the patient ID, name and number of the recording eg 1,2,3

Problem spotting

- If the carotid waves are not uniform, make sure that the vicorder wires are not twisted, that the neck cuff is not too loose and that the subject's head and shoulders are raised at an angle of 30°.

<u>Pulse Wave Analysis</u>

1. See steps 1-4 in the PWV protocol for setting up the Vicorder equipment.
2. Ask the subject to lie down in the supine position.
3. Take the subject's blood pressure in their right arm three times.
4. Explain to the subject about the two Vicorder cuffs (brachial and thigh).Check that the subject is comfortable with the cuffs.
5. Place a 10cm blue cuff as high as possible on the thigh, making sure it is not too tight or too loose. The tube on the thigh cuff should be pointing towards the head and should be in line with the femoral artery. Attach the tube of this cuff to the blue Vicorder lead.
6. Place a 10cm blue cuff around the upper arm with the tube pointing downwards. Attach the tube of this cuff to the red Vicorder lead.
7. Measure the distance from the top of the brachial cuff to the top of the femoral cuff with a tape measure.
8. In the quick launch tab on the right hand side of the Vicorder programme, select PWA. This will bring up the study folder.
9. Enter the subject details in the study folder: Study Name (APCAPS), Patient ID, First name, Last name, Sex, DOB (to change DOB you need to highlight each section of the date and use the up and down arrow keys)
10. For the proximal site, select **right brachial** from the drop down menu. For the distal site, select **right femoral** from the drop down menu.
11. Enter the following into the study folder:
    a. Aortic path length (distance between cuffs)
    b. Systolic blood pressure (use the third reading)
    c. Diastolic blood pressure (use the third reading)
12. Click on OK. This will bring up the data capture screen.
13. Press F3. The screen should now split in half, with the top half for the brachial readings and the bottom for the thigh readings.
14. Inform the subject that you are about to inflate the cuffs and that they will feel some pressure on the thigh and upper arm.
15. Press the SPACEBAR to inflate the cuffs.
16. Wait until there have been several successive screens of uniform waves in both windows and the values in the grey boxes have stabilised.

17. Press the SPACEBAR to capture the data.
18. To save the data:
    a) Press ENTER.
    b) Browse to find the *Brachial PWA* spreadsheet in the Real study PWV folder. Select this and Click OK. A message will appear asking if you want to overwrite this file, click Yes.
19. Repeat steps 14-17 twice more so that you capture 3 readings for each subject.

NOTE: For the second and third readings for a subject, the readings will automatically be saved to the same spreadsheet when you press ENTER.

<u>Quality Control</u>

To check the quality of the waveforms, for one subject per day, the images of the PWV waveforms should be recorded. To do this:

1. After saving a recording, press F4 to export the data. Browse to the *qc Brachial PWA* folder in the *real study PWV* folder.
2. Save the image as a jpeg and name it with the patient ID, name and number of the recording eg 1,2,3

VICORDER INSTRUCTIONS

Plug in VICORDER to laptop with main USB / 8-way cable.

Load up software onto the laptop once prompted to do so.

On the main screen, click on the START menu in the bottom left hand corner.

Then go to SETTINGS.

Then go to CONTROL PANEL.

In Control Panel, switch to CATEGORY VIEW if you are in Classic View. Once in Category view, select PERFORMANCE & MAINTENANCE.

In Performance and Maintenance, select SYSTEM at the bottom of the window and then select HARDWARE, then select DEVICE MANAGER.

In Device Manager, scroll down the list until you find the USB driver name with an exclamation mark next to it. If you cannot see this, then double click on the USB name (Universal Serial Bus Controllers).

Previously when this software has been loaded problems arose with the ***USB <-> Serial***

Right click on the USB name which has the exclamation mark next to it and select UPDATE DRIVER.

This opens the HARDWARE UPDATE WIZARD.
Within the Wizard window, select the option to INSTALL FROM A LIST OR SPECIFIC LOCATION, then click NEXT.

Then select the option to SEARCH FOR BEST DRIVER IN THESE LOCATIONS.

Tick the box to INCLUDE THIS LOCATION IN THE SEARCH (make sure the other option is unticked).

Select BROWSE and select C drive, Programme Files, Skidmore Medical, Vicorder, Driver. If Driver is not one of the options listed in Vicorder, double click on DATA and DRIVER should be available from this list.

Then click NEXT

Then click FINISH

The light on the VICORDER should now come on and remain on.

Now attach the leads to the front of the Vicorder box and the audio lead at the back and open the Vicorder programme via the icon on the desktop.

Once the Vicorder programme is open, select FILE, FIX DATABASE. A message should appear saying there is no error to fix. Select OK and then the programme will automatically check the license.

Data back-up of coronary data

Every Friday, all of the data from the previous week needs to be backed up onto a CD which must be clearly labelled with the study name and end date for that week. Eg.

- APCAPS 23rd January 2011
- APCAPS 30th January 2011

Files for the two separate medical measurements need to be copied onto the CD.

1) Vicorder – Protocol for Saving Data
Data will be stored each day for each individual subjects in two main excel spreadsheets on the Wipro laptop:
C:\Documents and settings\DOCTOR\My Documents\APCPAS PWV

Spreadsheet names:
PWV actual results Pulse Wave Velocity recordings
Brachial-PWA Brachial Pulse Wave Analysis recordings

At the end of each week the data stored within the two spreadsheets must be tidied up to remove excess information that is not required.

1. Open the file *PWV actual results* file in excel.
2. Scroll through the data and delete each row that has titles but no values e.g. Study Name, ID No, Last Name etc. This will be every fourth line or so and any empty rows in between.
3. The database should only contain the output for each individual subject and not the title headings for their data. So, rows starting with 'real', 'ID=' etc all need to be kept.
4. Repeat steps 2, 3 and 4 for the *Brachial-PWA* file.

Once the databases have been tidied up each week, they both need to be saved to the CD. In addition there are 2 folders in the "real study PWV" folder with the quality control images stored as jpegs. These should also be saved to the same CD.

2) Carotid IMT- Protocol for saving data
Images for Carotid IMT will be saved as jpegs each day in date labelled folders on the Dell laptop in:
C:\Documents and settings\DOCTOR\My Documents\APCAPS IMT

1. **In the APCAPS IMT folder, create a new folder for the week that has just been completed and name it with the date eg 17 – 21 Jan 2011.**
2. **Drag all of the day folders for this week into the new folder.**
3. **Save the week folder on to the CD.**
4. **Compress the weekly folder into a zip file. To do this, right click on the folder, go to SEND TO, then COMPRESSED (ZIPPED) FOLDER.**

Departure from clinic – completed by Field Manager / Project Manager

Reimbursement of participants

Distribution of reimbursement to participants is the responsibility of the Field Manager / Project Manager, and is undertaken at the end of the day when all examinations have been completed.

The level of reimbursement was arrived at using the following principles. Understanding of these principles may be useful at times for clarification, or in unusual circumstances where an appropriate level needs to be calculated. However, these do not need to be routinely explained to the participant, and reimbursement should be offered, as far as possible, on a lump sum basis using the suggested scales. In case there is doubt about the correct scale, please err on the higher side remembering that participants are volunteers, under no obligation to participate.

The APCAPS subjects are reimbursed for their time and loss of wages while attending the day clinic at the village and for attending the clinics at NIN for DEXA and vascular studies.

Attendance at Village site clinic: Rs. 200 per subject.
Attendance at NIN for DEXA and vascular studies: Rs. 200 per subject.

Proforma for APCAPS Reimbursement :

APCAPS - HYDERABAD - REIMBURSEMENT OF EXPENSES

Date: Name of the village:

Voucher No: APCAPS_

No. Of Participants:

SL NO	Subject ID	Name of participant	Amount paid	Signature/ Thumb impression

Total amount reimbursed

Refreshments

Total ------>

Prepared By Verified By Approved By

Proof of identity :

It is important that some proof of identity is available for the subject that confirms the relationship and place of residence of the subject. This requirement will be explained in the invitation letter. Examples of proof of identity include the ration card, voting card, work identity card, driving or other license, or letter from the village head. The subjects will be requested to bring a photocopy along with the original. The photocopy should be checked against the original and retained for record. If the subject brings the original but forgets the photocopy, the study team should get a photocopy made locally (the money for this can be claimed back). Where the subject forgets to bring a proof along, they should still be recruited, but only after asking suitable questions to confirm the identity. This will have to be done in a very careful manner to avoid upsetting the participants. In such cases the participants should be asked to post a photocopy of the proof after their return home, and they should be chased up for this if necessary. Where no proof becomes available, this fact should be recorded in the database.

	**Summary sheet (to be completed at time of reimbursement)**			**Instructions**
	**Component completed**			This section should be completed at the end just before the subject departs, generally at the time of reimbursement
	**Reimbursement**			
**1.1**	Reimbursement given	☐ [1=Yes; 2=No]		Subject's signatures should be taken on receipt
**1.2**	Identity proof taken	☐ [1=Yes; 2=No]		Proof should be copied & filed away
	Subject recall			
**1.3**	Subject needs to be recalled	☐ [1=Yes; 2=No]		Recall may be needed for repeatability, validation or incomplete study
**1.4**	Recall for repeatability study	☐ [1=Yes; 2=No]		Repeatability studies will be done on random 5% sample
**1.5**	Recall for validation study	☐ [1=Yes; 2=No]		Validation studies will be done on random 5% sample
**1.6**	Recall for incomplete study	☐ [1=Yes; 2=No]		Subject may need to be recalled if some of the information was not completed
**1.7**	If yes, is the subject willing to return?		☐ [1=Yes; 2=No; 3=Undecided]	
**1.8**	If undecided, date status will be reviewed:		__ __/__ __/__ __ [DD/MM/YY]	Negotiate the date with participant
**1.9**	**If recalled, clinic visit details**			
	(a) Date of clinic visit [DD/MM/YY]	(b) Travel [1=Self; 2=Team]	(c) Outcome [1=Yes; 2=No]	Start and end dates of period relate to time over which the relative is expected to arrive at the clinic e.g. if the person is expected anytime in the month of august, enter start and end dates for the month

	__ __/__ __/__ __			Travel relates to whether the subject has agreed to make his/her own travel/stay arrangements or if the team has to make part/all of the arrangements
	__ __/__ __/__ __			
	__ __/__ __/__ __			
**1.10**	**Summary sheet notes**			

MEDICAL REFERRAL

Who should be referred?

- Medical help can be obtained from the Medical Officer in the field team.
  - Complaining of possibly serious medical conditions
    - Chest pains on exertion, breathlessness, severe pain
  - Wanting a second opinion for known medical conditions
    - Discuss with SRF and negotiate with Referral hospital/Medical college .

Abnormal results and Referral:

All reports will be communicated to the subjects. For any abnormal results based on the clinical examination and laboratory results like anaemia, high blood pressure or sugar etc. the subject will be seen by medical officer and if required referred to the local medical college hospital/referral hospital.

First aid:

The doctor will provide first aid as needed or refer the subject to the local medical college hospital as needed.

In our scenario, we are most likely to encounter cases as:

Heat exhaustion:

If you suspect heat exhaustion, get the person out of the sun into a shady location. Lay the person down and elevate the feet slightly. Loosen or remove person's clothing. Cool the person by spraying him or her with cool water and fanning and have the person drink cool water. Monitor the person carefully. If person shows symptoms and signs of a heatstroke like temperature greater than 104 F, fainting, confusion or seizures, the person should be immediately referred.

Fainting

If a subject looks or feels faint during the procedure, it should be discontinued. The subject should be asked to place their head between their knees. They should subsequently be asked to lie down. If they are happy for the test to be continued after a suitable length of time, it should be done so with the subject supine and the circumstances should be recorded. They may wish to discontinue the procedure at this point, but willing to give the blood sample at a later time.

Needle stick injuries

The wound should be encouraged to bleed. The wound should be washed with soap and warm water, if available. Other hand cleaner may be used if water is not available.

Bruises

For any bruises following the blood sampling, elevate the injured area and apply ice or cold pack for 30 to 60 minutes at a time for a day or two after the injury.

INVENTORY AND CARE OF EQUIPMENT

All the equipment will have to be handled with care. It is the responsibility of the Field Manager to ensure at the end of the day when clinics are over; field workers who are carrying out the anthropometric measurements will pack the equipment properly and keep in a safe place. In case of any damage to the instruments or loss of instruments, this will have to be communicated to Delhi/Bristol or NIN immediately so that a replacement can be made.

An inventory of the general items as well as biochemistry items will have to be maintained by the centre and sent to Delhi/Bristol and NIN at the end of the month. Any item which needs to be procured will have to be informed well in advance.

ITEM	USED BY	TYPE AND NUMBER NEEDED IN STOCK
Batteries		
	Scales (6xAA)	40xAA
	BP machine (4xAA)	
	Spirometer (2xAA)	4x9V
**Anthropometry**		
Spirit	Anthropometry	2 bottles
Cotton wool	Anthropometry and doctor	4 packs
Calculator	Anthropometry	2 spare
Spirometry tubes	Anthropometry	500 per month used
**Doctor**		
Ultrasound gel	Doctor	2 bottles spare
**Stationery**		
Pens	All	50 pens
CDs	Doctor	30 spare
**Biochemistry**		
Needles disposal bin		
Glasses		
**Miscellaneous**		
Soap		
Toilet tissue		
Hand sanitizer		
Questionnaires		
Labels		

Communications-

- Every day the Project Manager will check the mail once in the morning and once at the end of the day. Mails which require immediate attention should be given high priority. All such mails must be replied by the evening of the day and even if the mentioned task cannot be done immediately, this should be communicated with the likely date of completion.

- Wherever deadlines have been specified, it is important that these are met. If for some unavoidable reasons it is not possible to complete a task in time, then this should be communicated well ahead of time so that a backup plan can be thought of.

- The must have a date diary-a large size one with a page to date. This is to make note on that day of the diary of the things to be done. Once any action is completed, it can be cancelled off from the list. With the diary you can also plan ahead for actions.

- Matters related to biochemistry, database and finances should be addressed to the concerned person and copied to the Project Manager.

- All urgent / important mails need to be acknowledged.

- Anything sent through post (photocopy of questionnaires, bills, attendance etc.) should be preceded by a mail to keep the other centre informed. Again receipt of anything should also be acknowledged by mail.

CALIBRATION SYSTEM

Daily Calibration

- All instruments which are being used for measurements in the clinics have to be calibrated on all the days when clinics are held.
- As far as possible, readings for the calibration to be taken on the same 2 fieldworkers everyday.
- Calibration has to be done early morning on arrival at the clinics before the subjects come.
- This has to be entered into a calibration sheet provided and then saved into the PC at the end of the day.
- Calibration needs to be done on active instruments only i.e., the one being currently used. In cases where both instrument is being used at the same time, then calibrations to be done for both.
- For the calibration of Card Guard Spiro Pro readings are recording on two fieldworkers and the readings are saved in an excel file.
- These readings need to be reviewed at the weekly meetings and if there is a very wide variation in the readings, then this needs to be communicated to the coordinating centre for appropriate action.
- For calibration of the callipers, measurements of a thin and thick book will suffice.

Monthly Calibration

Besides daily calibration, monthly calibration of the instruments needs to be done. The procedure for this is as below:

Weighing Machine:

- This can be done by comparing against standard weights starting from 5kg and above.

Stadiometer:

- The metallic tape can be used for this and readings to be taken at 75 and 150 cms on the stadiometer.

Callipers:

- Same as the daily calibrations, i.e., using 2 books with different thickness.

BP apparatus:

- Comparing it against a sphygmomanometer. It is important however to remember that the same sphygmomanometer to be used throughout the study.

Spirometer:

- The Card-Guard SpiroPro should also be calibrated once a month using a standard three litre calibration syringe. Method - . With the Spirometer in zero position, and using an appropriate mouthpiece or connector, the three litre calibrating syringe will be emptied into the spirometer. This procedure will be repeated three times, with the spirometer returning to zero position after each syringe check. The spirometer should read within +/- three percent of the syringe volume. After the calibration check, the research staff will perform upto eight maneuvers on himself/herself. The same person will do the calibration each time. The graphs are saved and readings entered into an excel sheet and reviewed by the coordinating center every month. Any marked variations will mean that the device needs to be checked and appropriate action taken.

DAILY CALIBRATION SHEET

Date	Wt. of FW no. (weighing machine no.___)	Ht of FW no. (stadiometer no_____)	measurement of thin book(calliper no____)	measurement of thick book(calliper no____)	BP of FW no. (Omron No____)	PFT values FW no.	Comments

MONTHLY CALIBRATION

Date	Standard weights(5,10,15kgs) against digital weighing scale (no.__)	Ht of FW(stadiometer no_____) at 75 and 150 cms	Measurement of thin book(calliper no____)	measurement of thick book(calliper no____)	BP of FW no. using Omron No____)	BP of FW no. Using sphygmomanometer	PFT values using 6 L syringe	PFT values for FW no.	Comments

FAQs

What if a woman (or in some cases men e.g. if elderly) refuses to travel alone?

- In such cases additional payment (50% extra) may be made for an accompanying person. However, the accompanying person is not to be included in the study. If the accompanying person wishes to have him or herself checked, a short examination (or blood pressure measurement) may be considered to ensure cooperation of the family; however, the data will not be recorded.

What if the subject arrives to the clinic but is not completely fasting?

- As long as the subject has not eaten or drank anything other than water in the last 4 hours, the sample should be collected and the subject recruited.

What if a woman is pregnant or may be pregnant?

- If there is any possibility that a woman may be pregnant, ask her to take a pregnancy test. Women who are pregnant undergo all the questionnaires and tests in the same way but do **not** undergo screening through DXA.
- Women who are pregnant are not asked to fast before the examination clinic.

Reporting System

This will be a regular reporting system from NIN to Delhi/Bristol.

NIN to Delhi / Bristol.

The Project Manager is required to send the weekly reports to Delhi/Bristol by Wednesday afternoons. Reports for a week will contain data through the last Wednesday to the current Tuesday. The weekly reports to be send to Delhi/Bristol are:

- Weekly summary report
- Fieldworker performance sheet
- Database copies

At the end of each month (on the last Wednesday of the month along with the weekly reports), Field Manger and Project Manager are required to note the following:

- inventory of the general items
- inventory of biochemistry items

* Each research assistants will also maintain the daily progress report which can be reviewed by the team leader every week at the weekly meeting on Friday.
** Every Friday there is a phone call between the Project Manager and PI at LSHTM so as to keep them abreast with the activities carried through the month and also discussing any issues/problems requiring solution.

IMPORTANT: All reports to be prepared by the Data Entry Operator and reviewed by the / Project Manager before sending it to Bristol.

At the end of each month the Project Manager is required to send the attendance signed by the Principal Investigator to Mr I Alex / Ms Lata by the 25th of each month.

The statement of Expenditure along with all supporting documents is required to be sent to Mr I Alex / Ms Lata biweekly.

Format for Statement of Expenditure:

REIMBURSEMENT OF EXPENSES

PERIOD 01-MAY-10 TO 15-MAY-10

STMT No. : CCDC/APCAPS/001 **STMT DATE :**

Sl No	DATE	INV / VCHER No. (IF ANY)	PARTICULARS	A/C HEAD	AMOUNT	REMARKS
01						
02						
03						
04						
05						
06						
07						
08						
09						
10						
11						
12						
13						
14						
15						
**TOTAL -----> **					**0**	

Expense Incurred	0
Less : Advance Recd (If Any)	
Net Receivable	0

Prepared By **Verified By** **Approved By**

DATA ENTRY

The database construction will be done in Bristol; set-up, operation and administration will be carried out at NIN by the Project Manager in consultation with Pete Shiarly at Bristol.

Two data entry operators will be involved in simultaneous data entry at NIN and data quality control procedures will be followed as outlined below. Copies of the database will be sent at regular intervals to the coordinating centre.

DATA QUALITY CONTROL PROTOCOL

Data quality of the questionnaires

Every week when the team meets, the Field Manager / Project Manager picks 2 questionnaires filled in the current week by one interviewer. The Field Manager / Project Manager needs to go through the questionnaire, question by question with the entire team, to identify incomplete/missing entries and any errors. The purpose of this is to rectify and clear any doubts which the interviewers may still have.

Data quality of the database

For the data quality of the database, the Filed Manager needs to compare the questionnaires with the same entered into the database. For this, 10% of the questionnaires per week, already entered into the database, could be checked. A blank questionnaire is kept at hand when doing this exercise and errors/missing data identified in the database can then be noted as tally lines against the particular question in the blank questionnaire. For e.g, if there is an error in Qs. 3.1 of the questionnaire, then a tally line needs to be marked against the question. This exercise has to be repeated every week so that by the end of the month, n questionnaires would have been covered. Then the tally lines in the blank questionnaire can be added up for all the n questionnaires and this adds up to the total number of errors and gives us the numerator for the error rate.

$$\text{Error rate} = \frac{\text{Total number of tally lines}}{\text{Total number of filled fields in the questionnaire x n}}$$

Filing System

1) ***All the questionnaires filled should be filed appropriately***.
2) For completed questionnaires pending for data entry, one separate box can be maintained. All questionnaires in this box should be filed according to ID. For easy management, boxes with ranges of IDs starting in a series.
3) Once the completed questionnaires have been checked by the Field Manager these will be filed by ID in the filing cabinet draw “For Data Entry”.
4) After data entry the questionnaires are filed by ID in the filing cabinet draw “Entered Questionnaires”
5) The photocopies of the ID will also be filed by ID in the filing cabinet draw “IDs”.

Clinics

For the clinics, 20 file folders will be maintained. On arrival of a subject to the clinic, a file folder containing the following will be handed over to him/her by the fieldworker who is receiving them. The file folder for the participant will contain the following:

- Participant Info sheet
- Consent Form
- Questionnaire
- Labels
- Reimbursement form
- Feedback form

This file folder will then be handed to the Field Manager at the end of the clinic procedures who will then check for missing/incomplete data. If everything is complete, this is to be piled in the box pending for data entry. The empty file folders will then be use for the next batch of subjects. The photocopies of the ID will also be filed.

Appendix -1

Transgenerational Study-Hyderabad

TRACKING FORM

1	SUBJECT ID:		
2	NAME OF VILLAGE:		
3	STUDY VILLAGE NO:		
4	WOMAN'S FAMILY NAME (surname):		
5	WOMAN'S FIRST (given) NAME:		
6	HUSBAND'S FIRST (given) NAME:		
7	HUSBAND'S SURNAME (if different from woman's surname):		
8	INTERVIEWER CODE:		
9	DATE OF QUESTIONNNAIRE COMPLETION:		
10	INFORMANT:		
11	STATUS OF THE FAMILY:	1 – STILL LIVING IN THE VILLAGE<br>2 – USED TO LIVE IN THE VILLAGE, BUT HAVE MOVED<br>3 – NEVER LIVED IN THE VILLAGE<br>4 – ANY OTHER	If Others(Specify)<br>_____<br>_____<br>_____
12	CURRENT POSTAL ADDRESS OF THE FAMILY (if any)		
13	TELEPHONE NUMBER	a) LANDLINE (with area code)	
		(b) Mobile	
14	AGE OF THE WOMAN (in completed years):		

DETAILS OF CHILDREN:

(a)Birth order	(b)First name	(c)Date of birth	(d)Gender 1=Male 2=Female	(e)Age	(f)Status	(g)Reason for move	(h)Education	(i)Occupation

Status:
1: Alive and resident in same village
2. Alive and moved to Hyderabad
3. Alive and moved relatively short distance (within 50 kms of Hyderabad, but not to Hyderabad.
4. Alive and moved relatively long distance (i.e. greater than 50 kms from Hyderabad.
5. Died
6. Any other, specify in comments.

Reason for Move:
(enter only if not staying with the family, otherwise leave blank)
Where there are multiple reasons, enter primary reason.
1: Education 2.Work 3. Marriage 4.Any other- specify in comments.

Comments:

_____

Study Recall: Parental recall of child being born in the study by virtue of -(Tick as appropriate)

1: Fieldworker visits (Questionnaires completed) -
2. Birth weight taken -
3. Both -
4. Neither -
5. Any other – specify-

Appendix -2

PARTICIPANT INFORMATION SHEET

Andhra Pradesh Children And Parents Study (APCAPS)
Nutritional challenges, abdominal adiposity and type 2 diabetes in Indians: Parental and offspring cardio-metabolic risk - a Transgenerational Extension of the Hyderabad Nutrition Trial

Purpose of the study
Researchers at the National Institute of Nutrition in Hyderabad and at the London School of Hygiene and Tropical Medicine are interested in understanding the effects of poor nutrition and environmental circumstances during pregnancy and childhood, on the chances of diabetes and heart disease occurring in the offspring. You have been chosen for this study as you/your wife/your mother participated in an earlier study conducted by National Institute of Nutrition. At this time, some but not all the participants were provided with extra food with the help of the Anganwadi. We are trying to know from this research whether the chances of getting heart diseases have been reduced in the children of women who got extra food. These comparisons will help to predict future needs for health services in India which will benefit planning. The research will also help in understanding the health effects – both positive and negative-of nutritional supplementation which may lead to ideas for preventing bad outcomes. Finally, the study provides an opportunity for you to gain important information about your health status.

Questions and concerns
You are being invited to participate in this medical research study. Kindly read this information sheet attentively. If you are not clear about anything or there is any uncertainty, then you are free to ask any questions when you receive a visit from the study staff. Sign the consent letter only when you are able to understand the nature of this study fully along with your rights as a participant. You are free to discuss it with anybody, whose consultation is important to you.

Voluntary participation
It is entirely your decision to participate in the study. If you want to discontinue at any point of time, you are free to leave this study without stating any reason. Your medical care will not be affected by your decision.

What does it mean to participate?
Participation in this study involves answering some questions about your general and medical habits, having body measurements taken, your blood drawn (**15ml)**, saliva (spits) sample taken and a short medical examination, which includes some measures of your heart and a breathing test. You will have a special type of X-ray taken to measure your body fat. Your answers are confidential and will be used only for the study.

You will be required to give a blood sample
You will be asked to fast overnight before the visit in which you give your blood samples. During the visit we will ask you to donate a small sample of your blood. Trained personnel will draw the blood. The supplies used for drawing blood will be safe and sterile and used only once and the supplies will be destroyed after use. The blood you give will be used for research purposes only. Any blood that is left over after the test will may be used for further test related to medical research including tests to find out whether any diseases run in the family.

Follow up in the future
The present research does not require the research team to see you again. However, important information can only be gained by linking your current health and life style to what happens to you in the future. Therefore, we would like to invite you to continue to participate in the future if you wish.

Benefits from the study
You will get a medical examination by an experienced team, including doctors. You will undergo the following tests: blood pressure, blood glucose for diabetes, haemoglobin, DXA scanning to measure fat levels, three tests on your heart and blood vessel function (arterial stiffness, pulse wave velocity and carotid intima media thickness), respiratory function and saliva sampling for genetic studies. Counselling will be given to you based on the results of the medical examination and blood tests. You will be given your blood results.

By participating in this study you will help researchers gain a clearer understanding of how nutritional supplementation has an effect on diseases like obesity and diabetes. Your participation will also help them understand how lifestyle, physical activity and dietary habits affect your health.

Risks of participating in the study
We do not expect that you will incur any risks by participating in this study. Blood drawing may cause a small amount of discomfort, but it is only temporary.

Financial costs
You will not incur any costs as a result of your participation in this study. Your travel fare along with any other expenses incurred and loss of daily wages towards time spent for our study will be reimbursed. Refreshments will also be provided.

Confidentiality
If you decide to take part in the study, all details provided by you will be kept confidential and it will only be made available to investigators related to this study. Information will be stored in password protected computer in Hyderabad and in London. The results will be published in research magazines and reports. However, the names and details of the study subjects will not be disclosed and you will not be recognized from them.

Funding & Coordinating agency
The funds for this study are being provided by the Wellcome Trust, a major UK based research charity.

Ethical Review
The study proposal has been approved by the London School of Hygiene & Tropical Medicine and the National Institute of Nutrition.

Contact for further information
If you require any further information or need to clarify some issue, you can contact any of our study team members at National Institute of Nutrition, Hyderabad

Tel: Project Office: National Institute of Nutrition: 040 – 27197256

Project Officer: Ms. Santhi Bhogadi: 9885448240.
Field Manager: Mr. Aniket Kumar: 7207805927

What your signature means
Your signature on the next page means that you understand the information given to you about the study. If you sign the form it means that you agree to join the study. You will be provided a copy of this patient information sheet to keep with your records.

Appendix -3

SECTION A: Consent Form

Andhra Pradesh Children And Parents Study (APCAPS)

Nutritional challenges, abdominal adiposity and type 2 diabetes in Indians: Parental and offspring cardio-metabolic risk - a Transgenerational Extension of the Hyderabad Nutrition Trial

Participant: Shri/Smt/Kum (First & Last Name)_____

Address (Lane, Town, State, Pincode)_____

I, _____exercising my free power of choice, hereby give my consent to be included as a subject in the clinical study - **Andhra Pradesh Children And Parents Study (APCAPS)** "Nutritional challenges, abdominal adiposity and type 2 diabetes in Indians"- Parental and offspring cardio-metabolic risk: - a Transgenerational Extension of the Hyderabad Nutrition Trial. (For the examination today we will ask you to undertake the following: interview, measurement of body size, DXA scan and a visit with the doctor. We will also ask you to give up to two blood samples. The examination will last until the afternoon.)

- I am free to participate or not to participate in this study.
- The purpose of this study was explained to me in my own language.
- I have been given the opportunity to ask questions and reply was given for all the questions to my satisfaction.
- I have been informed by the investigators about the process including the nature, objective and known likely inconveniences related to this study and I have understood them.
- My medical data are strictly confidential and I only authorise the persons, involved in the research, identified by the sponsor or health authorities to consult about the same.
- By signing this form, I give my free and informed consent to take part in this study as outlined in the information sheet and this consent form. Specifically, I agree to being interviewed, examined and having **(15 ml)** blood drawn. I agree to my information, including results of blood tests, to be used in research.
- I give permission for any blood that is left over after the tests to be stored and used for further laboratory tests for medical research
- I understand that for all practical purposes I may not gain anything by participating in the study though in the long run it may be beneficial to the community.
- I understand that I can withdraw from the study at any point without giving any reasons and withdrawing from the study will not affect me in any way.
- I understand that I will receive a very small radiation dose from the DXA scan, so I shouldn't have this scan if I am pregnant.
- I have been given a copy of the information sheet and consent form to keep. By signing this form I have not given up my legal rights.

Name of the Participant _____ Signature of the Participant_____

Date_____

Name of the Investigator_____ Signature of the Investigator_____

Date_____

SECTION B: Consent form for genetic component of the study

Andhra Pradesh Children And Parents Study (APCAPS)
Nutritional challenges, abdominal adiposity and type 2 diabetes in Indians: Parental and offspring cardio-metabolic risk - a Transgenerational Extension of the Hyderabad Nutrition Trial

Participant: Shri/Smt/Kum (First & Last Name) _____

Address (Lane, Town, State, Pin Code)_____

I, _____exercising my free power of choice, hereby give my consent to be included as a subject in the clinical study - **Andhra Pradesh Children And Parents Study (APCAPS)** "Nutritional challenges, abdominal adiposity and type 2 diabetes in Indians"- Parental and offspring cardio-metabolic risk: - a Transgenerational Extension of the Hyderabad Nutrition Trial. (For the examination today we will ask you to undertake the following: interview, measurement of body size, DXA scan and a visit with the doctor. We will also ask you to give up to two blood samples. The examination will last until the afternoon.)

- I am free to participate or not to participate in this study.
- The purpose of this study was explained to me in my own language.
- I have been given the opportunity to ask questions and reply was given for all the questions to my satisfaction.
- I have been informed by the investigators about the process including the nature, objective and known likely inconveniences related to this study and I have understood them.
- My medical data are strictly confidential and I only authorise the persons, involved in the research, identified by the sponsor or health authorities to consult about the same.
- By signing this form, I give my free and informed consent to take part in this study as outlined in the information sheet and this consent form. Specifically, I agree to being interviewed, examined, having saliva samples taken and blood drawn **(15ml)** for necessary tests. I agree to my information, including results of blood tests, to be used in research.
- I understand that the saliva sample and blood sample may be used for genetic research aimed at understanding genetic and epigenetic influences on diseases.
- I understand that for all practical purposes I may not gain anything by participating in the study though in the long run it may be beneficial to the community.
- I understand that I can withdraw from the study at any point without giving any reasons and withdrawing from the study will not affect me in any way.
- I have been given a copy of the information sheet and consent form to keep. By signing this form I have not given up my legal rights.

Name of the Participant _____ Signature of the Participant _____

Date_____

Name of the Investigator_____ Signature of the Investigator_____

Date_____

Appendix -4

Andhra Pradesh Children And Parents Study (APCAPS)

A Transgenerational extension of Hyderabad Nutrition Trial

(Funded by the Wellcome Trust, U.K)

CLINIC QUESTIONNAIRE

**Apply Subject details Label here**	**Apply Study ID Label here**

Subject Type : ☐

1 = Mother

2 = Father

3 = Sibling

4 = DXA

Participant info:

Name: _____

Date of birth: _____/_____/_____

Consent form	☐ [1=Yes; 2=No]	Lung function test	☐ [1=Yes; 2=No]
Blood	☐ [1=Yes; 2=No]	Vascular studies-NIN	☐ [1=Yes; 2=No]
Saliva	☐ [1=Yes; 2=No]	Spirometry -NIN	☐ [1=Yes; 2=No]
Questionnaire	☐ [1=Yes; 2=No]	DXA - NIN	☐ [1=Yes; 2=No]
Anthropometry	☐ [1=Yes; 2=No]	Doctor	☐ [1=Yes; 2=No]
TANITA	☐ [1=Yes; 2=No]	Reimbursement	☐ [1=Yes; 2=No]

Supported by

NATIONAL INSTITUTE OF NUTRITION, HYDERABAD, India
LONDON SCHOOL OF HYGIENE & TROPICAL MEDICINE, U.K.
&
UNIVERSITY OF BRISTOL, U.K.

PARTICIPANT INFORMATION SHEET

Andhra Pradesh Children And Parents Study (APCAPS)

Nutritional challenges, abdominal adiposity and type 2 diabetes in Indians: Parental and offspring cardio-metabolic risk - a Transgenerational Extension of the Hyderabad Nutrition Trial

Purpose of the study

Researchers at the National Institute of Nutrition in Hyderabad and at the London School of Hygiene and Tropical Medicine are interested in understanding the effects of poor nutrition and environmental circumstances during pregnancy and childhood, on the chances of diabetes and heart disease occurring in the offspring. You have been chosen for this study as you/your wife/your mother participated in an earlier study conducted by National Institute of Nutrition. At this time, some but not all the participants were provided with extra food with the help of the Anganwadi. We are trying to know from this research whether the chances of getting heart diseases have been reduced in the children of women who got extra food. These comparisons will help to predict future needs for health services in India which will benefit planning. The research will also help in understanding the health effects –both positive and negative-of nutritional supplementation which may lead to ideas for preventing bad outcomes. Finally, the study provides an opportunity for you to gain important information about your health status.

Questions and concerns

You are being invited to participate in this medical research study. Kindly read this information sheet attentively. If you are not clear about anything or there is any uncertainty, then you are free to ask any questions when you receive a visit from the study staff. Sign the consent letter only when you are able to understand the nature of this study fully along with your rights as a participant. You are free to discuss it with anybody, whose consultation is important to you.

Voluntary participation

It is entirely your decision to participate in the study. If you want to discontinue at any point of time, you are free to leave this study without stating any reason. Your medical care will not be affected by your decision.

What does it mean to participate?

Participation in this study involves answering some questions about your general and medical habits, having body measurements taken, your blood drawn (**15ml)**, saliva (spits) sample taken and a short medical examination, which includes some measures of your heart and a breathing test. You will have a special type of X-ray taken to measure your body fat. Your answers are confidential and will be used only for the study.

You will be required to give a blood sample

You will be asked to fast overnight before the visit in which you give your blood samples. During the visit we will ask you to donate a small sample of your blood. Trained personnel will draw the blood. The supplies used for drawing blood will be safe and sterile and used only once and the supplies will be destroyed after use. The blood you give will be used for research purposes only. Any blood that is left over after the test will may be used for further test related to medical research including tests to find out whether any diseases run in the family.

Follow up in the future

The present research does not require the research team to see you again. However, important information can only be gained by linking your current health and life style to what happens to you in the future. Therefore, we would like to invite you to continue to participate in the future if you wish.

Benefits from the study

You will get a medical examination by an experienced team, including doctors. You will undergo the following tests: blood pressure, blood glucose for diabetes, haemoglobin, DXA scanning to measure fat levels, three tests on your heart and blood vessel function (arterial stiffness, pulse wave velocity and carotid intima media thickness), respiratory function and saliva sampling for genetic studies. Counselling will be given to you based on the results of the medical examination and blood tests. You will be given your blood results.

By participating in this study you will help researchers gain a clearer understanding of how nutritional supplementation has an effect on diseases like obesity and diabetes. Your participation will also help them understand how lifestyle, physical activity and dietary habits affect your health.

Risks of participating in the study
We do not expect that you will incur any risks by participating in this study. Blood drawing may cause a small amount of discomfort, but it is only temporary.

Financial costs
You will not incur any costs as a result of your participation in this study. Your travel fare along with any other expenses incurred and loss of daily wages towards time spent for our study will be reimbursed. Refreshments will also be provided.

Confidentiality
If you decide to take part in the study, all details provided by you will be kept confidential and it will only be made available to investigators related to this study. Information will be stored in password protected computer in Hyderabad and in London. The results will be published in research magazines and reports. However, the names and details of the study subjects will not be disclosed and you will not be recognized from them.

Funding & Coordinating agency
The funds for this study are being provided by the Wellcome Trust, a major UK based research charity.

Ethical Review
The study proposal has been approved by the London School of Hygiene & Tropical Medicine and the National Institute of Nutrition.

Contact for further information
If you require any further information or need to clarify some issue, you can contact any of our study team members at National Institute of Nutrition, Hyderabad

Tel: Project Office: National Institute of Nutrition: 040 – 27197256

Project Officer: Ms. Santhi Bhogadi: 9885448240.
Field Manager: Mr. Aniket Kumar: 7207805927

What your signature means
Your signature on the next page means that you understand the information given to you about the study. If you sign the form it means that you agree to join the study. You will be provided a copy of this patient information sheet to keep with your records.

SECTION A: Consent Form

Andhra Pradesh Children And Parents Study (APCAPS)

Nutritional challenges, abdominal adiposity and type 2 diabetes in Indians: Parental and offspring cardio-metabolic risk - a Transgenerational Extension of the Hyderabad Nutrition Trial

Participant: Shri/Smt/Kum (First & Last Name)_____

Address (Lane, Town, State, Pincode)_____

I, _____exercising my free power of choice, hereby give my consent to be included as a subject in the clinical study - **Andhra Pradesh Children And Parents Study (APCAPS)** "Nutritional challenges, abdominal adiposity and type 2 diabetes in Indians"- Parental and offspring cardio-metabolic risk: - a Transgenerational Extension of the Hyderabad Nutrition Trial.
(For the examination today we will ask you to undertake the following: interview, measurement of body size, DXA scan and a visit with the doctor. We will also ask you to give up to two blood samples. The examination will last until the afternoon.)

- I am free to participate or not to participate in this study.
- The purpose of this study was explained to me in my own language.
- I have been given the opportunity to ask questions and reply was given for all the questions to my satisfaction.
- I have been informed by the investigators about the process including the nature, objective and known likely inconveniences related to this study and I have understood them.
- My medical data are strictly confidential and I only authorise the persons, involved in the research, identified by the sponsor or health authorities to consult about the same.
- By signing this form, I give my free and informed consent to take part in this study as outlined in the information sheet and this consent form. Specifically, I agree to being interviewed, examined and having **(15 ml)** blood drawn. I agree to my information, including results of blood tests, to be used in research.
- I give permission for any blood that is left over after the tests to be stored and used for further laboratory tests for medical research
- I understand that for all practical purposes I may not gain anything by participating in the study though in the long run it may be beneficial to the community.
- I understand that I can withdraw from the study at any point without giving any reasons and withdrawing from the study will not affect me in any way.
- I understand that I will receive a very small radiation dose from the DXA scan, so I shouldn't have this scan if I am pregnant.
- I have been given a copy of the information sheet and consent form to keep. By signing this form I have not given up my legal rights.

Name of the Participant _____ Signature of the Participant_____

Date_____

Name of the Investigator_____ Signature of the Investigator_____

Date_____

SECTION B: Consent form for genetic component of the study

Andhra Pradesh Children And Parents Study (APCAPS)

Nutritional challenges, abdominal adiposity and type 2 diabetes in Indians: Parental and offspring cardio-metabolic risk - a Transgenerational Extension of the Hyderabad Nutrition Trial

Participant: Shri/Smt/Kum (First & Last Name) _____

Address (Lane, Town, State, Pin Code)_____

I, _____exercising my free power of choice, hereby give my consent to be included as a subject in the clinical study - **Andhra Pradesh Children And Parents Study (APCAPS)** "Nutritional challenges, abdominal adiposity and type 2 diabetes in Indians"- Parental and offspring cardio-metabolic risk: - a Transgenerational Extension of the Hyderabad Nutrition Trial.
(For the examination today we will ask you to undertake the following: interview, measurement of body size, DXA scan and a visit with the doctor. We will also ask you to give up to two blood samples. The examination will last until the afternoon.)

- I am free to participate or not to participate in this study.
- The purpose of this study was explained to me in my own language.
- I have been given the opportunity to ask questions and reply was given for all the questions to my satisfaction.
- I have been informed by the investigators about the process including the nature, objective and known likely inconveniences related to this study and I have understood them.
- My medical data are strictly confidential and I only authorise the persons, involved in the research, identified by the sponsor or health authorities to consult about the same.
- By signing this form, I give my free and informed consent to take part in this study as outlined in the information sheet and this consent form. Specifically, I agree to being interviewed, examined, having saliva samples taken and blood drawn **(15ml)** for necessary tests. I agree to my information, including results of blood tests, to be used in research.
- I understand that the saliva sample and blood sample may be used for genetic research aimed at understanding genetic and epigenetic influences on diseases.
- I understand that for all practical purposes I may not gain anything by participating in the study though in the long run it may be beneficial to the community.
- I understand that I can withdraw from the study at any point without giving any reasons and withdrawing from the study will not affect me in any way.
- I have been given a copy of the information sheet and consent form to keep. By signing this form I have not given up my legal rights.

Name of the Participant _____ Signature of the Participant _____

Date_____

Name of the Investigator_____ Signature of the Investigator_____

Date_____

NATIONAL INSTITUTE OF NUTRITION (ICMR)

JAMAI OSMANIA (P.O.), HYDERABAD - 500 007

భారతీయులలో పోషకాహార సమస్యలు, ఉదర భాగమునందు క్రొవ్వు చేరుట మరియు టైపు2 మధుమేహము - తల్లిదండ్రులు మరియు వారి పిల్లలకు హృదయతత్వ పరిణామములకు అపాయము : తరతరాల మార్పులు, హైదరాబాదు పోషకాహార శోధనకు పొడిగింపు.

(Study title : "Nutritional Challanges, Abdominal Adiposity and type 2 diabetes in Indians - parental and offspring cardio - metabolic risk : a trans - generational extension of Hyderabad nutrition trial)

పాల్గొను వారికి విషయ సూచిక

అధ్యయనం యొక్క ఆవశ్యకత :

తల్లి గర్భములో మరియు చిన్నతనములో పౌష్టికాహార లోపము మరియు పరిసరాల యొక్క ప్రభావము వలన పిల్లల్లో మధుమేహము మరిము గుండె జబ్బులు వచ్చుటకు గల అవకాశములపై జాతీయ పోషకాహార సంస్థ, హైదరాబాదు మరియు లండన్ స్కూల్ ఆఫ్ హైజీన్ మరియు ట్రాపికల్ మెడిసిన్‌లో పనిచేయు పరిశోధకులు అధ్యయనం చేయదలచినారు. మీరు/ మీ భార్య / మీ అమ్మ గతంలో జాతీయ పోషకాహార సంస్థ నిర్వహించి అధ్యయనములో పాల్గొనినారు కావున మిమ్మల్ని ఈ అధ్యయనములో పాల్గొనుటకు ఎంచుకొనినారు. ఈ సమయములో కొంతమంది చూలింతలకు అంగన్‌వాడి ద్వారా అధిక ఆహారం ఇవ్వబడినది. ఈ అధ్యయనం ద్వారా అధిక ఆహారము తీసుకొనిన వారికి పుట్టిన పిల్లలకు గుండెకు సంబంధించిన జబ్బులు వచ్చుటకు అవకాశము తక్కువగా ఉండునేమో అని తెలుసుకొనదలచినారు. ఈ అనుసంధానము వలన భవిష్యత్‌లో భారతదేశములో ఆరోగ్యపరమైన సేవలు చేయుటకు దోహదపడును. ఈ పరిశోధన వలన ఆరోగ్యముపై ప్రభావమును అర్థం చేసుకొనుటకు అధిక పౌష్టికాహారము వలన కలుగు మంచి/ చెడు ప్రభావములు తెలుసుకొని, చెడు ప్రభావములను నివారించడానికి దోహదపడును. చివరిగా ఈ అధ్యయనములో మీ ప్రస్తుత ఆరోగ్య పరిస్థితి తెలుసుకొనుటకు అవకాశము లభించును.

ప్రశ్నలు మరియు నిమిత్తములు :

మిమ్మల్ని వైద్య సంబంధ పరిశోధన చేయు అధ్యయనములో పాల్గొనుటకు ఆహ్వానించుచున్నాము. దయచేసి ఈ విషయ సూచికను జాగ్రత్తగా చదువగలరు. మీకు ఈ ఏ విషయమైన అర్థం కాని ఎడల లేదా ఏ విషయముపైననైన అనుమానం ఉన్నచో, అధ్యయనములో పనిచేయు ఉద్యోగస్తులను అడిగి తెలుసుకొనుటకు మీరు పూర్తి స్వేచ్ఛ కలిగి ఉన్నారు. ఈ అధ్యయనము యొక్క స్వభావముతో పాటు మీరు పాల్గొనుటకు గల అధికారము పూర్తిగా అర్థమైన తరువాతనే అంగీకార పత్రముపై సంతకము చేయగలరు. ఈ విషయములు మీరు మీకు ముఖ్యమైన వారితో చర్చించుటకు పూర్తి స్వేచ్ఛను కలిగి ఉన్నారు.

పాల్గొనుటకు స్వేచ్ఛ :

ఈ అధ్యయనములో పాల్గొనుటకు పూర్తిగా మీ నిర్ణయం. ఏ నమయములోనైనా మీరు ఈ అధ్యయనము నుండి విరమించుకొనదలచినచో, ఎవ్వరికి ఏ కారణము తెలుపకుండానే ఈ అధ్యయనము నుండి తప్పుకొనవచ్చును. ఈ నిర్ణయము వలన మీ వైద్య సంరక్షణ మరియు ఉద్యోగముపై ఎటువంటి ప్రభావము ఉండదు.

పాల్గొనుట అనగానేమి?

ఈ అధ్యయనములో పాల్గొనుట అనగా మీరు మీ వైద్య సంబంధ అలవాట్లకు సంబంధజంచిన ప్రశ్నలకు సమాధానములు తెలుపుట. మీ శరీర కొలతలను తీసుకొనుట. 15 మి.లీ. రక్తము మరియు ఉమ్మి నమూన తీసుకొనుట మరియు గుండె, గాలి తీసుకొను ఎక్స్‌రేను తీయించుకొనదరు. మీ సమాధానములు గుప్తముగా ఉంచబడును మరియు ఈ అధ్యయనమునకు మాత్రమే ఉపయోగించుకొనబడును.

మీరు రక్త నమూన ఇవ్వవలసి ఉండును :

రక్త నమూన కొరకు మిమ్మల్ని రాత్రి నుండి ఏమి ఆహారము తీసుకొనకుండా ఉండమని అడిగెదము. మీరు వచ్చిన తరువాత ఒక చిన్న రక్త నమూనా తీసుకొనదము. ప్రజ్ఞావంతులైన నిపుణులచే రక్తము తీయించబడును. మీరు ఇచ్చిన రక్తము పరిశోధనకు మాత్రమే ఉపయోగించబడును.

భవిష్యత్ విచారణ :

ప్రస్తుత పరిశోధనకు, పరిశోధకులు ఎవ్వరు మిమ్మల్ని మరల చూడనవసరము లేదు. కాని, మీ ప్రస్తుత ఆరోగ్యము మరియు జీవన విధానము, భవిష్యత్ కాలములో మీకు ఎలా ఉండును అన్న విషయములను అనుసంధానము చేయుటచే ముఖ్య సమాచారము లభించును. అందువలన, మిమ్మల్ని మరల భవిష్యత్‌లో కూడా పాల్గొనుటకు ఆహ్వానించుచున్నాము.

అధ్యయనము వలన లాభములు :

మీకు అనుభవజ్ఞులైన వైద్యులచే వైద్య పరీక్షలు నిర్వహించబడును. మీకు బి.పి., మధుమేహము కొరకు షగరు పరీక్ష, రక్త శాతము, కొవ్వును కొలువు డెక్సా స్కానింగ్, గుండె మరియు రక్త నాళముల పనితీరును కనుగొనుటకు (ఆర్‌టీర్యల్ స్టిఫ్‌నెస్, ఫల్స్‌వేవ్ వెలాసిటీ, కరోటిడ్ ఇంటిమా మీడియా తిక్‌నెస్) మరియు ఉచ్ఛ్వాస, నిచ్ఛ్వాస క్రియల పరీక్షలు చేయబడును. మీ వైద్య మరియు రక్త పరీక్ష యొక్క ఫలితములను ఇవ్వబడును. మరియు వాటి ఆధారముగా తగిన సల హాలను ఇవ్వబడును. ఈ అధ్యయనములో పాల్గొనుట వలన పరిశోధకులకు అధిక పౌష్టికాహారము ఇవ్వడం వలన ఊబకాయము, మధుమేహము వంటి రోగములకు సంబంధము గురించి తెలుసుకొనుటకు అవకాశము కలుగును. మీరు ఈ అధ్యయనములో పాల్గొనుట వలన జీవన విధానము, స్వాభావిక విధానము మరియు ఆహార లక్షణములు మీ ఆరోగ్యముపై ప్రభావమును అర్థం చేసుకొనుటకు దోహదపడును.

అధ్యయనములో పాల్గొనుట వలన కలుగు అపాయములు :

ఈ అధ్యయనములో పాల్గొనుట వలన మీకు ఎటువంటి అసౌకర్యము కలుగునని భావించుట లేదు. రక్తము తీయునప్పుడు కొంచెము అసౌకర్యముగా అనిపించవచ్చును. కాని ఇది తాత్కాలికము.

ఆర్థిక వరమైన ఖర్చులు :

ఈ అధ్యయనములో పాల్గొనుట వలన మీకు ఎటువంటి ఆర్థికపరమైన ఇబ్బందులు కలుగవు. మీ ప్రయాణ ఛార్జీలు మరియు మీ పనిని మానుకొని ఆ సమయమును ఈ అధ్యయనము కొరకు వెచ్చించినందుకుగాను మీకు తగిన పరిహారము ఇవ్వబడును. ఆహారము కూడా ఇవ్వబడును.

గోప్యము :

ఈ అధ్యయనములో మీరు పాల్గొనదలచిన, మీకు సంబంధించిన వివరములన్నీ గోప్యముగా ఉంచబడును. మరియు ఆ వివరములన్నీ ఈ అధ్యయనములో పనిచేయు వారికి మాత్రమే తెలియును. మీ వివరములన్నీ హైదరాబాదు మరియు లండన్‌లో పాస్‌వర్డ్ ఉన్న కంప్యూటర్‌లో భద్రపరచబడును. ఈ ఫలితములు పరిశోధనా పత్రికలలో మరియు సమాచార పత్రములలో ముద్రించబడును. కాని, ఈ అధ్యయములో పాల్గొనువారి పేర్లు మరియు ఇతర వివరములేవి తెలియరావు, మిమ్మల్ని ఈ వివరముల వలన గుర్తించుట జరుగదు.

ఆర్థిక సహకారము మరియు తుల్య సంస్థ :

ఈ అధ్యయనమునకు ఆర్థిక సహకారము వెల్‌కంట్రస్ట్, యు.కె. అను పరిశోధన దాతృత్వ సంస్థ అందించుచున్నది.

నైతికవరమైన సమీక్ష :

ఈ అధ్యయన యోచనను లండన్ స్కూల్ ఆఫ్ హైజీన్ మరియు ట్రాఫికల్ మెడిసిన్ మరియు జాతీయ పోషకాహార సంస్థ వారిచే ఆమోదించబడినది.

సమాచారము తెలుసుకొనుట :

ఇతరత్ర ఏ విషయమైన తెలుసుకొనదలచిన లేదా ఏ విషయమైన విశదపరచుటకు మీరు ఈ అధ్యయనములకు సంబంధించిన ఉద్యోగస్థులను జాతీయ పోషకాహార సంస్థ, హైదరాబాద్‌లో సంప్రదించవచ్చును.

ఫోన్ నెంబర్లు :

ప్రాజెక్ట్ ఆఫీసు : జాతీయ పోషకాహార సంస్థ : 040-27197256
ప్రాజెక్ట్ ఆఫీసర్ : శాంతి భోగాది : 9885448240
ఫీల్డ్ మేనేజర్ : అనికేత్ కుమార్ : 7207805927

మీ సంతకము అనగా :

తదుపరి పత్రముపై మీ సంతకము అనగా మీరు ఈ సమాచార పత్రమును అర్థం చేసుకొనివారు అని భావించెదము. ఈ పత్రముపై సంతకము అనగా ఈ అధ్యయనములో పాల్గొనుటకు అంగీకరించిరని భావించెదము. మీకు అధ్యయనము యొక్క సమాచార పత్రము ఇవ్వబడును.

NATIONAL INSTITUTE OF NUTRITION (ICMR)

JAMAI OSMANIA (P.O.), HYDERABAD - 500 007

.భారతీయులలో పోషకాహార సమస్యలు, ఉదర భాగమునందు క్రొవ్వు చేరుట మరియు టైపు2 మధుమేహము - తల్లిదండ్రులు మరియు వారి పిల్లలకు హృదయతత్వ పరిణామములకు అపాయము : తరతరాల మార్పులు, హైదరాబాదు పోషకాహార శోధనకు పొడిగింపు.

(Study title : "Nutritional Challanges, Abdominal Adiposity and type 2 diabetes in Indians - parental and offspring cardio - metabolic risk : a trans - generational extension of Hyderabad nutrition trial)

'ఏ' భాగము అంగీకార పత్రము

పాల్గొను వారు :

శ్రీ / శ్రీమతి / కుమారి (మొదటి అండ్ చివరి పేరు)

చిరునామ (వీధి, ఊరు పేరు, రాష్ట్రము, పిన్ నెంబరు)

నేను ..... ("భారతీయులలో పోషకాహార సమస్యలు, ఉదర భాగమునందు కొవ్వు మరియు టైపు 2 మధుమేహం") వ్యాధులపై జరుపుచున్న అధ్యయనములో పాల్గొనుటకు నా సమ్మతిని, స్వేచ్ఛను ఉపయోగించుకుంటున్నాను. (ఈ రోజు జరుగుచున్న ఈ పరీక్షలో మిమ్మల్ని మేము ఇంటర్వ్యూవ ఇవ్వమ ని, శరీర కొలతలు, డాక్సాస్కాన్ మరియు డాక్టరు చేత పరీక్ష చేయించుకొనమని కోరెదము. రెండు రక్తము మరియు ఉమ్మి నమూనాలు ఇవ్వమని అడిగెదము. ఈ పరీక్షలన్నీ మధ్యాహ్నం వరకు జరుగును.)

- ఈ అధ్యయనములో పాల్గొనుట పాల్గొనకపోవుటను నిర్ణయించుకొనుటకు నాకు స్వేచ్ఛ కలదు.
- ఈ అధ్యయనము యొక్క ఆవశ్యకతను నా మాతృభాషలో వివరించినారు.
- ప్రశ్నలు వేయడానికి నాకు అవకాశమిచ్చి, అన్ని ప్రశ్నలకు సంతృప్తికరమైన సమాధానములు ఇచ్చినారు.
- అధ్యయనము యొక్క విధానము, స్వభావము, ఉద్దేశము మరియు తెలిసిన, కలుగు అసౌకర్యముల గురించి ఈ అధ్యయనములో పనిచేయువారు తెలియజేసినారు. నేను అర్థం చేసుకున్నాను.
- నా వైద్య సంబంధమ ఐన సమాచారమంతయు అతి రహస్యముగా మరియు పరిశోధనకు సహకరించు వారిచే గుర్తింపు పొందిన వారు లేక ఆరోగ్య సంబంధిత అధికారులకు మాత్రమే, తెలుసుకొనుటకు అవకాశము ఇస్తాను.
- ఈ పత్రముపై సంతకం చేయడం ద్వారా, సమాచార మరియు సమ్మతి పత్రములో రూపొందించిన విషయములపై మరియు తెలియపరచిన వాటిపై, నన్ను ప్రశ్నించడానికి, పరీక్షించటానికి మరియు ఉమ్మి, రక్త నమూన (15మి.లీ) తీసుకొనుటకు నా సమ్మతిని తెలియజేయుచున్నాను. నా సమాచారము, రక్త పరీక్ష ఫలితాలను పరిశోధనకు ఉపయోగించవచ్చును.
- పరీక్ష తరువాత మిగిలిన రక్తమును, నిల్వచేయుటకు మరియు వైద్య పరిశోధనలకై జరిపే పరీక్షలకు ఉపయోగించుకొనుటకు నేను అనుమతిస్తాను.
- ఈ అధ్యయనము దీర్ఘకాలంలో సమాజానికి ఉపయోగపడినప్పటికిని, నాకు మాత్రము ఎలాంటి లాభం కలుగదని తెలుసును.
- ఈ అధ్యయనము నుండి ఏ కారణము తెలుపకుండా ఎప్పుడైనా విరమించుకొనవచ్చునని నేను అర్థం చేసుకున్నాను, మరియు విరమించుకోవడం వల్ల నాపై ఎటువంటి ప్రభావము ఉండదు.
- డెక్సా స్కానింగ్ నుండి చిన్నపాటి కిరణాలను గ్రహించవలసి ఉండును కావున, నేను గర్భవతిని అయినచో ఈ స్కానింగ్ చేయించుకోకూడదు అని అర్థం చేసుకొనినాను.
- విషయ సూచిన మరియు అంగీకార పత్రము ఉంచుకొనుటకు నాకు ఇవ్వబడినది. ఈ పత్రముపై సంతకము చేయడం వలన నా న్యాయపరమైన హక్కులను కోల్పోను.

పాల్గొను వారి పేరు .....

పాల్గొను వారి సంతకం ..... తేది : .....

ఇన్వెస్టిగేటర్ పేరు : .....

ఇన్వెస్టిగేటర్ సంతకము : .....తేది :.....

NATIONAL INSTITUTE OF NUTRITION (ICMR)

JAMAI OSMANIA (P.O.), HYDERABAD - 500 007

భారతీయులలో పోషకాహార సమస్యలు, ఉదర భాగమునందు క్రొవ్వు చేరుట మరియు టైపు2 మధుమేహము - తల్లిదండ్రులు మరియు వారి పిల్లలకు హృదయతత్వ పరిణామములకు అపాయము : తరతరాల మార్పులు, హైదరాబాదు పోషకాహార శోధనకు పొడిగింపు.

(Study title : "Nutritional Challanges, Abdominal Adiposity and type 2 diabetes in Indians - parental and offspring cardio - metabolic risk : a trans - generational extension of Hyderabad nutrition trial)

'బి' భాగము : జన్యునంబంధిత విభాగము కొరకు అంగీకార పత్రము

పాల్గొను వారు :

శ్రీ / శ్రీమతి / కుమారి (మొదటి అండ్ చివరి పేరు)

చిరునామ (వీధి, ఊరు పేరు, రాష్ట్రము, పిన్ నెంబరు)

నేను ..... ("భారతీయులలో పోషకాహార సమస్యలు, ఉదర భాగమునందు కొవ్వు మరియు టైపు 2 మధుమేహం") వ్యాధులపై జరుపుచున్న అధ్యయనములో పాల్గొనుటకు నా సమ్మతిని, స్వేచ్ఛను ఉపయోగించుకుంటున్నాను. (ఈ రోజు జరుగుచున్న ఈ పరీక్షలో మిమ్మల్ని మేము ఇంటర్వ్యూవ ఇవ్వమ ని, శరీర కొలతలు, డాక్సాస్కాన్ మరియు డాక్టరు చేత పరీక్ష చేయించుకొనమని కోరెదము. రెండు రక్తము మరియు ఉమ్మి నమూనాలు ఇవ్వమని అడిగెదము. ఈ పరీక్షలన్నీ మధ్యాహ్నం వరకు జరుగును.)

- ❄ ఈ అధ్యయనములో పాల్గొనుట పాల్గొనకపోవుటను నిర్ణయించుకొనుటకు నాకు స్వేచ్ఛ కలదు.
- ❄ ఈ అధ్యయనము యొక్క ఆవశ్యకతను నా మాతృభాషలో వివరించినారు.
- ❄ ప్రశ్నలు వేయడానికి నాకు అవకాశమిచ్చి, అన్ని ప్రశ్నలకు సంతృప్తికరమైన సమాధానములు ఇచ్చినారు.
- ❄ అధ్యయనము యొక్క విధానము, స్వభావము, ఉద్దేశము మరియు తెలిసిన, కలుగు అసౌకర్యముల గురించి ఈ అధ్యయనములో పనిచేయువారు తెలియజేసినారు. నేను అర్థం చేసుకున్నాను.
- ❄ నా వైద్య సంబంధమ ఐన సమాచారమంతయు అతి రహస్యముగా మరియు పరిశోధనకు సహకరించు వారిచే గుర్తింపు పొందిన వారు లేక ఆరోగ్య సంబంధిత అధికారులకు మాత్రమే, తెలుసుకొనుటకు అవకాశము ఇస్తాను.
- ❄ ఈ పత్రముపై సంతకం చేయడం ద్వారా, సమాచార మరియు సమ్మతి పత్రములో రూపొందించిన విషయములపై మరియు తెలియపరచిన వాటిపై, నన్ను ప్రశ్నించడానికి, పరీక్షించటానికి మరియు ఉమ్మి, రక్త నమూన 15 మి॥లీ తీసుకొనుటకు నా సమ్మతిని తెలియజేయుచున్నాను. నా సమాచారము, రక్త పరీక్ష ఫలితాలను పరిశోధనకు ఉపయోగించవచ్చును.
- ❄ నా ఉమ్మి మరియు రక్త నమూన, రోగములపై జన్యు మరియు వంశపారంపర్యగా వచ్చుచున్న జన్యు సంబంధిత మార్పుల యొక్క ప్రభావమును అధ్యయనము చేయు జన్యు పరిశోధనకు ఉపయోగించెదరని అర్థం చేసుకొనినాను.
- ❄ ఈ అధ్యయనము ధీర్ఘకాలంలో సమాజానికి ఉపయోగపడినప్పటికిని, నాకు మాత్రము ఎలాంటి లాభం కలుగదని తెలుసును.
- ❄ ఈ అధ్యయనము నుండి ఏ కారణము తెలుపకుండా ఎప్పుడైనా విరమించుకొనవచ్చునని నేను అర్థం చేసుకున్నాను, మరియు విరమించుకోవడం వల్ల నాపై ఎటువంటి ప్రభావము ఉండదు.
- ❄ డెక్సా స్కానింగ్ నుండి చిన్నపాటి కిరణాలను గ్రహించవలసి ఉండును కావున, నేను గర్భవతిని అయినచో ఈ స్కానింగ్ చేయించుకోకూడదు అని అర్థం చేసుకొనినాను.
- ❄ విషయ సూచిన మరియు అంగీకార పత్రము ఉంచుకొనుటకు నాకు ఇవ్వబడినది. ఈ పత్రముపై సంతకము చేయడం వలన నా న్యాయపరమైన హక్కులను కోల్పోను.

పాల్గొను వారి పేరు .....

పాల్గొను వారి సంతకం ..... తేది : .....

ఇన్వెస్టిగేటర్ పేరు : .....

ఇన్వెస్టిగేటర్ సంతకము : .....తేది :.....

SECTION A: Reimbursement

	Summary sheet (to be completed at time of reimbursement)		
	Reimbursement		
1.1	Reimbursement given	☐ [1=Yes; 2=No]	
1.2	Identity proof taken	☐ [1=Yes; 2=No]	
	Subject recall		
1.3	Subject needs to be recalled	☐ [1=Yes; 2=No]	
1.4	Recall for repeatability study	☐ [1=Yes; 2=No]	
1.5	Recall for validation study	☐ [1=Yes; 2=No]	
1.6	Recall for incomplete study	☐ [1=Yes; 2=No]	
1.7	If yes, is the subject willing to return?	☐ [1=Yes; 2=No; 3=Undecided]	
1.8	If undecided, date status will be reviewed:	__ __/__ __/__ __ [DD/MM/YY]	
1.9	If recalled, clinic visit details		
	(a) Date of clinic visit [DD/MM/YY]	(b) Travel [1=Self; 2=Team]	(c) Outcome [1=Yes; 2=No]
	__ __/__ __/__ __		
	__ __/__ __/__ __		
	__ __/__ __/__ __		
1.10	Summary sheet notes		
	Accelerometer		
1.11	Accelerometer number	☐☐	
1.12	Date of initiation	___ ___ / ___ ___ / ___ ___ [DD/MM/YY]	
1.13	Time of initiation	☐☐ : ☐☐ [Hours: minutes; 24-hour clock]	
1.14	Date of termination	___ ___ / ___ ___ / ___ ___ [DD/MM/YY]	
1.15	Time of termination	☐☐ Hours ☐☐ Minutes	
1.16	Comments	_____ _____	

SECTION B: Blood Sampling

	Blood sampling		
2.1	Any illness within the last week?	☐ [1=Yes; 2=No]	
2.2	If yes, specify what illness: Cold ☐ Cough ☐ Headache ☐ Fever ☐ Bodyaches ☐ Pain Abdomen ☐ Diarrhoea ☐ Vomiting ☐ Others ☐ Specify_____		
2.3	(a) Was this illness or some other reason responsible for reduction in food intake over the last week?	☐ 1=No reduction<br>2=Minor reduction<br>3=Major reduction	
	(b) Do you have diabetes?	☐ [1=Yes; 2=No]	
	(c) Are you pregnant?	☐ [1=Yes; 2=No]	
2.4	Day of last meal	☐ [1=Today; 2=Yesterday]	
2.5	Time of last meal	☐☐ : ☐☐ [Hours: minutes; 24-hour clock]	
2.6	Time blood sample taken:	☐☐ : ☐☐ [Hours: minutes; 24-hour clock]	
2.7	Saliva sample taken	☐ [1=Yes; 2=No]	
2.8			
2.9			
	Success in blood sampling	(a) Volume	(b) Clot formation
2.10	Red capped tube	☐ [1=No; 2=Partial; 3=Complete]	☐ [1=Yes; 2=No]
2.11	Purple capped tube 1	☐ [1=No; 2=Partial; 3=Complete]	☐ [1=Yes; 2=No]
2.12	Grey capped tube	☐ [1=No; 2=Partial; 3=Complete]	☐ [1=Yes; 2=No]
2.13	Purple capped tube 2	☐ [1=No; 2=Partial; 3=Complete]	☐ [1=Yes; 2=No]
2.15	(a) Any other comments on blood sample	☐ [1=Yes; 2=No]	
	(b) If yes, specify	_____	

SECTION C: Clinical Questionnaire

	Interview details	
3.1	Date of quest. completion	___ ___ / ___ ___ / ___ ___ [DD/MM/YY]
3.2a.	Time of quest. Starting	☐☐ : ☐☐ [Hours: minutes; 24-hour clock]
3.3	Interviewer code	☐☐
3.4	Interviewer initials	☐☐☐
	First of all I would like to collect some details about you and where you live at present	
	Contact details	
4.1	Family name	_____[Surname]
4.2	First name/middle name	_____[Forename/other name]
4.3	Current house address (if any) [House No./ Street / Locality]	_____ _____
4.4	Place name	_____ [Name of Village/Town/City]
4.5	PIN Code	☐☐☐☐☐☐
4.6	Sub-district	_____ [Tehsil/Taluk/Mandal/Municipality]
4.7	District	_____
4.8	Nearest railway station	_____
4.9	Nearest big town	_____ [In case of village only]
4.10	State	_____ [Name of country if abroad]
4.11	Type of place	☐ [1=Village; 2=Town; 3=Small city; 4=Large city]
4.12	Census code	☐☐☐☐☐☐☐☐☐☐
4.13	Home telephone number (landline)	(☐☐☐☐☐) ☐☐☐☐☐☐☐☐ [Area code] [Phone number]
4.14	Mobile number	☐☐☐☐☐☐☐☐☐☐
	Now I would like to collect some personal information about you	

	Personal details	
5.1	Age last birthday	☐☐ [In completed years]
5.2	Day of birth	☐☐ [DD]
5.3	Month of birth	☐☐ [MM]
5.3a.	Season of birth	Summer ☐ Rainy season ☐ Winter ☐
5.4	Year of birth	☐☐☐☐ [YYYY]
5.4a	Birth weight	☐☐☐☐ gms Records-1; Recall-2
5.4b	If you do not know birth weight, try to recall if -	Thin baby ☐ Normal wt ☐ Heavy baby ☐
5.5	Sex	☐ [1=Male; 2=Female]
5.6 a	Sibling History	
	(a) How many brothers (alive) do you have?	☐☐ [Enter 00 if None]
	(b) How many sisters (alive) do you have?	☐☐ [Enter 00 if None]
	(c) What was your birth order in your family?	☐☐
	(d) Do you have a twin brother or sister?	☐ [1=Yes; 2=No]
5.6 b	Ethnicity and religion	
	What is your place of Origin ? _____	
	(b) Which category do you belong to?	☐ 1 – General , 2 – SC , 3 – ST , 4 – OBC , 5 - Others
	(c) What religion do you belong to ?	☐ 1 – Muslim , 2 – Hindu , 3 – Christian , 4 – Others
	(d) In case of Hindu General Category, what caste to you belong to ? ☐<br>1- Brahmin, 2 - Kshatriya, 3-Vaish, 4 - Others	
	(e) In case you are a Muslim which category do you belong to? ☐<br>1- Shia 2 - Sunni 3 - Other	
	(f) If belonging to a Tribe, which one do you belong to? _____	

5.6 c	Consanguinity<br>(a) If married, is your spouse a close relative before marriage? ☐ 1-Yes 2- No<br>(b) If yes, what is the relation? ☐ 1-Sibling, 2- First cousin (paternal/maternal),<br>3. Second cousin (paternal/maternal) 4. Uncle (maternal/paternal)<br>5. Niece (paternal/Maternal) 6. Other	
5.7	Current marital status	☐ 1=Never married<br>2=Married<br>3=Widow/widower<br>4=Separated/divorced
5.8	If ever married:	
	(a) How old were you when you first started living with your spouse after your marriage?	☐☐ [Age in completed years]
	(b) Does your spouse normally live with you now?	☐ [1=Yes; 2=No]
5.9	How many (live) sons do you have?	☐☐ [Enter 00 if None]
5.10	How many (live) daughters do you have?	☐☐ [Enter 00 if None]
5.10a<br><br>5.10b	What was the index child/children's birth order (BO)?<br><br>Please recall whether the child/children (all) received nutritional supplementation from the Anganwadi Yes=1 and No=2<br>i. If yes, at what age.	☐, ☐, ☐, ☐<br>BO b. Supplemented i. Age<br>1. ☐ ☐ _____<br>2. ☐ ☐ _____<br>3 ☐ ☐ _____<br>4. ☐ ☐ _____
5.10c	What was the index child's gender?	☐☐☐☐ [1=Male; 2=Female]
5.10d	Was the child breast-fed?	☐☐☐☐ Yes =1 and No=2
5.10e	What was the duration of ***EXCLUSIVE*** breast-feeding?	☐☐☐☐ months
	Primary occupation	
5.11	(a) Respondent: ☐	(b) Spouse (if married): ☐
	1=At home doing housework<br>2=Unemployed, not seeking work: retired/ disabled<br>3=Unemployed, seeking work	4= Student/ training<br>5=Unskilled manual<br>6=Semi-skilled manual<br>7=Skilled manual
		8=Skilled non-manual<br>9=Semi-Professional<br>10=Professional

5.12	Briefly describe your job: _____	
	Highest educational level attained	
5.13	(a) Respondent: ☐	(b) Spouse (if married) ☐
	1=Illiterate<br>2=Literate, no formal education<br>3=Up to primary school (class IV)	4=Secondary school (ITI course, class X/XII, Intermediate)<br>5=Graduate (BA, BSc, BCom, Diploma)-completed<br>6=Professional degree/postgraduate (MA, MSc, MBBS, MSW, BTech, PhD)
Now I am going to ask you some questions about your household		
	Current household circumstances	
6.1	What kind of household do you currently live in?	☐
	1=Single<br>2=Hostel/shared accommodation<br>3=Nuclear family (married couple & offspring)<br>4=Extended family (2 related married couples of different generations i.e. married couple with one of the parents)	5=Joint family (two related married couples from same generation (i.e. two married siblings)<br>6=Joint-extended<br>7=Any other
6.2	What is the material used in the construction of the house?	☐ 1=Kutcha<br>2=Semi-pucca<br>3=Pucca
6.3 a	What is the main source of lighting for your household?	☐ 1=Electricity<br>2=Kerosene<br>3=Gas<br>4=Oil<br>5=Other
6.3 b	What is the main source cooking fuel?	☐ 1=Electricity<br>2=Kerosene<br>3=Gas<br>4=Oil<br>5=Other
6.4	What is the main source of drinking water for members of your household?	☐ 1=Pipe, hand pump, well (in residence/ plot)<br>2=Pipe, hand pump or well (public)<br>3=Other
6.5	What kind of toilet facility does the household have?	☐ 1=Own flush toilet<br>2=Own pit toilet/latrine<br>3=No facility/field/bush<br>4=Other
6.6	Do you collect rations from a ration card?	☐ [1=Yes; 2=No]
	SKIP QUESTIONS 6.7-6.10 IF LIVING IN HOSTEL/SHARED ACCOMMODATION	
6.7	Including yourself, how many people normally live in your household?	☐☐ [Number of People]

6.8 a	How many rooms are there in your household? (count all rooms including kitchen, bathroom, etc)	☐☐ [Number of Rooms]
6.8 b	Do you have a separate kitchen at home ?	☐ [1=Yes; 2=No]
6.8 c	If yes, for how many years ?	☐☐
6.9	Does this household own any agricultural land?	☐ [1=Yes; 2=No]
6.10	Does the household own any of the following:	
	(a) Clock/Watch	☐ [1=Yes; 2=No]
	(b) Radio/Transistor/Tape recorder	☐ [1=Yes; 2=No]
	(c) Television	☐ [1=Yes; 2=No]
	(d) Bicycle	☐ [1=Yes; 2=No]
	(e) Motorcycle/scooter/moped	☐ [1=Yes; 2=No]
	(f) Car	☐ [1=Yes; 2=No]
	(g) Refrigerator	☐ [1=Yes; 2=No]
	(h) Telephone	☐ [1=Yes; 2=No]
	(i) Water pump	☐ [1=Yes; 2=No]
	(j) Bullock cart	☐ [1=Yes; 2=No]
	(k) Thresher	☐ [1=Yes; 2=No]
	(l) Tractor	☐ [1=Yes; 2=No]

	Now thinking back to when you were a child, say 10-12 years old, please answer the following questions about the household where you lived at that time		
	Household circumstances in childhood (at age 10-12 years)		
7.1	What was your father's occupation at the time? ☐		
	1=At home doing housework	4= Student/ training	8=Skilled non-manual
	2=Unemployed, not seeking work: retired/ disabled	5=Unskilled manual	9=Semi-Professional
		6=Semi-skilled manual	10=Professional
	3=Unemployed, seeking work	7=Skilled manual	11=Died, left family
7.2	What was the highest educational level attained by your mother?		☐

	1=Illiterate<br>2=Literate, no formal education<br>3=Up to primary school (class IV)	4=Secondary school (ITI course, class X/XII, intermediate)<br>5=Graduate (BA, BSc, B.Com, Diploma)<br>6=Professional degree/postgraduate<br>(MA, MSc, MBBS, MSW, B.Tech, PhD)		
7.3	What was the highest educational level attained by your father?	☐		
	1=Illiterate<br>2=Literate, no formal education<br>3=Up to primary school (class IV)	4=Secondary school (ITI course, class X/XII, Intermediate)<br>5=Graduate (BA, BSc, BCom, Diploma)<br>6=Professional degree/postgraduate (MA, MSc, MBBS, MSW, BTech, PhD)		
7.4	What was your mother's occupation at the time	☐		
	1=At home doing housework<br>2=Unemployed, not seeking work: retired/ disabled<br>3=Unemployed, seeking work	4= Student/ training<br>5=Unskilled manual<br>6=Semi-skilled manual<br>7=Skilled manual	8=Skilled non-manual<br>9=Semi-Professional<br>10=Professional<br>11=Died, left family	
7.5	What kind of household did you live in?	☐		
	1=Single<br>2=Hostel/shared accommodation<br>3=Nuclear family (married couple & offspring)<br>4=Extended family (2 related married couples of different generations i.e. married couple with one of the parents)	5=Joint family (two related married couples from same generation (i.e. two married siblings)<br>6=Joint-extended<br>7=Any other		
7.6	What was the material used in the construction of the house?	☐	1=Kutcha<br>2=Semi-pucca<br>3=Pucca	
7.7a	What was the main source of lighting for your household?	☐	1=Electricity<br>2=Kerosene<br>3=Gas	4=Oil<br>5=Other
7.7b	What was the main source cooking fuel?	☐	1=Electricity<br>2=Kerosene<br>3=Gas	4=Oil<br>5=Other

7.8	What was the main source of drinking water for members of your household?	☐ 1=Pipe, hand pump, well (in residence/ plot)<br>2=Pipe, hand pump or well (public)<br>3=Other
7.9	What kind of toilet facility did the household have?	☐ 1=Own flush toilet<br>2=Own pit toilet/latrine<br>3=No facility/field/bush<br>4=Other
7.10	Did you collect rations from a ration card?	☐ [1=Yes; 2=No]

SKIP QUESTIONS 7.11 -7.14 IF LIVING IN HOSTEL/SHARED ACCOMMODATION

7.11	Including yourself, how many people normally lived in your household?	☐☐ [Number of People]
7.12 a	How many rooms were there in your household? (count all rooms including kitchen, bathroom, etc)	☐☐ [Number of Rooms]
7.12 b	Did you have separate kitchen at home?	☐ [1=Yes; 2=No]
7.12 c	If yes , for how many years ?	☐☐
7.13	Did this household own any agricultural land?	☐ [1=Yes; 2=No]
7.14	Did the household own any of the following:	
	(a) Clock/Watch	☐ [1=Yes; 2=No]
	(b) Radio / Transistor /Tape recorder	☐ [1=Yes; 2=No]
	(c) Television	☐ [1=Yes; 2=No]
	(d) Bicycle	☐ [1=Yes; 2=No]
	(e) Motorcycle / scooter / moped	☐ [1=Yes; 2=No]
	(f) Car	☐ [1=Yes; 2=No]
	(g) Refrigerator	☐ [1=Yes; 2=No]
	(h) Telephone	☐ [1=Yes; 2=No]
	(i) Water pump/ motor	☐ [1=Yes; 2=No]
	(j) Bullock cart	☐ [1=Yes; 2=No]
	(k) Thresher	☐ [1=Yes; 2=No]
	(l) Tractor	☐ [1=Yes; 2=No]

Now I will ask you a few questions about your health and lifestyle						
Health and lifestyle						
8.1	(i) Have your ever used tobacco on a REGULAR basis (at least weekly)?	(ii)Age at starting	(iii) Duration of use	(iv) Number of days per week	(v) Number of use or smoked per day	(vi) Time of day when first cigarette is smoked
(a) Smoked	1=Never ☐ 2=Former (stopped >6months) 3=Current (in last 6 months	☐☐ [Yrs]	☐☐ [Yrs]	☐ [Days]	☐☐	☐☐.☐☐ a.m/p.m
(b) Chewed	1=Never ☐ 2=Former (stopped >6months) 3=Current (in last 6 months	☐☐ [Yrs]	☐☐ [Yrs]	☐ [Days]	☐☐	☐☐.☐☐ a.m/p.m
(c) Snuffed	1=Never ☐ 2=Former (stopped >6months) 3=Current (in last 6 months	☐☐ [Yrs]	☐☐ [Yrs]	☐ [Days]	☐☐	☐☐.☐☐ a.m/p.m
8.2	(a) Is there someone in your household who smokes tobacco at home? [If no, skip to 8.3] ☐ [1=Yes; 2=No]					
	If yes, (b) How many cigarettes or beedis does this person smoke per day? ☐☐☐ beedis /cigarettes per day					
8.3	(a) Has an indoor open fire with wood, crop residues or dung been used in your home as a primary means of cooking for more than 6 months in your life? [If no, skip to 8.4] ☐ [1=Yes; 2=No]					
	If yes, (b) For how many years has wood, crop residues or dung been used for cooking in your home? ☐☐ [Years]					
	(c) On average for how many hours a day have you personally spent cooking using wood, crop residues or dung? [00 if none] ☐☐ [Hours]					
	(d) Is wood, crop residues or dung still used for cooking in your home? ☐ [1=Yes; 2=No]					

	(e) Was your stove or fire vented to the outside? ☐ [1=Yes; 2=No]	
8.4	Would you describe your present alcohol intake as?<br>a. Locally made spirits<br>b. Branded spirits<br>c. Beer<br>d. Wine	☐ 1=Daily/most days<br>☐ 2=Weekends only<br>☐ 3= 1-2 times/month<br>☐ 4=Special occasions<br>5=Never
8.4a	Measures or glasses per occasion<br>a. Locally made spirits<br>b. Branded spirits<br>c. Beer<br>d. Wine	No. of glasses ☐☐<br>☐☐<br>☐☐<br>☐☐<br>ml ☐☐☐<br>☐☐☐<br>☐☐☐<br>☐☐☐

	Now I will ask you a few questions about how you have been feeling in general. I will read out a list of statements, please tell me which one best describes your health state today.			
	Quality of life			
9.1	Mobility	☐	1= I have no problems in walking around;<br>2= I have some problems in walking around;<br>3=I am confined to bed	
9.2	Self care	☐	1= I have no problems with washing and dressing myself;<br>2= I have some problems with washing or dressing myself;<br>3=I am unable to wash and dress myself	
9.3	Usual activities	☐	(e.g. work, study, housework, family or leisure activities)<br>1= I have no problems with performing my usual activities;<br>2= I have some problems with performing my usual activities;<br>3=I am unable to perform my usual activities	
9.4	Pain/discomfort	☐	1= I have no pain or discomfort;<br>2= I have moderate pain or discomfort;<br>3=I have extreme pain or discomfort	
9.5	Anxiety/Depression	☐	1= I am not anxious or depressed;<br>2= I am moderately anxious or depressed;<br>3=I am extremely anxious or depressed	
9.6	We have drawn a scale on which the best state you can imagine is marked 100 and the worst state you can imagine is marked 0. Please indicate on this scale how good or bad your own health is today, in your opinion			☐☐☐ %
Depression				
	Over the last 2 weeks, how often have you been bothered by any of the following problems?	1=Not at all<br>2=Several days	3=More than half the days<br>4=Nearly every day	
9.7	Little interest or pleasure in doing things			☐
9.8	Feeling down, depressed, or hopeless			☐
9.9	Trouble falling or staying asleep, or sleeping too much			☐
9.10	Feeling tired or having little energy			☐
9.11	Poor appetite or overeating			☐
9.12	Feeling bad about yourself, or that you are a failure, or have let yourself or your family down			☐
9.13	Trouble concentrating on things, such as reading the newspaper or watching television			☐
9.14	Moving or speaking so slowly that other people could have noticed. Or the opposite – being so fidgety or restless that you have been moving around a lot more than usual			☐
9.15	Thoughts that you would be better off dead, or of hurting yourself in some way			☐

9.16	In the last 4 weeks, have you had an anxiety attack – suddenly feeling fear or panic	☐ [1=Yes; 2=No]
IF NO, SKIP QUESTIONS 9.17-9.20 AND GO TO QUESTION 9.21		
	If yes,	
9.17	Has this happened before?	☐ [1=Yes; 2=No]
9.18	Do some of these attacks come suddenly out of the blue – that is, in situations where you don't expect to be nervous or uncomfortable?	☐ [1=Yes; 2=No]
9.19	Do these attacks bother you a lot or are you worried about having another attack?	☐ [1=Yes; 2=No]
9.20	During your last bad anxiety attack, did you have symptoms like shortness of breath, sweating, your heart racing or pounding, dizziness or faintness, tingling or numbness, or nausea or upset stomach?	☐ [1=Yes; 2=No]
9.21	If you checked off any problems on this questionnaire so far, how difficult have these problems made it for you to do your work, take care of things at home, or get along with other people?	☐ 1=Not difficult at all<br>2=Somewhat difficult<br>3=Very difficult<br>4=Extremely difficult
In the last 4 weeks, how much have you been bothered by any of the following problems?		1=Not bothered<br>2=Bothered a little<br>3=Bothered a lot
9.22	Worrying about your health	☐
9.23	Your weight or how you look	☐
9.24	Difficulties with husband/wife, parents, or other relatives	☐
9.25	The stress of taking care of children, parents or other family members	☐
9.26	Stress at work outside of home or at school	☐
9.27	Financial problems or worries	☐
9.28	Having no one to turn to when you have a problem	☐
9.29	Something bad that happened recently	☐

9.30	Thinking or dreaming about something terrible that had happened to you in the past – like your house being destroyed, a severe accident, being hit or assaulted, or being forced to commit a sexual act	☐

Now I will ask you questions relating to your usual sleep patterns.				
10.1	How many hours do you usually sleep per day (including sleep at night and during the day) on a typical day when you have school or work the next day?	☐☐.☐ [Completed half hours]		
10.2	How many hours do you usually sleep per day (including sleep at night and during the day) on a typical day when you do not have school or work the next day?	☐☐.☐ [Completed half hours]		
10.3	(a) Do you undertake shift work that interrupts your usual sleep patterns?	☐ [1=Yes; 2=No]		
	(b) If yes, how often is the shift work (over the last month)?	☐ 1=Daily<br>2=5-6 times/week	3=2-4 times/week<br>4=Once a week	5=2-3 times/month<br>6=Once a month
10.4	In the past month, how often have you experienced difficulties in getting to sleep?	☐ 1=Daily<br>2=5-6 times/week	3=2-4 times/week<br>4=Once a week	5=2-3 times/month<br>6=Once a month<br>7=Never
10.5	In the past month, how often have you been bothered by awakening during night?	☐ 1=Daily<br>2=5-6 times/week	3=2-4 times/week<br>4=Once a week	5=2-3 times/month<br>6=Once a month<br>7=Never

PHYSICAL ACTIVITY QUESTIONNAIRE

	Now I am going to ask you questions about the time you spent doing different types of physical activity. Please recall the activities that you did in the LAST WEEK.<br>The first questions are about your work/college. This includes paid jobs, working in your farm, study/training, any volunteer work or college activities.<br>Do not include unpaid work you might do around your home, like housework, garden work, and caring for your family. I will ask you about these later.	
	Work related activity	
11.1	Do you currently have a job or do any unpaid work or study/training? Do not include household work, we will ask about this later.	☐ [1=Yes; 2=No]<br>[IF NO, SKIP TO 11.8]
11.2	How many days did you work at the job or unpaid work in the last week?	☐ [In completed days]
11.3	In the last week, how many hours per day did you spend at this work?	☐☐.☐
	Of the hours you spend at work in a day during the last week I am going to ask you how many hours you spend in	

	standing, sitting, walking and other strenuous activities:		
	(a) Standing: E.g. talk, lab work, supervise, mild cleaning, cattle grazing done standing.	(b) Sitting: E.g. typing, computer work, cleaning grains, eating lunch, driving for your work, etc	(c) Walking: E.g. walking around, strolling, walking with light loads
	☐☐.☐ [hours]	☐☐.☐ [hours]	☐☐.☐ [hours]

11.4	If you spend any time at work on activities more strenuous than walking, please list these:			
		(i) Took part in this activity	(ii) Days per week	(iii) Total duration per day
	(a) Carrying/walking with loads (15-25 kg)	☐ [1=Yes; 2=No]	☐ days	☐☐☐ [mts]
	(b) Carrying/walking with heavy load (≥25 kg)	☐ [1=Yes; 2=No]	☐ days	☐☐☐ [mts]
	(c) Lifting / loading of weights	☐ [1=Yes; 2=No]	☐ days	☐☐☐ [mts]
	(d) Pushing cart with a load	☐ [1=Yes; 2=No]	☐ days	☐☐☐ [mts]
	(e) Ploughing	☐ [1=Yes; 2=No]	☐ days	☐☐☐ [mts]
	(f) Digging	☐ [1=Yes; 2=No]	☐ days	☐☐☐ [mts]
	(g) Watering / weeding fields	☐ [1=Yes; 2=No]	☐ days	☐☐☐ [mts]
	(h) Cut / chop wood or stones	☐ [1=Yes; 2=No]	☐ days	☐☐☐ [mts]
	(i) Harvesting	☐ [1=Yes; 2=No]	☐ days	☐☐☐ [mts]
	(j) Any others?	☐ [1=Yes; 2=No]		
	(k) _____		☐ days	☐☐☐ [mts]
	(l) _____		☐ days	☐☐☐ [mts]
	(m)_____		☐ days	☐☐☐ [mts]

	Travel to and from work<br>Now think about how you travelled to and from work over the LAST WEEK. Please do not include travelling activities if you have already mentioned while we discussed your work/college activities.		
		(a) Days per week	(b) Total duration per day
11.5	During the last week, how many days did you travel on a motorised vehicle, like a car, bus, auto-rickshaw or motorcycle to and from work?	☐ days	☐☐☐ [mts]
11.6	During the last week, on how many days did you cycle to and from work?	☐ days	☐☐☐ [mts]

11.7	During the last week, on how many days did you walk to and from work?	☐ days		☐☐☐ [mts]
	Travel apart from to and from work<br>Now think about how you travelled from place to place over the LAST WEEK, including places like stores, movies, visiting relatives etc but excluding to and from work. Please do not include travelling activities if you have already mentioned.			
		(a) Days per week		(b) Total duration per day
11.8	During the last week, how many days did you travel to places on a motorised vehicle, like a car, bus, auto-rickshaw or motorcycle except to and from work?	☐ days		☐☐☐ [mts]
11.9	During the last week, on how many days did you travel to places on a bicycle except to and from work?	☐ days		☐☐☐ [mts]
11.10	During the last week, on how many days did you travel to places by walking except to and from work ?	☐ days		☐☐☐ [mts]
	Now I am going to ask you some questions about how you spent your time, apart from work outside of the home over the LAST WEEK			
11.11	Sports / games / exercise<br>Now think about all the physical activities that you did in the last 7 days solely for sport, exercise of leisure. Please do not include any activities you have already mentioned.			
	Name of activity	(i) Took part in this activity	(ii) Days per week	(iii) Total duration per day
	(a) Walking normal speed for leisure	☐ [1=Yes; 2=No]	☐ days	☐☐☐ [mts]
	(b) Walking brisk speed for leisure	☐ [1=Yes; 2=No]	☐ days	☐☐☐ [mts]
	(c) Jogging/Running	☐ [1=Yes; 2=No]	☐ days	☐☐☐ [mts]
	(d) Badminton	☐ [1=Yes; 2=No]	☐ days	☐☐☐ [mts]
	(e) Cricket	☐ [1=Yes; 2=No]	☐ days	☐☐☐ [mts]
	(f) Yoga	☐ [1=Yes; 2=No]	☐ days	☐☐☐ [mts]
	(g) Swimming	☐ [1=Yes; 2=No]	☐ days	☐☐☐ [mts]
	(I) Volleyball	☐ [1=Yes; 2=No]	☐ days	☐☐☐ [mts]
	(j) Kabbadi	☐ [1=Yes; 2=No]	☐ days	☐☐☐ [mts]
	(k) Cycling	☐ [1=Yes; 2=No]	☐ days	☐☐☐ [mts]

	(m) Any others?	☐ [1=Yes; 2=No]		
	(n)		☐ days	☐☐☐ [mts]
	(o)		☐ days	☐☐☐ [mts]
	(p)		☐ days	☐☐☐ [mts]
11.12	Household activities<br>Now think about activities you do at home such as housework, gardening and hobbies. Please do not include any activities already mentioned.			
	Name of activity	(i) Took part in this activity	(ii) Days per week	(iii) Total duration per day
	(a) Cooking	☐ [1=Yes; 2=No]	☐ days	☐☐☐ [mts]
	(b) Washing vessels	☐ [1=Yes; 2=No]	☐ days	☐☐☐ [mts]
	(c) Mopping	☐ [1=Yes; 2=No]	☐ days	☐☐☐ [mts]
	(d) Sweeping	☐ [1=Yes; 2=No]	☐ days	☐☐☐ [mts]
	(e) Wash clothes manually	☐ [1=Yes; 2=No]	☐ days	☐☐☐ [mts]
	(f) Dusting / cleaning	☐ [1=Yes; 2=No]	☐ days	☐☐☐ [mts]
	(g) Ironing and folding clothes	☐ [1=Yes; 2=No]	☐ days	☐☐☐ [mts]
	(h) Child care	☐ [1=Yes; 2=No]	☐ days	☐☐☐ [mts]
	(i) Collecting fuel/fodder/water	☐ [1=Yes; 2=No]	☐ days	☐☐☐ [mts]
	(j) Animal care	☐ [1=Yes; 2=No]	☐ days	☐☐☐ [mts]
	(k) Gardening	☐ [1=Yes; 2=No]	☐ days	☐☐☐ [mts]
	(l) Any others?	☐ [1=Yes; 2=No]		
	(m)Washing clothes by machine	☐ [1=Yes; 2=No]	☐ days	☐☐☐ [mts]
	(n)_____		☐ days	☐☐☐ [mts]
	(o)_____		☐ days	☐☐☐ [mts]

11.13	Sedentary activities<br>The last question is about time spent sitting in the last 7 days. Do not include time spent sitting at work Please do not include any activities already mentioned.			
	Name of activity	(i) Took part in this activity	(ii) Days per week	(iii) Total duration per day
	(a) Reading for leisure	☐ [1=Yes; 2=No]	☐ days	☐☐☐ [mts]
	(b) Computer /computer games / internet for leisure	☐ [1=Yes; 2=No]	☐ days	☐☐☐ [mts]
	(c) Watching TV/ movies	☐ [1=Yes; 2=No]	☐ days	☐☐☐ [mts]
	(d) Indoor games (e.g. chess, carom, playing cards)	☐ [1=Yes; 2=No]	☐ days	☐☐☐ [mts]
	(e) Prayer/meditation	☐ [1=Yes; 2=No]	☐ days	☐☐☐ [mts]
	(f) Listening to music/radio	☐ [1=Yes; 2=No]	☐ days	☐☐☐ [mts]
	(g) Sewing /embroidery/ knitting	☐ [1=Yes; 2=No]	☐ days	☐☐☐ [mts]
	(h) Socialising (talking outside working hours or on phone)	☐ [1=Yes; 2=No]	☐ days	☐☐☐ [mts]
	(i) Any others?	☐ [1=Yes; 2=No]		
	(j) Sitting idle	☐ [1=Yes; 2=No]	☐ days	☐☐☐ [mts]
	(k)_____		☐ days	☐☐☐ [mts]
11.14	Routine activities			
	Name of activity	(i) Took part in this activity	(ii) Days per week	(iii) Total duration per day
	(a) Eating (breakfast, lunch ,dinner)	☐ [1=Yes; 2=No]	☐ days	☐☐☐ [mts]
	(b) Brushing, shaving, bathing	☐ [1=Yes; 2=No]	☐ days	☐☐☐ [mts]
	(c) Dressing	☐ [1=Yes; 2=No]	☐ days	☐☐☐ [mts]

FOOD FREQUENCY QUESTIONNAIRE

INSTRUCTION TO SUBJECT: I am now going to ask you about the food that you have eaten over the last year. If you have not heard of an item please answer "No".							
	CEREALS	Portion Size	(a) Average consumption	(b) Per Day[1]	(b) Per Week[2]	(b) Per Month[3]	(b) Per Year/ Never[4]
12.1	Chapathis / roti	No					
12.2	Parathas / naan	No					
12.3	Jowar roti	No					
12.4	Poori, bhatura	No					
12.5	Plain rice	Bowl					
12.6	Mutton, chicken biriyani	Bowl					
12.7	Lime rice, puliogare, veg biriyani	Bowl					
12.8	Bhagar	Bowl					
12.9	Upma	Bowl					
12.10	Idlis	No					
12.11	Dosa / uthappam	No					
12.12	Pesarattu	No					
12.13	Attakalu	Bowl					
12.14	Rice, ragi porridge	Bowl					
12.15	Corn flakes	Bowl					
12.16	Bread, Toast, Rolls, Buns	No					
12.17	Noodles, pasta etc	Bowl					
	LENTILS / DHALS / GRAVIES						
13.1	Plain dhal sambar	Ladle					
13.2	Dhal sambar with vegetables	Ladle					
13.3	Channa, rajma, dry peas etc. curry	Ladle					
13.4	Green leafy vegetable curry	Ladle					
13.5	Rasam, all types	Ladle					

		Portion Size	(a) Average consumption	(b) Per Day[1]	(b) Per Week[2]	(b) Per Month[3]	(b) Per Year/ Never[4]
	CHUTNEYS / SALAD / PAPAD						
14.1	Raw vegetable salad	Tbsp					
14.2	Vegetable Raitha	Tbsp					
14.3	Pickle	Tsp					
14.4	Papad	No					
14.5	Coconut chutney	Tbsp					
14.6	Groundnut chutney	Tbsp					
14.7	Tomato chutney	Tbsp					
	NON – VEGETARIAN						
15.1	Chicken curry	Bowl					
15.2	Chicken fry/grilled	No					
15.3	Mutton/ pork/beef curry or fry	Bowl					
15.4	Fish curry	Bowl					
15.5	Fish fry	No					
15.6	Organ meats (Liver, brain, kidney etc.)	Tbsp					
15.7	Prawn, crab, shell fish etc.	Bowl					
15.8	Egg (boiled, poached, omelettes)	No					
	MILK & BEVERAGES						
16.1a.	Tea –with milk	Glass					
16.1b.	Tea –without milk	Glass					
16.2 a.	Coffee – with milk	Glass					
16.2 b.	Coffee-without milk	Glass					
16.3	Plain milk	Glass					
16.4	Flavored milk (horlicks, bournvita etc)	Glass					
16.5	Curds	Bowl					
16.6	Buttermilk/Lassi	Glass					
16.7	Lime/ orange/ other fresh fruit juice	Glass					

		Portion Size	(a) Average consumption	(b) Per Day[1]	(b) Per Week[2]	(b) Per Month[3]	(b) Per Year/ Never[4]
16.8	Fanta, pepsi, coca cola etc.	250ml bottle					
16.9	Beer	Bottle					
16.10	Spirits (whiskey, gin, rum, arrack)	30ml peg					
16.11	Other local alcoholic drinks	30ml peg					
	MISCELLANEOUS						
17.1	Ghee/ butter	Tsp					
17.2	Jam	Tsp					
17.3	Sugar	Tsp					
17.4	Salt	Tsp					
	SNACKS/ SWEETS/DESSERTS						
18.1	Mixture, namkeen, chiwda, khara boondi, dalmoth	Tbsp					
18.2	Vada, all types	No					
18.3	Nuts (groundnuts, cashewnuts etc.)	Tbsp					
18.4	Chips/salted packed snacks (bingo, kurkure etc)	Bowl					
18.5	Samosa,bajji ,bonda, cutlet, patties	No					
18.6	Salted biscuits (krackjack, bakery biscuits)	No					
18.7	Sweet biscuits (Marie/good day/cream biscuits)	No					
18.8	Murukku , chakli, sakinalu	No					
18.9	Cakes or sweet pastries	No					
18.10	Payasam, kheer	Bowl					
18.11	Ice cream	Bowl					

		Portion Size	(a) Average consumption	(b) Per Day[1]	(b) Per Week[2]	(b) Per Month[3]	(b) Per Year/ Never[4]
18.12	Jamoon, Jilebi, Jangir etc.	No					
18.13	Mysore pak, ladoo, barfis	No					
18.14	Baksham	No					
18.15	Dried fruits (dates, figs, raisins etc)	Tbsp					
18.16	Chocolates	Small Bar					

	FRUITS	Portion size	(a) Average consumption	(b) Per Day[1]	(b) Per Week[2]	(b) Per Month[3]	(b) Per Year/ Never[4]	(c) Seasonal (cross if seasonal)
19.1	Banana	No						
19.2	Apple	No						
19.3	Orange	No						
19.4	Mango	No						
19.5	Guava (amrood)	No						
19.6	Grapes (angoor)	Bowl						
19.7	Pineapple	Slice						
19.8	Papaya (papita)	Slice						
19.9	Pomegranate (anar)	No						
19.10	Sapota (Chikoo)	No						
19.11	Watermelon(tarbooj)	Bowl						
19.12	Musk melon (kharbooj)	Bowl						
19.13	Custard apple	No						
19.14	Zizyphus (ber)	No						
19.15	Sugarcane (ganaa)	Pieces						
19.16	Palmyra	No						
	VEGETABLES							
20.1	Palak, methi, other leafy vegetables	Tbsp						
20.2	Potato, sweet potato	Tbsp						

20.3	Beetroot/ radish	Tbsp						
20.4	Cabbage	Tbsp						
20.5	Beans, cluster beans	Tbsp						
		Portion size	(a) Average consumption	(b) Per Day[1]	(b) Per Week[2]	(b) Per Month[3]	(b) Per Year/ Never[4]	(c) Seasonal (cross if seasonal)
20.6	Ladies finger	Tbsp						
20.7	Cauliflower	Tbsp						
20.8	Bottlegourd (lauki), ashgourd, Ridgegourd (turai), snakegourds, etc.	Tbsp						
20.9	Brinjal	Tbsp						
20.10	Kovai	Tbsp						
20.11	Capsicum/ green pepper	Tbsp						
20.12	Drumstick	Pieces						
20.13	Raw plantain	Tbsp						
20.14	Colacasia (arvi)	Tbsp						

21.1	Which type of oil is consumed most by your family? State in order of decreasing quantity of use.	☐<br>☐<br>☐<br>☐<br>☐<br>☐	Quantity in Kg/month<br>1=Sunflower oil ☐☐.☐☐<br>2=Groundnut oil ☐☐.☐☐<br>3=Coconut oil ☐☐.☐☐<br>4=Palm oil ☐☐.☐☐<br>5=Mustard oil ☐☐.☐☐<br>6=Dalda /vanaspathi ☐☐.☐☐	7=Butter ☐☐.☐☐<br>8=Ghee ☐☐.☐☐<br>9=Olive oil ☐☐.☐☐<br>10=Corn ☐☐.☐☐<br>11=Rice bran oil ☐☐.☐☐<br>12=Soya bean oil ☐☐.☐☐<br>13=Cotton seed oil ☐☐.☐☐
21.2	How many coconuts do you use for cooking in a month?	☐☐ [No / month] [00 if none]		
21.3	(a) What type of milk do you regularly consume?	☐	1=Whole milk	4=Skimmed milk powder

			2=Skimmed milk<br>3=Toned milk	5=Whole and toned milk<br>6=Other
	(b) If other, then specify_____			
21.4	(a) Do you consume any vitamin or mineral supplement once a week? ☐ [1=Yes; 2=No] at least			
	If Yes, (b) Brand name / Type		(c) Dosage(mg)	(d) No. / week
21.5	Are you vegetarian?		☐ [1=Yes; 2=No]	
21.6	Are you on any of the following special diets?			
	(a) Diabetic diet		☐ [1=Yes; 2=No]	
	(b) Low fat diet		☐ [1=Yes; 2=No]	
	(c) High fiber diet		☐ [1=Yes; 2=No]	
	(d) Low salt diet		☐ [1=Yes; 2=No]	
	(e) Weight reducing diet		☐ [1=Yes; 2=No]	
	(f) Other		☐ [1=Yes; 2=No]	
	(g) If other, please specify		1.<br>2.	
	If yes,<br>(h) Since how many years are you on this special diet?		1. ☐☐ [completed years]<br>2. ☐☐ [completed years]	

Now I am going to ask you questions about your family history of illness, and your medical history				
	Medical history			
22.16	Is your father still alive?	(a) ☐ [1=Yes; 2=No]	(b) If no, his age at death ☐☐ [years]	
22.17	(a) If no, what was the cause of his death?	☐ 1=Heart disease<br>2=High blood Pressure<br>3=stroke	4=lung<br>5=cancer<br>6=accident /injury	7=other<br>8=don't know
	(b) If "other" specify:			

	Did/does your father suffer from any of the following?		
22.18	Diabetes	☐ [1=Yes; 2=No; 3=Don't know]	
22.19	High blood pressure	☐ [1=Yes; 2=No; 3=Don't know]	
22.19a.	Stroke	☐ [1=Yes; 2=No; 3=Don't know]	
22.20	Heart disease	☐ [1=Yes; 2=No; 3=Don't know]	
22.21	Overweight / obesity	☐ [1=Yes; 2=No; 3=Don't know]	
22.22	Lung disease	☐ [1=Yes; 2=No; 3=Don't know]	
22.23	Is your mother still alive?	(a) ☐ [1=Yes; 2=No]	(b) If no, her age at death ☐☐ [years]
22.24	(a) If no, what was the cause of her death?	☐ 1=Heart disease; 2=High blood Pressure; 3=stroke; 4=lung; 5=cancer; 6=accident /injury; 7=other; 8=don't know	
	(b) If "other" specify:		
	Did/does your mother suffer from any of the following?		
22.25	Diabetes	☐ [1=Yes; 2=No; 3=Don't know]	
22.26	High blood pressure	☐ [1=Yes; 2=No; 3=Don't know]	
22.26a	Stroke	☐ [1=Yes; 2=No; 3=Don't know]	
22.27	Heart disease	☐ [1=Yes; 2=No; 3=Don't know]	
22.28	Overweight/obesity	☐ [1=Yes; 2=No; 3=Don't know]	
22.29	Lung disease	☐ [1=Yes; 2=No; 3=Don't know]	
	Did/do any of your brothers or sisters suffer from any of the following?		
22.30	Diabetes	☐ [1=Yes; 2=No; 3=Don't know; 4=no siblings]	
22.31	High blood pressure	☐ [1=Yes; 2=No; 3=Don't know; 4=no siblings]	
22.31a	Stroke	☐ [1=Yes; 2=No; 3=Don't know]	
22.32	Heart disease	☐ [1=Yes; 2=No; 3=Don't know; 4=no siblings]	
22.33	Overweight/obesity	☐ [1=Yes; 2=No; 3=Don't know; 4=no siblings]	
22.34	Lung disease	☐ [1=Yes; 2=No; 3=Don't know; 4=no siblings]	
23.1	(a) Have you had wheezing or whistling in your chest at any time in the last year?	☐ [1=Yes; 2=No]	
	If yes,<br>(b) In the last year have you had this wheezing or whistling only when you have a cold?	☐ [1=Yes; 2=No]	
	(c) In the last year have you ever had an attack of wheezing or whistling that has made you feel short of breath?	☐ [1=Yes; 2=No]	

24.1	(a) Do you usually cough when you don't have a cold? [If no, skip to 24.2]	☐ [1=Yes; 2=No]
	If yes<br>(b) Are there months when you cough most days	☐ [1=Yes; 2=No]
	(c) Do you have a cough on most days for as much as three months each year?	☐ [1=Yes; 2=No]
	(d) For how many years have you had this cough?	☐☐ [Years]
24.2	(a) Do you usually bring up phlegm from your chest, or do you usually have phlegm in your chest that is difficult to bring up when you don't have a cold? [If no, skip to 24.3]	☐ [1=Yes; 2=No]
	If yes,<br>(b) Are there months in which you have this phlegm on most days?	☐ [1=Yes; 2=No]
	(c) Do you bring up this phlegm on most days for as much as 3 months per year?	☐ [1=Yes; 2=No]
	(d) For how many years have you had this phlegm?	☐☐ [Years]
24.3	(a) Are you unable to walk due to a condition other than shortness of breath? [If no, skip to 24.4]	☐ [1=Yes; 2=No]
	(b) If yes, name of condition_____	
24.4	If able to walk: (a) Are you troubled by shortness of breath when hurrying on the level or walking up a slight hill? [If no, skip to 24.5]	☐ [1=Yes; 2=No]
	If yes,<br>(b) Do have to walk slower than most people of your age on level ground because of shortness of breath?	☐ [1=Yes; 2=No]
	(c) Do you have to stop for breath when walking at your own pace on level ground?	☐ [1=Yes; 2=No]
	(d) Do you ever have to stop for breath after walking about 100 yards on level ground?	☐ [1=Yes; 2=No]
	(e) Are you too short of breath to leave the house or short of breath on dressing of undressing?	☐ [1=Yes; 2=No]
24.5	(a) Have you ever had any pain or discomfort in your chest? [If no, end section]	☐ [1=Yes; 2=No]
	If yes,<br>(b) Do you get it when you walk uphill or hurry?	☐ [1=Yes; 2=No]
	(c) Do you get it when you walk at an ordinary pace on the level?	☐ [1=Yes; 2=No]
	If no pain on walking, end section. Otherwise ask d-g	
	(d) What do you do if you get it while you are walking?	☐ 1=Stop/slow down<br>2=Carry on
	(e) If you are standing still, what happens to it?	☐ 1=Relieved<br>2=Not relieved
	(f) How soon?	☐ 1=10 minutes or less<br>2=Over 10 minutes
	(g) Will you show me where it is (record all places)? [SHOW PICTURE]	☐,☐,☐,☐

SECTION D: Anthropometry

	Weight and height	a) First reading	b) Second reading
25.1	Weight	☐☐☐.☐ [kg]	☐☐☐.☐ [kg]
25.2	Scale number	☐	
25.3	Standing height	☐☐☐☐ [mm]	☐☐☐☐ [mm]
25.4	Sitting height	☐☐☐☐ [mm]	☐☐☐☐ [mm]
25.5	Stool height	☐☐☐☐ [mm]	☐☐☐☐ [mm]
25.6	Stadiometer number	☐	
	Circumferences	a) First reading	b) Second reading
25.7	Waist circumference	☐☐☐☐ [mm]	☐☐☐☐ [mm]
25.8	Hip circumference	☐☐☐☐ [mm]	☐☐☐☐ [mm]
25.9	Mid-arm circumference	☐☐☐☐ [mm]	☐☐☐☐ [mm]
25.10	Calf circumference	☐☐☐☐ [mm]	☐☐☐☐ [mm]
25.11	Head circumference	☐☐☐☐ [mm]	☐☐☐☐ [mm]
25.11- i	Chest Circumference at end-inspiration	☐☐☐☐ [mm]	☐☐☐☐ [mm]
25.11- ii	Chest Circumference at end- expiration	☐☐☐☐ [mm]	☐☐☐☐ [mm]

	Skinfold measurements	a) First reading	b) Second reading	c) Third reading
25.12	Triceps skinfold	☐☐.☐ [mm]	☐☐.☐ [mm]	☐☐.☐ [mm]
25.13	Biceps skinfold	☐☐.☐ [mm]	☐☐.☐ [mm]	☐☐.☐ [mm]
25.14	Subscapular skinfold	☐☐.☐ [mm]	☐☐.☐ [mm]	☐☐.☐ [mm]
25.15	Suprailiac skinfold	☐☐.☐ [mm]	☐☐.☐ [mm]	☐☐.☐ [mm]
25.16	Calf skinfold	☐☐.☐ [mm]	☐☐.☐ [mm]	☐☐.☐ [mm]
25.17	Caliper number	☐		

	Muscle strength	Reading 1	Reading 2	Reading 3	Reading 4
25.18	Right hand	☐☐.☐ [kg]	☐☐.☐ [kg]	☐☐.☐ [kg]	☐☐.☐ [kg]
25.19	Left hand	☐☐.☐ [kg]	☐☐.☐ [kg]	☐☐.☐ [kg]	☐☐.☐ [kg]
25.20	Dominant hand	☐ [1=Right, 2=Left]			
25.21	Grip strength machine	☐			

	General information: anthropometry measurements	
25.22	Researcher code	☐☐
25.23	Researcher initials	☐☐☐
25.24	Left sided measurements	☐ [1=Yes; 2=No]
25.25	If not, specify	_____
25.26	All measurements adequate	☐ [1=Yes; 2=No]
25.27	If not, specify	_____

	Blood pressure			
26.1	Room temperature	☐☐ . ☐ [degree Celsius]		
		a) First measure	b) Second measure	b) Third measure
26.2	Systolic BP (brachial)	☐☐☐ [mmHg]	☐☐☐ [mmHg]	☐☐☐ [mmHg]
26.3	Diastolic BP (brachial)	☐☐☐ [mmHg]	☐☐☐ [mmHg]	☐☐☐ [mmHg]
26.4	Pulse rate	☐☐☐ [bpm]	☐☐☐ [bpm]	☐☐☐ [bpm]
26.5	Cuff size used	☐ [1=Small; 2=Medium; 3=Large]		
26.6	BP apparatus number	☐		
26.7	Right arm measurements	☐ [1=Yes; 2=No]		
26.8	Measurements adequate	☐ [1=Yes; 2=No]		
26.9	If not, specify	_____		
	Respiratory function			

27.1a	In the past three months have you had any surgery on your chest or abdomen?	☐ [1=Yes; 2=No]
27.1b	Have you had a hernia problem at any time?	☐ [1=Yes; 2=No]
27.2	Have you had a heart attack within the past three months?	☐ [1=Yes; 2=No]
27.3	Do you have a detached retina or have you had eye surgery within the past three months?	☐ [1=Yes; 2=No]
27.4	Have you been hospitalized with any other heart problem within the past month?	☐ [1=Yes; 2=No]
27.5	Are you in the last trimester of pregnancy?	☐ [1=Yes; 2=No]
27.6	Are you currently taking medication for TB?	☐ [1=Yes; 2=No]
27.7	Have you coughed up blood within the past month?	☐ [1=Yes; 2=No]
27.8	Does the participant have a resting pulse of greater than 120 beats per minute?	☐ [1=Yes; 2=No]
	If any of the questions 27.1 to 27.8 is "yes", do NOT proceed with the test	
27.9	(a) Have you taken medication for breathing in last 6 hours?	☐ [1=Yes; 2=No]
	If yes, name of medication: _____	
27.10	Have you had a respiratory infection (cold) in the last three weeks?	☐ [1=Yes; 2=No]
	TAKE VERBAL CONSENT TO DO THE TEST	

		Pred. value	a) Blow 1	b) Blow 2	c) Blow 3	d) Blow 4	e) Blow 5
27.11	FEV_1		☐.☐☐ [l]	☐.☐☐ [l]	☐.☐☐ [l]	☐.☐☐ [l]	☐.☐☐ [l]
27.12	FVC		☐.☐☐ [l]	☐.☐☐ [l]	☐.☐☐ [l]	☐.☐☐ [l]	☐.☐☐ [l]

27.13	If unable to obtain satisfactory spirometry (check one):	☐ 1 = Participant did not understand instructions<br>2 = Participant medically excluded<br>3 = Participant unable to physically cooperate<br>4 = Participant refused

ONLY FOR WOMEN

	Now I will ask you a few questions about your reproductive history (women only)	
	Reproductive history	
28.1	At what age did your periods start?	☐☐ [Age in completed years]
28.2	(a) Do you still menstruate?	☐ [1=Yes; 2=No]
	(b) If no, at what age did your periods stop?	☐☐ [Age in completed years]
	(c) If yes, do you have irregular/infrequent menstrual cycles?	☐ [1=Yes; 2=No]
	(d) If yes, how many periods do you have in a year?	☐☐ [Number]
28.3	Do you have excess hair growth on your upper lip, chin, lower abdomen or inner thighs?	☐ [1=Yes; 2=No]
28.4	(a) Have you ever taken the oral contraceptive pill?	☐ [1=Yes; 2=No]
	(b) If yes, Which type of pill did you take	☐☐ 1=Combined pill<br>2=Progestogen only (mini pill)<br>3=Don't know
	(c) If yes, for how long did you take it?	☐☐ [Completed years]
28.5	(a) Have you ever been pregnant?	☐ [1=Yes; 2=No]
	(b) If yes, at what age was your first pregnancy?	☐☐ [Age in completed years]
	(c) If yes, how many pregnancies have you had?	☐☐ [Total number, 00 if none]
	(d) If yes, how many live births have you had?	☐☐ [Total number, 00 if none]
	(e) If yes, how many miscarriages/stillbirths have you had?	☐☐ [Total number, 00 if none]
	(f) If yes, how many induced abortions have you had?	☐☐ [Total number, 00 if none]
		[Check that c = d + e + f]
28.6	Have you ever tried to become pregnant during a period of one year or more without success?	☐ [1=Yes; 2=No]
28.7	(a) Are you pregnant at the moment?(Ask if relevant to the sibling)	☐ [1=Yes; 2=No]
	(b) If yes, which trimester of pregnancy are you in?	☐ [1, 2 or 3]
3.2b.	Time of quest. Completion	☐☐ : ☐☐ [Hours: minutes; 24-hour clock]

SECTION E: DXA Measurements

	DXA Scan	
29.1	DXA machine	☐ [1=New; 2=Old]
29.2	Researcher initials	☐☐☐
29.3	Whole scan taken	☐ [1=Yes; 2=No]
29.4	Spine scan taken	☐ [1=Yes; 2=No]
29.5	Hip scan taken	☐ [1=Yes; 2=No]
29.6	If not, specify reason	_____
29.7	First L1-L4 measure taken	☐ [1=Yes; 2=No]
29.8	Second L1-L4 measure taken	☐ [1=Yes; 2=No]
29.9	First L2-L4 measure taken	☐ [1=Yes; 2=No]
29.10	Second L2-L4 measure taken	☐ [1=Yes; 2=No]
29.11	If not, specify reason	_____

SECTION F: Coronary Measures and Medical History

	**Medical history**.		
**30.1**	(a) Have you been diagnosed with any of the following conditions?		(b) *If yes*, age when diagnosed
**30.2**	High blood pressure	(a) ☐ [1=Yes; 2=No]	(b) ☐☐ [Age in completed years]
	(c) Are you on regular medication for your high blood pressure?		☐ [1=Yes; 2=No]
	(d) Name of medicine:	_____	
	(e) Who diagnosed condition	☐ [1=allopathic doctor; 2=homeopath; 3=ayurvedic doctor 4=RMP – registered medical practitioner; 5=Other]	
**30.3**	Diabetes (high blood sugar)	(a) ☐ [1=Yes; 2=No]	(b) ☐☐ [Age in completed years]
	(c) Are you on a regular diet for your diabetes?		☐ [1=Yes; 2=No]
	(d) Are you on regular tablets for your diabetes?		☐ [1=Yes; 2=No]
	(e) Name of medicine:	_____	
	(f) Are you on a regular treatment with insulin?		☐ [1=Yes; 2=No]
	(g) Do you attend a hospital or GP diabetic clinic?		☐ [1=Yes; 2=No]
	(h) Who diagnosed condition	☐ [1=allopathic doctor; 2=homeopath; 3=ayurvedic doctor 4=RMP – registered medical practitioner; 5=Other]	
**30.4**	Heart disease	(a) ☐ [1=Yes; 2=No]	(b) ☐☐ [Age in completed years]
	(c) Are you on regular medication for your heart disease?		☐ [1=Yes; 2=No]
	(d) Name of medicine:	_____	
	(e) Who diagnosed condition	☐ [1=allopathic doctor; 2=homeopath; 3=ayurvedic doctor 4=RMP – registered medical practitioner; 5=Other]	
	(f) Type of heart disease	☐ [1=angina; 2=heart attack; 3=heart failure 4=don't know; 5=Other]	
**30.5**	Stroke (paralytic attack)	(a) ☐ [1=Yes; 2=No]	(b) ☐☐ [Age in completed years]
	(c) Who diagnosed condition	☐ [1=allopathic doctor; 2=homeopath; 3=ayurvedic doctor 4=RMP – registered medical practitioner; 5=Other]	
**30.6**	Asthma, asthmatic bronchitis or allergic bronchitis?	(a) ☐ [1=Yes; 2=No]	(b) ☐☐ [Age in completed years]
	(c) Have you had an attack of asthma in the last year?		☐ [1=Yes; 2=No]
	(d) *If you have asthma,* are you on regular medication for asthma? (tablets/inhaler)		☐ [1=Yes; 2=No]
	(e) Name of medicine:	_____	

**30.7**	Thyroid problem	(a) ☐ [1=Yes; 2=No]	(b) ☐☐ [Age in completed years]
	(c) Are you on regular medication for your thyroid problem?		☐ [1=Yes; 2=No]
	(d) Name of medicine:	_____	
**30.8**	Tuberculosis	(a) ☐ [1=Yes; 2=No]	(b) ☐☐ [Age in completed years]
	(c) Are you on regular medication for your tuberculosis?		☐ [1=Yes; 2=No]
	(d) Name of medicine:	_____	
**30.9**	Depression	(a) ☐ [1=Yes; 2=No]	(b) ☐☐ [Age in completed years]
	(c) Are you on regular medication for your depression?		☐ [1=Yes; 2=No]
	(d) Name of medicine:	_____	
**30.10**	Peptic ulcer	(a) ☐ [1=Yes; 2=No]	(b) ☐☐ [Age in completed years]
**30.11**	COPD	(a) ☐ [1=Yes; 2=No]	(b) ☐☐ [Age in completed years]
**30.12**	Emphysema	(a) ☐ [1=Yes; 2=No]	(b) ☐☐ [Age in completed years]
**30.13**	Chronic bronchitis	(a) ☐ [1=Yes; 2=No]	(b) ☐☐ [Age in completed years]
**30.14**	Cancer	(a) ☐ [1=Yes; 2=No]	(b) ☐☐ [Age in completed years]
	(c) *If yes,* what type of cancer:	_____	

MEDICAL EXAMINATION

	**Carotid IMT**	**(a) Far wall**	**(b) Near wall**
**31.1**	Right common carotid artery image taken	☐ [1=Yes; 2=No]	☐ [1=Yes; 2=No]
**31.2**	Any problems taking images	☐ [1=Yes; 2=No]	☐ [1=Yes; 2=No]
**31.3**	If yes, specify reason	_____	_____

	**Pulse Wave Velocity**			
**32.1**	Room temperature	☐☐ . ☐ [degree Celsius]		
**32.2**	Have you had a meal in last 2 hours?	☐ [1=Yes; 2=No]		
**32.3**	Proximal distance (carotid to notch)	☐☐ . ☐ [cm]		
**32.4**	Distal distance (notch to upper thigh)	☐☐ . ☐ [cm]		
		**(a) First measure**	**(b) Second measure**	**(c) Third measure**
**32.5**	Systolic BP (supine)	☐☐☐ [mmHg]	☐☐☐ [mmHg]	☐☐☐ [mmHg]
**32.6**	Diastolic BP (supine)	☐☐☐ [mmHg]	☐☐☐ [mmHg]	☐☐☐ [mmHg]
**32.7**	Pulse rate (supine)	☐☐☐ [bpm]	☐☐☐ [bpm]	☐☐☐ [bpm]
**32.8**	Pulse wave velocity	☐☐.☐ [m/s]	☐☐.☐ [m/s]	☐☐.☐ [m/s]
**32.9**	Transit time	☐☐☐ [ms]	☐☐☐ [ms]	☐☐☐ [ms]
	**Pulse Wave Analysis**			
**32.10**	Distance (brachial to femoral)		☐☐ . ☐ [cm]	
		**(a) First measure**	**(b) Second measure**	**(c) Third measure**
**32.11**	Augmentation index (Aix)	(☐) ☐☐%	(☐) ☐☐%	(☐) ☐☐%
**32.12**	Central SBP	☐☐☐ [mmHg]	☐☐☐ [mmHg]	☐☐☐ [mmHg]
**32.13**	Central DBP	☐☐☐ [mmHg]	☐☐☐ [mmHg]	☐☐☐ [mmHg]
**32.14**	Heart rate	☐☐☐ [bpm]	☐☐☐ [bpm]	☐☐☐ [bpm]

Section G: TANITA measures

37. 1 Height ☐☐☐ **cm** **37.2 Weight** ☐☐☐ . ☐ **kg**

37.3 BMI ☐☐.☐ **37.4 BMR** ☐☐☐☐ kJ

☐☐☐☐ **Kcal**

**37.5 Total Body Fat**	**37.6 Impedance**
i. Fat Percentage ☐☐. ☐☐ %	i. Whole body ☐☐☐ Ω
ii. Fat mass ☐☐. ☐☐ Kg	ii. Right leg ☐☐☐ Ω
iii. Fat free mass ☐☐ . ☐☐ Kg	iii. Left leg ☐☐☐ Ω
iv. Total body water ☐☐. ☐☐ Kg	iv. Right arm ☐☐☐ Ω
	v. Left arm ☐☐☐ Ω

Segmental Analysis

**38. 7 Right Leg**	**37.8 Left Leg**
i. Fat Percentage ☐☐. ☐☐ %	i. Fat Percentage ☐☐. ☐☐ %
ii. Fat mass - ☐☐. ☐☐ Kg	ii. Fat mass - ☐☐. ☐☐ Kg
iii. Fat free mass ☐☐. ☐☐ Kg	iii. Fat free mass ☐☐. ☐☐ Kg
iv. Pred.muscle mass ☐☐. ☐☐ Kg	iv. Pred.muscle mass ☐☐. ☐☐ Kg
**37.9 Right Arm**	**37.10 Left Arm**
i. Fat Percentage ☐☐. ☐☐ %	i. Fat Percentage ☐☐. ☐☐ %
ii. Fat mass - ☐☐. ☐☐ Kg	ii. Fat mass - ☐☐. ☐☐ Kg
iii. Fat free mass ☐☐. ☐☐ Kg	iii. Fat free mass ☐☐. ☐☐ Kg
iv. Pred.muscle mass ☐☐. ☐☐ Kg	iv. Pred.muscle mass ☐☐. ☐☐ Kg
**37.11 Trunk**	
i. Fat Percentage ☐☐. ☐☐ %	iii. Fat free mass ☐☐. ☐☐ Kg
ii. Fat mass - ☐☐. ☐☐ Kg	iv. Pred.muscle mass ☐☐. ☐☐ Kg

Section H: For Married Children

38.1 How many years have you been married now? ☐☐

38.2 Name of spouse - _____

38.3 Age of spouse - ☐☐

38.4 Current address - _____

_____

38.5 How many children do you have? ☐☐

38.6 Please enter the details of all children in the table below:

(a) Birth order	(b) First name	(c) Date of birth	(d) Gender	(e) Age	(f) Birth weight	(g)Nutrition supplementation recd. Yes=1 and No=2	(h)Age at supplementation.	Status *

*** Status:**

1. Alive and resident in same village
2. Alive and moved to Hyderabad
3. Alive and moved relatively short distance (within 50 kms of Hyderabad, but not to Hyderabad.
4. Alive and moved relatively long distance (i.e. greater than 50 kms from Hyderabad.
5. Died
6. Any other, specify in comments.

Appendix – 5

Andhra Pradesh Children And Parent Study (APCAPS)

REPORT FORM

Date: _ _ / _ _ / _ _ _ _

Subject ID: **Name of the Village:**

Name:

Age: **Sex:**

I. Body Build

Measure	Value	Remarks
**Height (cm)**		
**Weight (kg)**		
**Body Mass Index (BMI)**		**Normal Range: 18-24 kg/m^2**
**Total Body Fat (%)**		**Normal Range:**

II. Physiological Measures

Measure		Value	Remarks
**Blood Pressure (mmHg)**			**Normal: 120/80 mmHg**<br>**High: 140/90 mmHg**
**Lung Function (in litres)**	**FEV1**<br><br>**FEV1/FVC**		**Poor Lung Function:**<br>**FEV1<80%**<br>**FEV1/FVC < 70%**

III. Biochemistry Investigations

Measure	Value	Remarks
**Fasting Glucose (mg/dl)**		**Normal: 70 to 110 mg/dl**<br>**High: >=126 mg/dl**
**Haemoglobin (g/dl)**		**Females: 10-16 g/dl**<br>**Males: 12-18 g/dl**

Note: Kindly verify your readings again with the help of a medical doctor if they are not in the normal range and take his/her advice before starting any treatment

---

Collaborating Centers :South Asia Network for Chronic Disease, PHFI, Delhi; National Institute of Nutrition (NIN), ICMR, Hyderabad; Centre for Chronic Disease Control (CCDC), Delhi; London School of Hygiene and Tropical Medicine (LSHTM), UK; Department of Social Medicine, University of Bristol, UK
Funded by: Wellcome Trust capacity building grant to "South Asia Network for Chronic Disease" (SANCD)

Appendix -6

Body Composition Measurements using TANITA

6.Operating Instructions

GB

Body Composition Analysis

This explains the procedure when the printer function is turned on. Please be aware that the display may be different if the number of printouts is set to [0].

- Do not wipe the equipment with corrosive chemicals (gasoline, cleaner, etc.). Please use a neutral detergent to clean the equipment.
- When the equipment has been transferred to any location where there is a temperature difference of 20°C or more, wait for at least two hours before using it.
- In taking measurements, please keep the person away from the unit, who uses transmitters such as a mobile phone avoid causing margin errors.

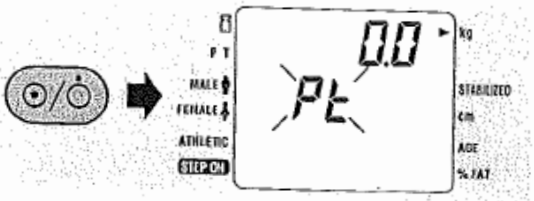

1. Turn on the Power.

Press the [⊙/⏻] key.
"0.0" will appear on the upper portion of the display.

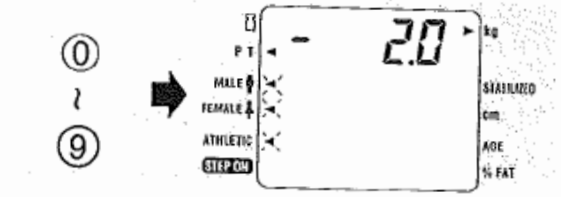

2. Enter Clothes Weight.

Enter clothes weight using the numerical keys.
Example: if the clothes weight is 2.0 kg, press [2], [.] and then [0].
When the data input is completed, the data will be displayed as a minus number.

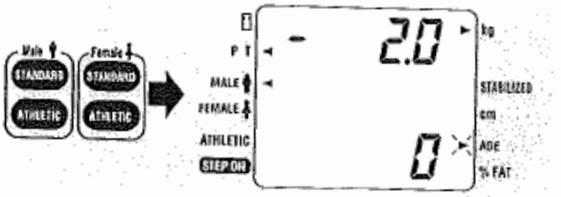

3. Select the Body Type.

Select the Body Type from Standard Male, Standard Female, Athletic Male and Athletic Female. Please use the Athletic key when the user is 17 years or older and also meets the following criteria.
See page 8 for Tanita's definition of "Athlete."

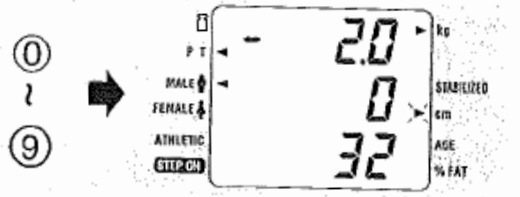

4. Enter Age.

* If the user is 32 years old or younger.
  Example: Press [3] and [2].
* If the user is 9 years old or younger
  Example: press [0] and [9].
* If ages 16 or less are entered, even if Athletic is selected for the Body Type, it will be automatically changed to Standard.

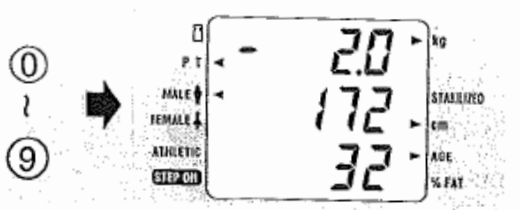

5. Enter Height.

Example: If the user's height is 172 cm, please press [1], [7] and then [2].

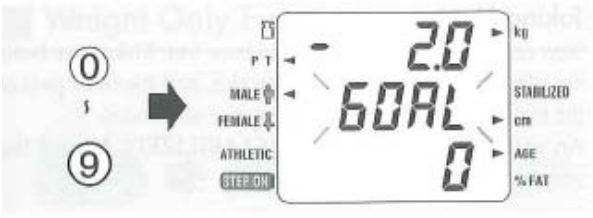

6. Set the Target Body Fat Ratio.

After you input the height, "GOAL" will automatically flash on the display. Input the target body fat ratio desired using the numerical keys.

Example: 16% = Press [1] and [6].
9% = Press [0] and [9].

* If the number of print outs is set to "0", nothing will be displayed.
* If the target body fat ratio is set to OFF, the target body fat ratio will not be printed out.

- **Before you start a body weight management program and set the appropriate personal body fat ratio, please consult your doctor. Tanita is not responsible for setting the appropriate target body fat ratio for specific individuals.**
- **For details of the desirable body fat percentage, please refer to the Technical Notes. Male athletes may wish to select a single digit body fat percentage as their target. However, this is not recommended for Standard Adults, in particular women, who should avoid becoming excessively lean. Always consult a doctor about the target body fat percentage most suitable for your body type.**

- **Do not step on the Weighing Platform until the target body fat ratio setting has been completed because the power may be automatically turned off or the measurement may be inaccurate.**

* If you want to change the settings, please press the [CE] key and the procedure will go back to the former step. Please re-enter the data.

7. After "88888" is displayed on the upper portion of the display, a flashing arrow will appear next to STEP ON .

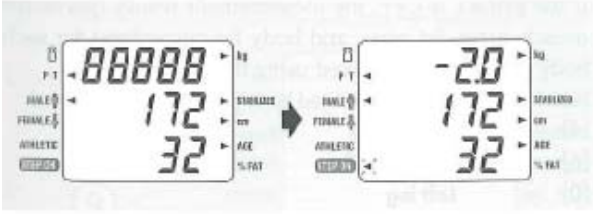

8. Start Measurement.

Step on the Weighing Platform with bare feet so they touch the electrodes. Stand in a stable position without bending your knees.

- **Do not use the hand grips, as you will only be measuring your body weight this time.**

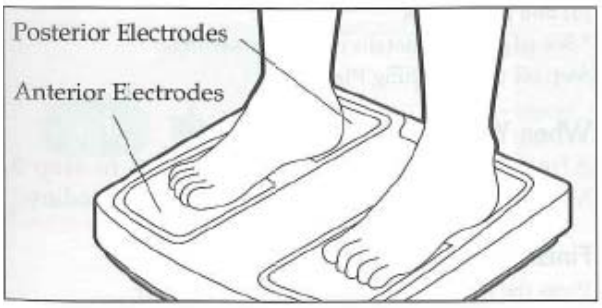

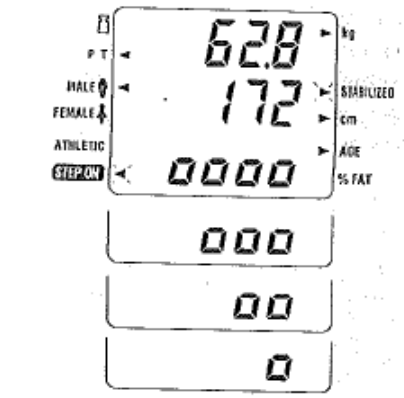

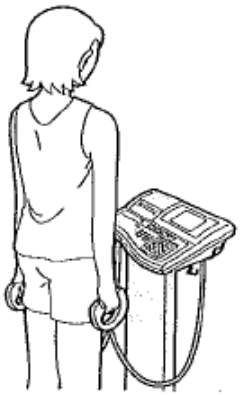

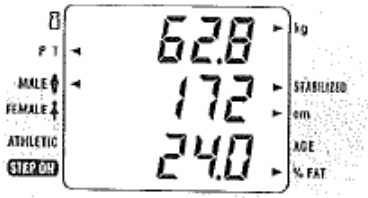

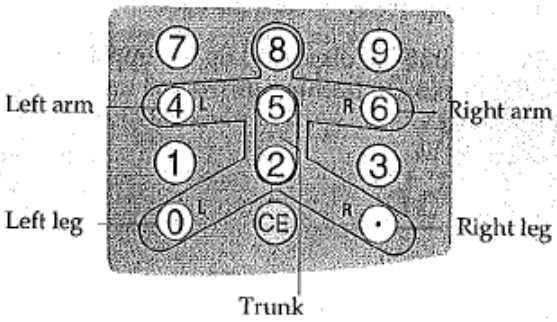

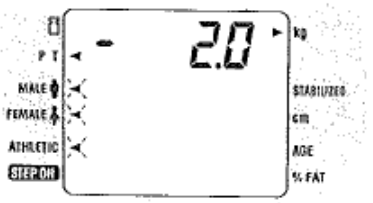

9. Taking Measurement.

Step on the weighing platform in bare feet. Make sure heels are placed on the posterior electrodes, and the front part of the feet are in contact with the anterior electrodes.
An arrow will flash next to [STABILIZED] ▶ and the weight will be displayed on the LCD.

10. Measure the Impedance.

When the grips are grasped with both hands, 0000 will appear at the bottom of the display and the impedance measurement will begin.
The 0000 marks will disappear one by one during the measurement; after five full cycles, the measurement will be complete.

- **Grab the grips (two locations) only after the body weight figure on the display has stabilized.**
- **Do not step off the Weighting Platform until the "0000" symbols disappear completely.**
- **In cases when measurements of the body fat ratio or the quantity of fat are abnormally small or the error message (E01) is shown on the display, the probable reason is that the soles of the feet and the electrodes are not in full contact. Make sure you step on the Weighing Platform so there is contact between the electrodes and the soles of your feet. If the problem is not solved this way, it is possible that the soles of the feet have calluses and the resistance is too great. Therefore, place about 0.5 ml of water on each of the four electrodes where the feet touch before measurement.**

11. Measurement Is Completed.

Once the body weight and impedance measurements have been completed, the overall body fat percentage will be shown at the bottom of the display and a buzzer will sound.
If the printer is ON, the measurement results will be printed out.
* With regard to the measurement result, please refer to P. 22.

If the printer is OFF, the measurement results (predicted muscle mass, fat mass, and body fat percentage) for each body part can be displayed using the ten-key pad.
Select the number of the desired body part on the ten-key pad.

[4]: Left arm
[6]: Right arm
[0]: Left leg
[.]: Right leg
[5] and [2]: Trunk

* See page 13 for details on printer settings.
Step off the Weighing Platform.

12. When You Continue to Measure.

After printing is completed, go back to step 3. Measure by entering the data in the same procedure.

13. Finish Measurement.

Press the [⊙/Ⓞ] key and turn off the power.

Weight Only Function

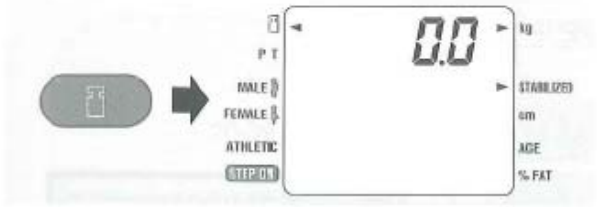

1. After turning on the unit, press the [⏻] key.
After a momentary display check, "0.0" will appear on the LCD. If measuring units need to be changed, do so at this time by pressing the [⏻]key.
An arrow on the LCD will follow the selection of weighing units.

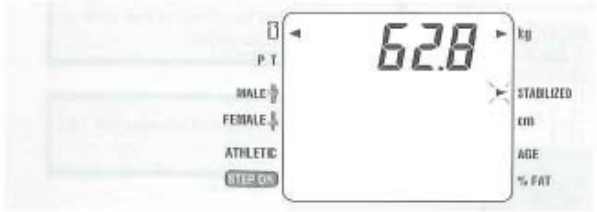

2. Weight Measurement.
Step on the Weighing Platform.An arrow will light next to [STABILIZED] ▶ and the weight measurement will be displayed on the LCD.

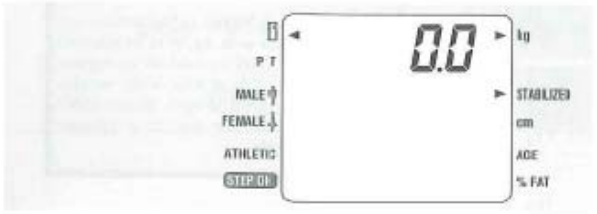

3. When measuring is complete, press the [⊙/⏻] key to turn off the power.

⚠
- **No printer is available when measuring weight only.**
- **If body composition analysis is desired, turn the unit off and then on, using the [⊙/⏻] key.**

Important Note: There is no automatic weight lock function.

Setting of Clothes Weight

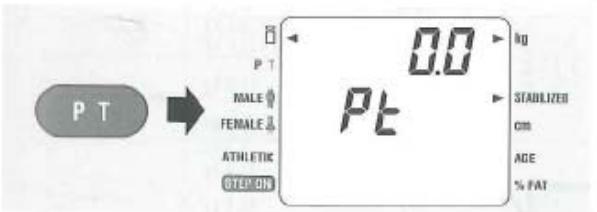

1. Press the [P T] key.

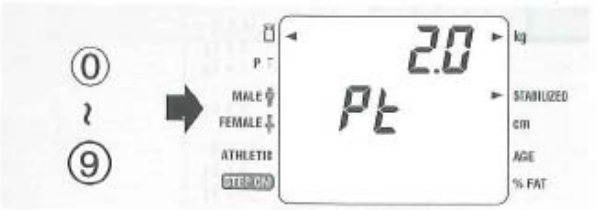

2. Enter the clothes weight.
Example: If the clothes weight is 2.0 kg, press [2], [.] and then [0].
* When you want to correct an entry, press the [CE] key and enter the data again.

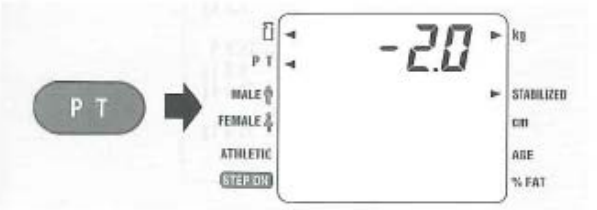

3. Finalizing the setting
Press the [P T] key to finalize the clothes weight.
The finalized clothes weight is displayed with a minus, an arrow next to P T ◀ lights up, and it becomes possible to measure your weight.

Appendix - 7
Fieldworker Booklet
Table of occupational classification in APCAPS

Code number	Name	Activity	Training	Education	Examples
1	At home doing housework	NA	NA	NA	Any member of household
2	Unemployed, not seeking work	NA	NA	NA	Retired, disabled
3	Unemployed, seeking work	NA	NA	NA	Unemployed
4	Student / Training	NA	NA	NA	Student, training,
5	Unskilled manual	Unskilled	None	None	- Landless labourers<br>- unskilled manual labourer,<br>- Servant,<br>- Watchman/chowkidar/gate-keepers<br>- Coolly,<br>- Sweeper/Dhobi(washing and iron)<br>- Hawkers/vendors<br>- Packers, labellors<br>- delivery boys(paper,milk etc)<br>- Garbage collectors<br>- Car cleaner
6	Semi-skilled manual	Semi-skilled, manual	Some training	None	- Marginal landowner<br>- Petty shopkeeper<br>- Peon<br>- Rickshaw driver<br>- Barber<br>- Cobbler<br>- Welder/fitter<br>- Fisherman<br>- Sweet maker(halwai)<br>- Butcher<br>- Farmer/gardener<br>Semi-skilled manual labour

7	Skilled manual	Highly skilled, manual activity	Long training	None or little	- Machine and plant operators<br>- Painters/Plumber<br>- Carpenters/Furnishers<br>- Mason,<br>- Mechanic,<br>- driver, .<br>- Sculptors/potters<br>- Spinners/weavers/carpet makers<br>- Tailors<br>- Blacksmith/goldsmith/engravers<br>- Street artist and performers/circus people<br>- Hunters/trappers<br>- Poultry farmers/animal rearers<br>- Fire-fighters<br>- Army jawan
8	Skilled non-manual	Highly skilled, non-manual activity	Long training	Some education (read, write, arithmetic) will often be required	- Small business owner(<15 employees)<br>- Farm owner/landlord<br>- big Store keeper/ shopkeeper<br>- Clerk/typist/stenographer/librarian<br>- Receptionist(small organization)<br>- Ticket collectors/ sellers & examiners/bus conductors<br>- Playhome teachers<br>- Electrical repair works/ Electrician/watch makers<br>- Telephone/telegraph operators<br>- Post masters/telegraph masters<br>- Station Masters & Station Superintendent<br>- Alternative healers<br>- Musicians/dancers/artists (village level)<br>- Midwives / Health Visitors/field workers/vaccinators<br>- X-ray Technician/Lab technicians/OT assistants.<br>- Postman

9	Semi-professional	Lower grade professional	Lower grade professional training	High school or university education	- Medium business owner (15-49 employees),<br>- Teachers/College lecturers<br>- Personnel managers/Junior Administrators<br>- Inspectors(police,school,insuranceetc.)/Agents(customs,insurances etc.)<br>- Maintenance (in-charge),<br>- Nurses/Pharmacist/Dietician<br>- Accountant<br>- Secretary/ Receptionist(big organisation)<br>- Diploma Engineers<br>- Musicians/dancers/artists (the teachers)
10	Professional	Higher grade professional	Higher grade professional training	Generally but not necessarily university education	- Doctors (Allopathy,Ayurveda,Homeopathy)/Veterinarians<br>- Lawyers/Judges/Magistrate<br>- Engineers/Architects/designers<br>- University lectures/Readers/Professors/Principals<br>- Class I IAS/IFS/IPS officers<br>- Senior administrative officers/Managing Directors<br>- Bank Managers/Auditors<br>- Newspaper Editors<br>- Musicians/dancers/artists (national/international level)<br>- Big business(>50 employees)<br>- Pilots/Navigators

దయచేసి ఈ దిగువనిచ్చిన వాటిలో ప్రతి గ్రూపులో నేటి మీ ఆరోగ్య పరిస్థితిని అత్యుత్తమంగా సూచించే వ్యాఖ్యలకు ఎదురుగా గల గడిలో టిక్ (√) గుర్తు పెట్టండి.

9.1 కదలిక

నడకలో నాకెలాంటి సమస్యలూ లేవు ❑

నడకలో నాక్కొన్ని సమస్యలున్నాయి ❑

నేను మంచానికే పరిమితమై ఉంటాను ❑

9.2 స్వయం -సంరక్షణ

స్వయం- సంరక్షణలో నాకెలాంటి సమస్యలూ లేవు ❑

స్నానం చేయడంలోనూ, లేదా బట్టలు తొడుక్కోవటంలో నాక్కొన్ని సమస్యలున్నాయి ❑

స్నానం చేయడంలోనూ, లేదా బట్టలు తొడుక్కోవటం అస్సలు చెయ్యలేను ❑

9.3 రోజువారీ పనులు (ఉదా: పని చేయడం, చదువు, ఇంటిపని, కుటుంబ సంబంధ పనులు లేదా ఖాళీ సమయంలో చేసే పనులు)

రోజువారీ పనులు చేసే విషయంలో నాకెలాంటి సమస్యలూ లేవు ❑

రోజువారీ పనులు చేసే విషయంలో నాక్కొన్ని సమస్యలున్నాయి ❑

నేను రోజువారీ పనులు అస్సలు చెయ్యలేను ❑

9.4 నొప్పి / అసౌకర్యం

నాకు ఎలాంటి నొప్పి లేదా అసౌకర్యం లేవు ❑

నాకు ఒక మాదిరి నొప్పి లేదా అసౌకర్యం ఉన్నాయి ❑

నాకు తీవ్రమైన నొప్పి లేదా అసౌకర్యం ఉన్నాయి ❑

9.5 ఆత్రుత/ నిరుత్సాహం

నేను ఆత్రుత లేదా నిరుత్సాహంతో లేను ❑

నేను ఒక మాదిరి ఆత్రుత లేదా నిరుత్సాహంతో ఉన్నాను ❑

నేను తీవ్రమైన ఆత్రుత లేదా నిరుత్సాహంతో ఉన్నాను ❑

9.6 ఆరోగ్యపరిస్థితి ఎంత చక్కగా లేదా ఎంత అధ్వాన్నంగా ఉందో చెప్పేందుకు ప్రజలకు సహాయపడేందుకు మేము గీసిన కొలబద్ద (ధర్మోమీటర్ మాదిరిగా)పై, అత్యంత మెరుగైన ఆరోగ్య పరిస్థితిగా మీకు అనిపించేది 100 గాను మరియు అత్యంత క్షీణస్థితిగా మీకు అనిపించేది ం గాను గుర్తింబడ్డాయి.

మీ అభిప్రాయం ప్రకారం ఈ రోజున మీ ఆరోగ్య పరిస్థితి ఎంత చక్కగా లేదా ఎంత ఆధ్వాన్నంగా ఉందో మీరు ఈ కొలమానంపై గుర్తించాలని మేము కోరుతున్నాం. దయచేసి దిగువ ఇవ్వబడిన గడి నుండి, కొలమానంపై ఉన్న బిందువు వరకూ మీ ఆరోగ్యం ఎంత మెరుగ్గా లేదా క్షీణంగా ఉందో, అంతవరకు గీత గీస్తూ దాన్ని చెయ్యండి.

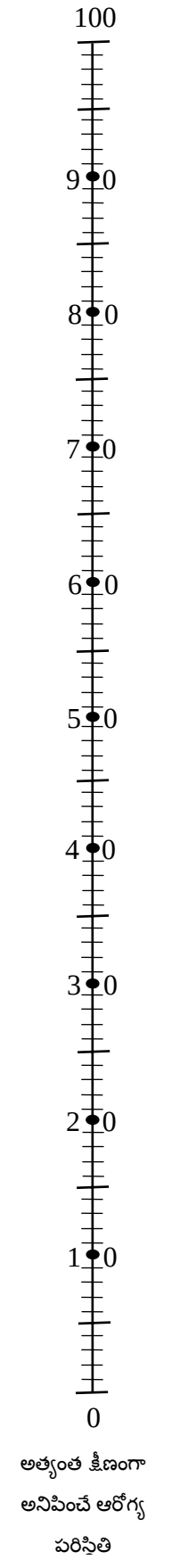

రోగి ఆరోగ్య సంక్షిప్త ప్రశ్నావళి

గత రెండు వారాలలో ఎంత తరచుగా ఈ క్రింది సమస్యల వలన మీరు భాధపడ్డారు?

1 = ఏమాత్రం లేదు 2 = అనేక రోజులు 3 = సగం కంటే ఎక్కువ రోజులు 4 = దాదాపు ప్రతిరోజు

9.7 పని చేయడంలో తక్కువ ఆసక్తి లేదా ఆనందం కలిగింది.	☐
9.8 నిస్పృహ, నిరాశ లేదా దు:ఖ భావన కలిగింది.	☐
9.9 నిద్ర రావడం/నిద్ర పోయి ఉండడంలో కష్టం లేదా నిద్ర ఎక్కువవడం	☐
9.10 తొందరగా అలసిపోవడం లేదా నీరసపడడం	☐
9.11 తక్కువ ఆకలి వేయడం లేదా ఎక్కువ తినడం	☐
9.12 మీరు మీ స్వంతానికి గాని, కుటుంబానికి గాని ఏమి చేయలేదని, మీరొక ఓడిపోయిన వ్యక్తనే చెడు భావనలు కలగడం.	☐
9.13 పేపరు చదవడం లేదా టెలివిజన్ చూడడం వంటి పనులలో ఎందులోను ఏకాగ్రత కలగకపోవడం	☐
9.14 ఇతరులు గమనించేటంతగా మెల్లగా నడవడం, మాట్లాడడం లేదా చికాగ్గా, అసహనంగా, అస్థిరంగా తిరగడం.	☐
9.15 మీ మనసులో చచ్చిపోవాలనే లేదా స్వయంగా గాయపరచుకోవాలనే ఆలోచన వచ్చింది.	☐

అతృత గురించిన ప్రశ్నలు

1 = అవును 2 = లేదు.

9.16 **గత 4 వారాలలో** హఠాత్తుగా భయపడ్డం లేదా ఆందోళన పడడం వంటి ఎటాక్ వచ్చిందా? **మీరు "లేదు" అని సమాధానం ఇస్తే 9.21 ప్రశ్నకు వెళ్ళండి.**	☐
9.17 ఇంతకు ముందు ఎప్పుడైనా ఇలా జరిగిందా ?	☐
9.18 వీటిలో కొన్ని సంఘటనలు **హఠాత్తుగా ఆందోళన** లేదా చికాకు ఊహించని సందర్భాలలో కలుగుతాయా ?	☐
9.19 ఎటాక్ వలన మీకు చికాకు కలుగుతుందా ? మరోసారి ఇలా జరుగుతుందని భయపడ్తున్నారా ?	☐
9.20 మీ ఆఖరి ఆందోళన సంఘటన కలిగినప్పుడు ఊపిరి ఆడకపోవడం, చెమటులు పట్టడం, గుండె గబా గబా కొట్టుకోవడం, తల తిరగడం లేదా స్పృహ లేకపోవడం, తిమ్మిరులు కలగడం, వాంతులు లేదా వికారం కలగడం వంటి లక్షణాలు కనిపించాయా ?	☐

9.21 ఒక వేళ ఈ ప్రశ్నావళిలో ఇచ్చిన ఏమైనాసమస్యలు ఇప్పటివరకు మీకు చికాకు కలిగిస్తే, ఈ సమస్యల కారణంగా మీకు మీ పనిని చేయడం, ఇంటి విషయాలను పట్టించుకోవడం లేదా ఇతరులతో కలసిమెలసి ఉండటంలో ఎంత కష్టం కలిగిందో చెప్పండి. ☐

1 = ఏమాత్రం కష్టం లేదు. 2 = కొంత వరకు కష్టం అయింది 3 = చాలా కష్టం అయింది.
4 = చాలా ఎక్కువ కష్టం అయింది.

గత నాలుగు వారాలలో ఈ క్రింది సమస్యల వలన మీరు ఎంత చికాకు పడ్డారు ?

1 = చికాకు లేదు. 2 = కొంచెం చికాకు 3 = చాలా చికాకు

9.22 మీ ఆరోగ్యం గురించి చికాకు పడడం	☐
9.23 మీ రూపం/బరువు గురించి కలత.	☐
9.24 భర్త / భార్య / తల్లిదండ్రులు / ఇతర కుటుంబ సభ్యులతో కష్టాలు	☐
9.25. పిల్లలు, తల్లిదండ్రులు, కుటుంబ సభ్యుల బాగోగులు చూడడంలో వత్తిడి.	☐
9.26 స్కూలు / ఇంటి బయట వత్తిడి.	☐
9.27 ఆర్థిక ఇబ్బందులు	☐
9.28 కష్టంలో పాలు పంచుకునే ఆప్తులు లేకపోవడం	☐
9.29 ఈ మధ్య కాలంలో జరిగిన చెడు గురించి పట్టించుకుంటారా ?	☐
9.30 **గతంలో** జరిగిన విపరీత సంఘటన (ఉదా : ఇల్లు ధ్వంసమయిపోవడం, ఘోరప్రమాదం, ఎవరి చేతనో కొట్టబడడం / దాడి చేయడబడడం లేదా బలాత్కరించబడడం) గురించి ఆలోచించడం, ఆ సంఘటనలు కలలోనికి రావడం.	☐

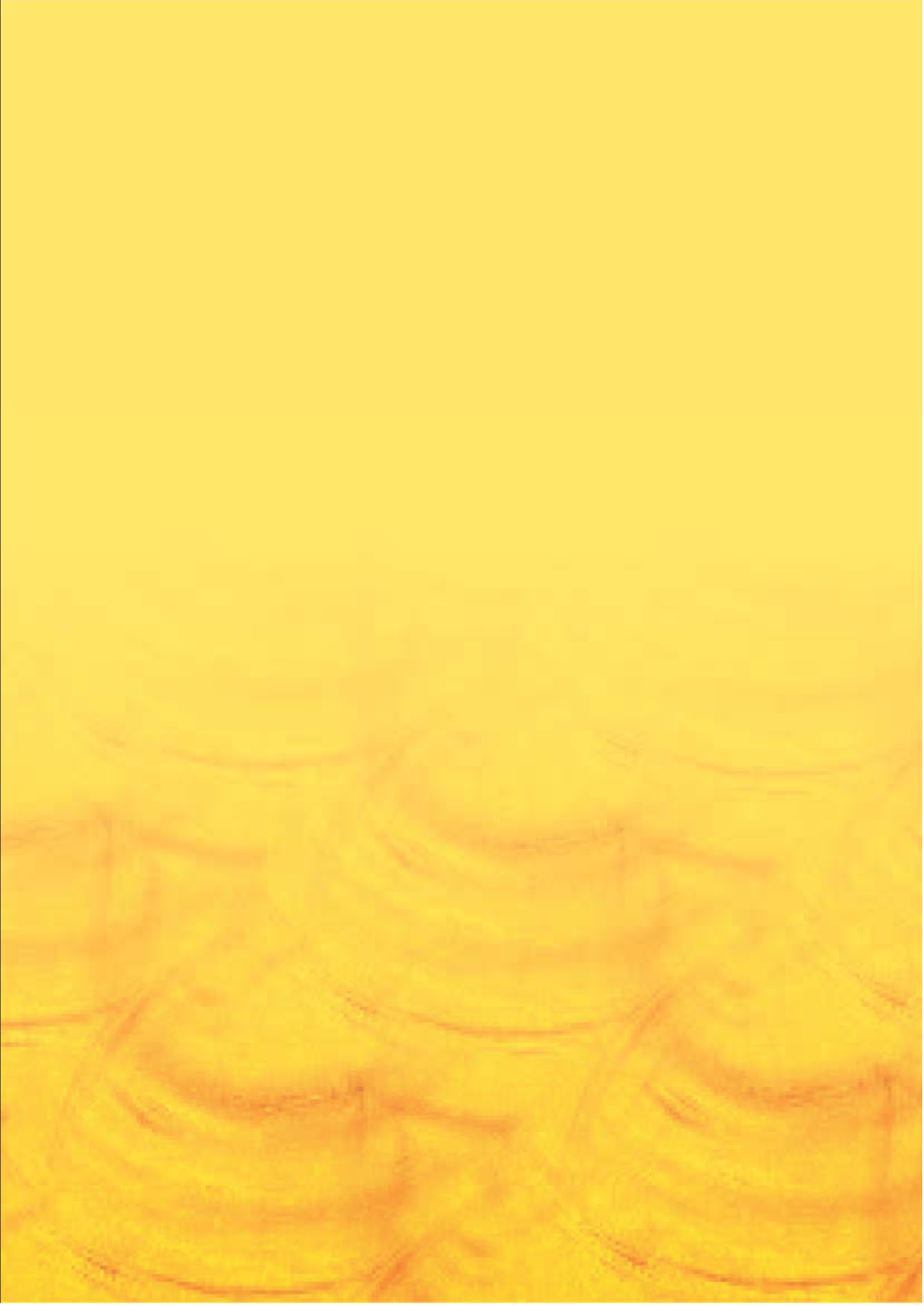

Supplement: S1 Text — APCAPs, Andhra Pradesh Children and Parents study. (PDF) [file pmed.1003183.s002.pdf]
