# Supplementary material for: Effect of supplemental nutrition in pregnancy on offspring’s risk of cardiovascular disease in young adulthood: Long-term follow-up of a cluster trial from India
Source: PLoS Med. 2020 Jul 21;17(7):e1003183. doi: 10.1371/journal.pmed.1003183 (PMC7373266; doi:10.1371/journal.pmed.1003183)
Supplement: S1 Table — (DOCX) [file pmed.1003183.s003.docx]

| **Cardiovascular risk factor** | **N** | **Estimated effect (beta-coefficient) of supplementation (95% confidence interval)** | | **p-value interaction** |  |
| --- | --- | --- | --- | --- | --- |
|  |  | **Age<22 year** | **Aged>=22 year** |  |  |
| Height (mm) | 1783 | -1.62 (-9.82 to 6.57) | 1.32 (-8.60 to 11.23) | 0.63 |  |
| BMI (kg/m^2^) | 1783 | -0.11 (-0.64 to 0.43) | -0.29 (-0.90 to 0.31) | 0.57 |  |
| Waist circumference (mm) | 1779 | -2.01 (-14.57 to 10.56) | -9.04 (-23.44 to 5.36) | 0.39 |  |
| Systolic BP (mmHg) | 1782 | 0.96 (-0.48 to 2.40) | 0.43 (-1.23 to 2.08) | 0.58 |  |
| Diastolic BP (mmHg) | 1782 | 1.20 (-0.34 to 2.74) | 1.18 (-0.56 to 2.92) | 0.98 |  |
| Central SBP (mmHg) | 1395 | -0.36 (-1.66 to 0.95) | 0.63 (-1.58 to 2.85) | 0.42 |  |
| Pulse wave velocity | 1542 | 0.05 (-0.06 to 0.15) | 0.04 (-0.13 to 0.20) | 0.92 |  |
| Pulse pressure (mmHg) | 1782 | -0.35 (-1.10 to 0.40) | -0.78 (-1.70 to 0.14) | 0.47 |  |
| Augmentation index (%) | 1322 | -1.57 (-3.24 to 0.10) | 1.16 (-1.04 to 3.35) | 0.008 |  |
| Carotid IMT (mm) | 1194 | -0.01 (-0.04 to 0.01) | 0.03 (-0.01 to 0.06) | 0.016 |  |
| Total cholesterol (mmol/l) | 1764 | 0.09 (-0.12 to 0.30) | 0.11 (-0.12 to 0.34) | 0.81 |  |
| LDL cholesterol (mmol/l) | 1756 | 0.10 (-0.06 to 0.26) | 0.09 (-0.08 to 0.27) | 0.93 |  |
| HDL cholesterol (mmol/l) | 1764 | -0.02 (-0.10 to 0.05) | 0.00 (-0.08 to 0.08) | 0.40 |  |
| Log triglycerides (mmol/l) | 1763 | 0.02 (-0.05 to 0.09) | 0.04 (-0.04 to 0.12) | 0.65 |  |
| Fasting glucose (mmol/l) | 1763 | 0.12 (-0.02 to 0.26) | -0.20 (-0.36 to -0.03) | 0.001 |  |
| Log insulin (mU/l) | 1756 | 0.17 (-0.00 to 0.34) | -0.04 (-0.23 to 0.15) | 0.016 |  |
| Log HOMA-IR | 1756 | 0.15 (-0.02 to 0.32) | -0.06 (-0.25 to 0.12) | 0.010 |  |

**S1 Table.** Age-and sex adjusted effect of supplemental nutrition by age at outcome measurement

BMI is Body Mass Index; BP is Blood Pressure, IMT is Intima-Media Thickness; LDL is Low-density lipoprotein, HDL is High-density lipoprotein, HOMA-IR is Homeostatic Model Assessment-Insulin Resistance
