# Supplementary material for: Effect of supplemental nutrition in pregnancy on offspring’s risk of cardiovascular disease in young adulthood: Long-term follow-up of a cluster trial from India
Source: PLoS Med. 2020 Jul 21;17(7):e1003183. doi: 10.1371/journal.pmed.1003183 (PMC7373266; doi:10.1371/journal.pmed.1003183)
Supplement: S2 Table — (DOCX) [file pmed.1003183.s004.docx]

**S2 Table.** Age-adjusted effect of supplemental nutrition on outcome by sex of the participant

| **Cardiovascular risk factor** | **N** | **Estimated effect (beta coefficient) of supplementation (95% confidence interval)** | | **p-value interaction** |
| --- | --- | --- | --- | --- |
|  |  | **Male** | **Female** |  |
| Height (mm) | 1783 | -2.49 (-10.63 to 5.66) | 3.12 (-7.18 to 13.42) | 0.37 |
| BMI (kg/m^2^) | 1783 | 0.07 (-0.45 to 0.60) | -0.60 (-1.22 to 0.01) | 0.04 |
| Waist circumference (mm) | 1779 | -1.41 (-13.67 to 10.84) | -10.52 (-25.22 to 4.18) | 0.27 |
| Systolic BP (mmHg) | 1782 | 0.98 (-0.43 to 2.39) | 0.36 (-1.34 to 2.06) | 0.53 |
| Diastolic BP (mmHg) | 1782 | 1.27 (-0.25 to 2.79) | 1.06 (-0.72 to 2.85) | 0.83 |
| Central SBP (mmHg) | 1395 | 0.00 (-1.41 to 1.42) | -0.38 (-2.14 to 1.38) | 0.72 |
| Pulse wave velocity | 1542 | 0.07 (-0.04 to 0.18) | -0.01 (-0.14 to 0.13) | 0.28 |
| Pulse pressure (mmHg) | 1782 | -0.38 (-1.12 to 0.36) | -0.75 (-1.69 to 0.20) | 0.54 |
| Augmentation index (%) | 1322 | -1.45 (-3.20 to 0.30) | 0.07 (-1.89 to 2.04) | 0.09 |
| Carotid IMT (mm) | 1194 | 0.01 (-0.02 to 0.03) | -0.02 (-0.05 to 0.02) | 0.17 |
| Total cholesterol (mmol/l) | 1764 | 0.11 (-0.11 to 0.32) | 0.09 (-0.14 to 0.32) | 0.86 |
| LDL cholesterol (mmol/l) | 1756 | 0.09 (-0.06 to 0.25) | 0.11 (-0.06 to 0.28) | 0.83 |
| HDL cholesterol (mmol/l) | 1764 | -0.01 (-0.09 to 0.06) | -0.01 (-0.09 to 0.07) | 0.97 |
| Log triglycerides (mmol/l) | 1763 | 0.04 (-0.03 to 0.11) | 0.01 (-0.07 to 0.10) | 0.57 |
| Fasting glucose (mmol/l) | 1763 | 0.00 (-0.14 to 0.14) | -0.02 (-0.19 to 0.15) | 0.83 |
| Log insulin (mU/l) | 1756 | 0.09 (-0.08 to 0.26) | 0.07 (-0.11 to 0.26) | 0.85 |
| Log HOMA-IR | 1756 | 0.09 (-0.08 to 0.25) | 0.03 (-0.15 to 0.22) | 0.53 |

BMI is Body Mass Index; BP is Blood Pressure, IMT is Intima-Media Thickness; LDL is Low-density lipoprotein, HDL is High-density lipoprotein, HOMA-IR is Homeostatic Model Assessment-Insulin Resistance
