# Supplementary material for: Effect of supplemental nutrition in pregnancy on offspring’s risk of cardiovascular disease in young adulthood: Long-term follow-up of a cluster trial from India
Source: PLoS Med. 2020 Jul 21;17(7):e1003183. doi: 10.1371/journal.pmed.1003183 (PMC7373266; doi:10.1371/journal.pmed.1003183)
Supplement: S3 Table — (DOCX) [file pmed.1003183.s005.docx]

**S3 Table.** Effect of supplemental nutrition by village urbanisation score

| **Cardiovascular risk factor** | **N** | **Estimated effect (beta coefficient) of supplementation (95% confidence interval)** | | | **p-value interaction** |
| --- | --- | --- | --- | --- | --- |
|  |  | **Least urbanized third** | **Middle third of urbanisation** | **Most urbanized third** |  |
| Height (mm) | 1783 | 0.22 (-12.53 to 12.97) | 1.94 (-10.09 to 13.97) | -2.86 (-14.20 to 8.49) | 0.85 |
| BMI (kg/m^2^) | 1783 | -0.49 (-1.27 to 0.29) | -0.46 (-1.20 to 0.28) | 0.02 (-0.74 to 0.78) | 0.52 |
| Waist circumference (mm) | 1779 | -12.09 (-30.15 to 5.97) | -10.29 (-27.51 to 6.93) | -1.04 (-18.44 to 16.37) | 0.56 |
| Systolic BP (mmHg) | 1782 | -0.58 (-2.47 to 1.31) | 0.65 (-1.14 to 2.43) | 1.13 (-0.55 to 2.81) | 0.60 |
| Diastolic BP (mmHg) | 1782 | 0.17 (-1.72 to 2.07) | 1.03 (-0.76 to 2.82) | 1.12 (-0.59 to 2.83) | 0.76 |
| Central SBP (mmHg) | 1395 | 0.16 (-1.87 to 2.18) | -0.03 (-1.99 to 1.93) | -0.48 (-2.31 to 1.34) | 0.71 |
| Pulse wave velocity | 1542 | -0.00 (-0.14 to 0.13) | 0.01 (-0.12 to 0.14) | 0.07 (-0.05 to 0.18) | 0.48 |
| Pulse pressure (mmHg) | 1782 | -0.74 (-1.89 to 0.40) | -0.35 (-1.43 to 0.72) | -0.05 (-1.06 to 0.97) | 0.26 |
| Augmentation index (%) | 1322 | -2.96 (-5.46 to -0.47) | 2.11 (-0.33 to 4.55) | -1.59 (-4.15 to 0.96) | 0.28 |
| Carotid IMT (mm) | 1194 | 0.04 (0.01 to 0.07) | -0.00 (-0.03 to 0.03) | -0.03 (-0.06 to 0.00) | 0.006 |
| Total cholesterol (mmol/l) | 1764 | -0.07 (-0.42 to 0.27) | 0.09 (-0.24 to 0.42) | 0.19 (-0.16 to 0.54) | 0.39 |
| LDL cholesterol (mmol/l) | 1756 | -0.02 (-0.24 to 0.21) | 0.08 (-0.14 to 0.29) | 0.07 (-0.15 to 0.30) | 0.697 |
| HDL cholesterol (mmol/l) | 1764 | -0.03 (-0.14 to 0.09) | -0.01 (-0.12 to 0.10) | 0.09 (-0.02 to 0.21) | 0.14 |
| Fasting glucose (mmol/l) | 1763 | -0.01 (-0.21 to 0.19) | 0.01 (-0.18 to 0.20) | -0.08 (-0.27 to 0.11) | 0.75 |
| Log triglycerides (mmol/l) | 1763 | -0.03 (-0.14 to 0.07) | 0.02 (-0.07 to 0.12) | 0.07 (-0.03 to 0.17) | 0.32 |
| Log insulin (mU/l) | 1756 | -0.09 (-0.30 to 0.11) | -0.02 (-0.22 to 0.18) | 0.07 (-0.14 to 0.27) | 0.50 |
| Log HOMA-IR | 1756 | -0.10 (-0.31 to 0.11) | -0.02 (-0.22 to 0.18) | 0.02 (-0.18 to 0.23) | 0.65 |

BMI is Body Mass Index; BP is Blood Pressure, IMT is Intima-Media Thickness; LDL is Low-density lipoprotein, HDL is High-density lipoprotein, HOMA-IR is Homeostatic Model Assessment-Insulin Resistance
