# Supplementary material for: Effect of supplemental nutrition in pregnancy on offspring’s risk of cardiovascular disease in young adulthood: Long-term follow-up of a cluster trial from India
Source: PLoS Med. 2020 Jul 21;17(7):e1003183. doi: 10.1371/journal.pmed.1003183 (PMC7373266; doi:10.1371/journal.pmed.1003183)
Supplement: S4 Table — BMI, body mass index. (DOCX) [file pmed.1003183.s006.docx]

**S4 Table.** Effect of supplemental nutrition by body mass index of participant

| **Cardiovascular risk factor** | **N** | **Estimated effect (beta coefficient) of supplementation (95% confidence interval)** | | **p-value interaction** |
| --- | --- | --- | --- | --- |
|  |  | **BMI≤20kg/m^2^** | **BMI>20kg/m^2^** |  |
| Height (mm) | 1783 | 1.42 (-6.79 to 9.63) | 1.26 (-7.76 to 10.29) | 0.98 |
| Waist circumference (mm) | 1779 | -6.74 (-14.4 to 0.94) | -1.56 (-9.98 to 6.86) | 0.31 |
| Systolic BP (mmHg) | 1782 | 0.64 (-0.80 to 2.08) | 1.22 (-0.32 to 2.78) | 0.48 |
| Diastolic BP (mmHg) | 1782 | 0.99 (-0.40 to 2.38) | 1.40 (-0.10 to 2.90) | 0.62 |
| Central SBP (mmHg) | 1395 | 0.08 (-1.37 to 1.53) | 0.08 (-1.52 to 1.68) | 1.00 |
| Pulse wave velocity | 1542 | 0.04 (-0.05 to 0.14) | 0.06 (-0.05 to 0.16) | 0.81 |
| Pulse pressure (mmHg) | 1782 | -0.38 (-1.23 to 0.47) | -0.12 (-1.05 to 0.80) | 0.64 |
| Augmentation index (%) | 1322 | -0.35 (-1.99 to 1.29) | -2.00 (-3.72 to -0.28) | 0.035 |
| Carotid IMT (mm) | 1194 | 0.01 (-0.02 to 0.03) | 0.01 (-0.02 to 0.04) | 0.49 |
| Total cholesterol (mmol/l) | 1764 | 0.08 (-0.12 to 0.28) | 0.18 (-0.03 to 0.39) | 0.24 |
| LDL cholesterol (mmol/l) | 1756 | 0.09 (-0.07 to 0.25) | 0.19 (0.02 to 0.35) | 0.16 |
| HDL cholesterol (mmol/l) | 1764 | -0.01 (-0.09 to 0.06) | -0.02 (-0.10 to 0.05) | 0.65 |
| Log triglycerides (mmol/l) | 1763 | 0.01 (-0.05 to 0.07) | 0.05 (-0.01 to 0.12) | 0.27 |
| Fasting glucose (mmol/l) | 1763 | -0.04 (-0.16 to 0.07) | 0.01 (-0.11 to 0.14) | 0.49 |
| Log insulin (mU/l) | 1756 | 0.01 (-0.15 to 0.17) | 0.08 (-0.08 to 0.25) | 0.28 |
| Log HOMA-IR | 1756 | -0.02 (-0.18 to 0.14) | 0.07 (-0.09 to 0.24) | 0.19 |

BMI is Body Mass Index; BP is Blood Pressure, IMT is Intima-Media Thickness; LDL is Low-density lipoprotein, HDL is High-density lipoprotein, HOMA-IR is Homeostatic Model Assessment-Insulin Resistance
