# Supplementary material for: Effect of supplemental nutrition in pregnancy on offspring’s risk of cardiovascular disease in young adulthood: Long-term follow-up of a cluster trial from India
Source: PLoS Med. 2020 Jul 21;17(7):e1003183. doi: 10.1371/journal.pmed.1003183 (PMC7373266; doi:10.1371/journal.pmed.1003183)
Supplement: Attachment — Submitted filename: Response to reviewers_Kinra et al.docx [file pmed.1003183.s007.docx]

**The Editor**

**PLOS Medicine**

Dear Editor,

We thank the editors and reviewers for their thorough and insightful comments on our manuscript. We found these comments extremely helpful in improving our manuscript. Please see our point by point response to these comments in bold letters below with line numbers referring to revised manuscript submitted alongside.

Please do not hesitate if you have any follow-on questions or suggestions.

Best wishes,

Sanjay Kinra, on behalf of the co-authors

**Comments from the Academic Editor:**
The authors examined the intervention effects of food supplements in a unique trial conducted in three decades ago on profiles of cardiovascular risk factors.

Main comments:
1. The reviewer initially somehow read as if this study were a community-based cluster randomized controlled trial. It appeared not, where the program did not randomly determine intervention villages and control villages (confirmed in Ref #12, BMJ article). The non-randomized design should be apparent in Abstract at least. Also, the authors should state the selection criteria for the intervention and no intervention explicitly in the first paragraph of Methods. The authors are encouraged to state clearly, in the abstract and the first paragraph of the Methods, that this study was a community-based non-randomized controlled intervention trial.

**We agree that the villages were not formally randomised, although they were selected using an objective selection process to minimise confounding (i.e. contiguous villages surrounding the geographic centre of two adjacent administrative areas). We have now used the suggested terminology ‘community-based non-randomised controlled intervention trial’ throughout (including abstract and first paragraph of methods), explained the village selection criteria in greater detail in the methods, and also included a map as suggested later by the editors (lines 117-123 and Fig 1).**

2. The authors found null associations in the primary analysis and described why in Discussion. The authors described the reason that the null finding might be due to the confounding effects of puberty or related factors. This explanation does not make sense. The confounding factor must have occurred before the exposure status was determined. The primary exposure was the intervention to mothers of the participants and to the participants themselves during their childhoods. Puberty factors could not be confounders.

**The relevant statement (‘differences in cardiovascular risk factors in adolescence may have been due to chance or residual confounding from differences in pubertal status, which may not have been fully accounted for by our pubertal assessment tool; both under- and over-nutrition have been associated with changes in timing and progression through puberty, which can influence the levels of cardio-metabolic risk factors in a variable fashion.’) meant to suggest puberty as a random factor or a mediator of positive findings in the 1^st^ follow-up. The term residual confounding was therefore misleading but was intended to refer to inadequate adjustment by the puberty assessment tool. This hypothesis is supported by the earlier growth spurt in the intervention arm (participants were taller at age 16 years, but not at age 22 years), which is widely regarded a strong marker of puberty timing. Nevertheless, since this explanation refers to findings from 1^st^ follow-up (and already discussed in the relevant publication). We are happy to remove this explanation.**

The possible explanations should include the true null, flagging up the possibility of by-chance findings in the previous analysis (BMJ). This current study, showing Table 3 and 4, indicated the selection bias in the previous analysis. The participants selectively showed up the first follow-up and presented favourable outcomes, thereby leading to potentially false-positive results. Also, the previous analysis in BMJ demonstrated a lot of statistical tests and may have substantially raised the likelihood of false-positive findings: the authors should take a fresh look at the results with the null. The authors should suspect such possibilities.

Then, the authors may want to highlight the overall cohort design, given the non-random nature of the exposure and also non-random nature of the responses to the follow-up assessments. It would be difficult to solve, but that should be the point to highlight.

**We have taken these suggestions on board (thank you for these thoughtful suggestions) and revised the relevant paragraphs of the discussion section accordingly. Please see lines 337-371 (limitations of the study) and 391-414 (comparison of findings from 1^st^ and 2^nd^ or 3^rd^ follow-ups).**

3. The other possible explanation of the findings was that any positive or adverse effect of the intervention might get diluted over the long-term follow-up given the nutrition or economic transition over time. The authors are encouraged to extract such information from regional data.

**These data are part of two independent publications (one published and other under late stages of peer review with a journal). We have provided some key text (lines 398-405) and citation of the published paper (and hope to provide the second citation as soon as the acceptance is confirmed).**

4. The authors should explore the literature and discuss whether the dose levels were sufficient to test the hypothesis. 2.5 MJ of energy intake and 20 g of protein on a single day or over a few months (frequency and duration were not available in this paper actually) may not have been sufficient. The authors should look at the requirement of energy intake and protein intake for pregnant women and the toddlers and compare between those required levels and the provided levels via food supplements. It would be acceptable that the authors discuss their speculation. However, before the speculation (e.g. puberty effect) without physiologic explanation, the authors seem to have a lot to discuss.

**We have now discussed this issue in lines 351-358. Supplement is/was given daily (we apologise for inadvertently missing this information) and provides ~25% of daily energy and protein requirement for the women, and ~25% of energy and ~50% of protein requirement for the children.**

5. The authors should confirm or disconfirm that the intervention villages were selected because of their socially deprived condition. If so, the authors were testing whether the food supplements let people in deprived villages catch up with those in more affluent villages. This interpretation would be optimistic but fair. The authors may want to touch on this while no randomization was certainly the limitation and to be on emphasis much more. At the same time, the authors describe the difference between pregnant women in intervention villages and control villages.

**The intervention villages were not selected due to their socially deprived conditions. The national program (i.e. intervention) was gradually rolled out across the 1 million villages of India over a decade due to logistical constraints. However, we have now acknowledged the possibility of differences between areas arising because of non-randomisation by adding the following statement to the discussion, ‘Although the control district was awaiting implementation of the intervention at the time of the trial (and did receive it a few years later in 1992-1993), the intervention district may have may have received the intervention earlier because of some other reason (e.g. political), which could be associated with other differences between the trial areas that could influence health outcomes.’ (see lines 339-343).**

Minor comments:
Abstract:
"cluster trial" should be "cluster non-randomized trial".

**Done (line 32).**

Introduction:
"maybe" should be "may be" in the end of the first paragraph.

**Done (line 98).**

The sentence "We previously reported..." should be taken out.

**Done. We were not entirely certain, but we have interpreted this comment to mean that the editor would like us to remove reference to the previous findings from the same cohort from introduction. We have now done that (and also removed reference to previous finding from the abstract for consistency) and only refer to these findings after describing the trial (i.e. in methods and discussion). We agree that it will be less confusing for the reader in this way.**

The abstract should include limitations of no randomization or residual confounding and no assessment of compliance with the intervention at least.

**Done (lines 44-46).**

Levels of dose, frequency, and duration of the intervention should be available in Abstract.

**Done (lines 33-35).**

The conclusion requires revision. The authors should not give a conclusion about what this cohort will have to do: readers may support the promise of this study, but that cannot be the conclusion of this study. The last sentence should be taken out.

**Done.**

Methods:
"South India" sounds like a country. "India" seems fine.

**Done (line 110).**

On the third line of the first paragraph, "aims" may sound too odd because the trial started long ago and "ICDS" is not clear for readers (and the reviewer) whether it has been lasting for a long time. The authors may want to supplement the information that ICDS is a long-term scheme lasting over decades in India to let the tense make sense.

**Done (line 110-114).**

The authors should clarify the dose frequency and duration of the food supplement, not only the dose levels. Readers could interpret that the investigators had provided just a single package of food.

**The supplement was/is offered daily to pregnant and lactating women and children below the age of 6 years (apologies as this was inadvertently left out) (see lines 123-126). It is now clarified throughout.**

In the second paragraph of Methods, "Where individual participant data was" should be "Where individual participant data were".

**Done (line 154).**

The authors stated assay quality, but IMT and some of the other measures are not assays. The authors need to rephrase or add sufficient information on a measurement quality for different physiologic measures.

**We have rephrased and added data on measurement quality for physiological measures (lines 211-215).**

Here are some comments about statistical analyses.
"We fitted a random-effect at both village and household levels" is not clear. The authors could assign a random-effect to different parameters in a regression model. Those could be an intercept, a beta coefficient for the binary intervention variable, or both. The current description is not clear. It is highly important to test or describe the variation of effects across villages.

**We used a model with a random intercept at the village and household level. We were mainly concerned that outcomes may be clustered rather than the effect of the intervention varying between villages, hence the inclusion of a random intercept rather than a random slope. To clarify this, we have changed the relevant sentence to say “random-intercept” model (line 226).**

In the statistical section, the authors should explicitly state the need for the statistical adjustment because the trial was susceptible to the bias without randomization.

**Done (lines 234-236).**

It would be meaningful to adjust for mediating or intermediary factors. However, the factors the authors describe just after the thought should not be age and sex. They cannot be mediating factors. The authors may want to revise the order of the description would allow an odd interpretation. Alternatively, the authors should state what they think are mediating factors.

**By intermediary factors, we were referring to some lifestyle factors included in model 3 (e.g. dietary factors such as preference for fatty foods or sedentariness), which could potentially be determined by early life nutritional status. However, since the evidence for this is weak, we have now deleted the reference to intermediary factors. The revised statement now reads,** **‘In view of the non-randomised trial design and varying urbanisation of the study villages over the long-term follow-up, we fitted three predefined models to incrementally adjust for potential differences in socio-demographic and lifestyle factors that could influence the levels of cardiovascular risk factors: model 1 was adjusted for age and sex; model 2 was additionally adjusted for socio-economic position and village urbanisation; and model 3 was further adjusted for lifestyle factors including tobacco and alcohol use, fat and salt consumption, and physical activity level and sedentariness’ (lines 234-241).**

Results:
The authors should not abbreviate 2012 into 12.

**Done. We now use the full form of the years throughout the paper.**

Table 1 must include information stratified by trial arms: intervention arm and control arm.

**Done (Table 1, line 287).**

Table 2 should have a title of the year or year range of these characteristics and then provide a footnote to explain the timepoint was follow-up measures.

**Done. Clarified in title of Table 2 (line 291).**

Table 2 has two columns of the sample sizes for different rows. The authors may better drop this. Then, after placing the maximal numbers of participants of the two arms (949 and 877), create the following two columns. Average % of missing information. Use a superscript to indicate whether the percentages were statistically significantly different between arms. P values for differences between arms.

**Done (Table 2, line 291).**

In Table 2, the authors may better indicate that "Resident village urbanization" and "Standard of living index" were both scores based on some index. Otherwise, "third" would not make sense.

**Done (Table 2, line 291).**

Table and Figure. For each p-value higher than 0.1, present all the values up to the second decimal place.

**Done (in all tables and figures).**

Table S3. CIMT needs an explanation for what it stands for.

**Done (see S5 Table).**

"Table S1-4" is confusing. The authors need to revise this.

**Done. It now reads, S3 Table etc (line 274).**

The authors use "wave 1" or "wave 2 and 3", but it is confusing. Descriptions in text and tables are not consistent (sometimes in "wave" and other times in years), and it is difficult to read. Then, additionally, in Figure 2, the authors state FU1 and FU2/3. All are confusing. The authors should state "baseline", "follow-up 1", and "follow-up 2&3" throughout in text, tables, and figures. "Wave" is not a proper scientific term.
**We have now used the term follow-up (instead of waves) throughout and also referred to the actual timing of the survey in most places to aid clarity.**

Discussion:
The interpretation of Table 4 in comparison to that of Table 3 should include the selection bias during follow-up. The results in the subset are sensitive to the sampling, which is a study-specific factor. The argument about puberty is little convincing, without any biologically plausible, persuasive arguments. The authors are highly encouraged to take out the argument.

**Done. We have taken out the puberty argument and added selection bias (lines 395-403).**

The argument about confounding should be related to the selection of village in regards to the definition of confounding (determinants of exposure). The authors should dig into the selection criteria in details. It is just not convincing (or non-scientific) to speaking about the factors after the intervention as a source of confounding.

**We have now highlighted the possibility of confounding related to selection of villages (lines 337-343). We have now removed puberty as a possible random factor or mediator of findings in the 1^st^ follow-up (please see our response to academic editor on page 1).**

The authors should discuss the lack of blinding in this trial for the participants and the assessors. The reviewer understands it would be difficult to achieve, but the limitation should be available in the text.

**Done (lines 363-366).**

The authors should describe the policy implications that many interventions may be carried out without any apparent effects on cardiovascular phenotypes.

**Done (lines 438-440).**

The lack of assessment of compliance with the intervention seemed to be crucial. The authors' view was too optimistic. The difference by 61 g in offspring's birth weight may have been too small given the absolute levels of birth weights; and also could be by chance, given the history of multiple tests in the prior publications.

**As noted earlier, the 61g birthweight difference is consistent with multiple trials in which compliance was actively monitored. It is also biologically plausible, since birthweight increases within a single generation are constrained by other factors such as maternal size. Birthweight was analysed as a single outcome at the time of original trial (we re-analysed the data later to confirm the difference). On this basis, we do not believe that compliance was a major issue. Nevertheless, we have elaborated on this issue in study limitations (lines 351-358).**

-----------------------------------------------------------
**Requests from the editors:**

Abstract- please provide participant demographics

**Done (lines 36-39).**

Abstract- please provide clear number of years of follow-up reported previously and those reported in the current submission

**Based on comment from the academic editor we have removed reference to previous findings in the introduction (since the trial is not introduced at this stage) and consequently removed it also from abstract. We believe that this makes the abstract and the conclusions clearer. We hope that the editors agree with our judgement; however, we are open to re-introducing the previous study earlier in the text and abstract, if the editors prefer that.**

Please revise for clarity and grammar “The levels of cardiovascular risk factors were generally low” and also avoid the use of vague language like generally.

**Done. We have now stated actual levels of a couple of risk factors (lines 39-40).**

Please provide p-values along with 95% CI throughout and within abstract

**Done.**

Abstract methods and findings-last sentence should be a limitation of your methodology

**Done (lines 46-48).**

Abstract conclusions should begin with “our results show” or similar

**Done (line 50).**

Please place full stop after the reference square brackets

**Done (throughout).**

At this stage, we ask that you include a short, non-technical Author Summary of your research to make findings accessible to a wide audience that includes both scientists and non-scientists. The Author Summary should immediately follow the Abstract in your revised manuscript. This text is subject to editorial change and should be distinct from the scientific abstract. Please see our author guidelines for more information: [https://journals.plos.org/plosmedicine/s/revising-your-manuscript#loc-author-summary](https://protect-eu.mimecast.com/s/CdBsCVQRMU3g49i1nHWR?domain=journals.plos.org)

**Done (lines 60-82).**

Introduction second paragraph- please provide a sentence or two about the previous study before you mention trial and control villages as it is currently not obvious that the previous study was in the same population as in the current submission

**As suggested by the academic editor, we have now removed reference to the findings from the previous follow-up in the introduction since the trial is not yet described. We now refer to these findings in methods and discussion after describing the trial. We believe that the flow and clarity is better as a result; however, we would be happy to re-instate the previous study findings in introduction with details of the trial if the editors wish.**

Was this trial registered? Please provide details, either way within the methods section

**No. We have now added the following statement to methods, ‘****The trial was not registered, as it was not standard practice to register community trials at that time’ (lines 130-131).**

Please provide the study protocol as supplementary information.

**Done (added S2 Text).**

Please revise the reporting in accordance with CONSORT guidelines and provide a completed CONSORT checklist as supplementary information

**Done (added S1 Table).**

Telangana is a large state, please consider providing a map of the villages in both arms to highlight the spread as well as to indicate which areas have undergone urbanisation

**Done (added Fig 1, line 136).**

Please clearly provide names and citations of all surveys, questionnaires used in this study. “Tobacco and alcohol were assessed by standard questions” is not sufficient.

**Done. Added additional citations for surveys (see lines 168, 174, 177).**

Please provide participant demographics in the methods section

**Done (lines 140-142).**

Please provide details of ethics approval and informed written consent in the methods section

**Done (lines 252-257).**

Discussion- please summarise findings in one paragraph, followed by strengths, limitations and comparison to other studies in the literature.

**Done. The discussion section is now thoroughly revised along these lines and other suggestions by reviewers and editors.**

**Comments from the reviewers:
Reviewer #1:** This is a statistical review of manuscript PMEDICINE-D-20-00167_R1. The manuscript reports the results of a long-term follow-up of a cluster trial in nutritional supplementation during pregnancy on offspring's risk of cardiovascular disease.

The statistical methods are appropriate. The reporting is clear, especially thanks to the use of Tables. I only have two minor comments related to Figure 2. Firstly, the text mentions that height was different in adolescence but height isn't reported on Figure 2. Secondly, a legend is missing to distinguish what the blue and red bars represent.

**Done (height and legend added to Fig 2, which is now Fig 3, line 416).**

**Reviewer #2:** This is an interesting manuscript, describing the follow up of young adults born during a village-based nutrition intervention programme in pregnancy and early childhood in Hyderabad, India. It is an important contribution to the DOHaD field.
Specific comments below largely relate to missing detail about the study design, cohort details etc. that prevent the reader from having a full understanding of the cohort.

Abstract
Background: Developmental Origins of Adult Disease Hypothesis is now more commonly called the Developmental Origins of Health and Disease hypothesis. Also in Background, paragraph 1, and Discussion.

**Done (lines 24, 95, 423).**

Insert word, 'control of THE cardiovascular disease epidemic ….'.

**Done (line 26).**

Methods and findings, insert word 'further surveys of THE offspring ….'

**This statement was removed as part of other edits so this does not apply now.**

Define in abstract, is the 1826 70% of the original number of mother-infant dyads randomized? In the method, it states that 2,964 infants were born into the study (or had a birthweight measured) - 1826/2964 = 61%. This isn't clear.

**The cohort size is 2601 children born to 1826 women. The cohort size is smaller than total number of birthweights (N=2964) recorded since the cohort was defined as children born in the trial who were still alive and resident in the study area when the cohort was established in 2003. Those who died or migrated were excluded (details in Figure 1).**

Add some detail to define the low cardiovascular disease risk in the adults (e.g. mean BMI, level of elevated blood pressure).

**Done (lines 39-40).**

Background
Missing word "social disadvantage in THE early years of life ….."

**Done (line 89).**

Methods
Paragraph 2. Further confusion around participant numbers. In paragraph 1, it says that in 2003-5, N=2601 families were contacted (unless I'm misreading this). But in paragraph 2, it states that N=1165 were followed up in 2003-5. Please make this clear.

**Fewer number were examined during first survey due to resource constraints as this was a small-scale PhD project. This was already noted in Methods, but we have now elaborated on it to make it clearer (lines 142-146).**

What was the difference between Wave 2 and Wave 3? Why were there two separate waves, one year apart? Was there any order in how participants were retraced during these waves?

**We have now noted in methods that, ‘The 3rd follow-up was conducted primarily to extend the study to parents and siblings of the trial offspring; however, the trial offspring were also offered the opportunity to take part again, resulting in the close timing of the two surveys’ (lines 149-154). There was no order in how participants were invited, but many participants who attended wave 2 chose not to attend wave 3, and vice versa.**

I note that ten years has passed since wave 2 was complete. Should this be mentioned, e.g. in relation to any further secular trends with respect to CVD risk in India?

**We had already referred to the secular trend in India and the expectation that results may be different in future surveys (lines 369-371, and lines 406-414). We believe that this should suffice.**

Measurements. The reader is referred to two other papers for details on the methodologies used (refs 12 and 14). Ref 12 is the previous follow up study on adolescents, conducted several years earlier. Were all the same interviews used? Is this appropriate, given the differing age/context of the adolescent/young adult groups? Please be specific about which questionnaires/measurements were used as described previously, and which were implemented specifically in this study of adults.

**Methodologies for several measurements such as lifestyle questionnaires, anthropometry, vascular measures, and biochemical assays were similar as they are not age dependent. However, we have now clarified this by providing specific citations for measurements as necessary (see lines 168, 174, 177, 178, 186).**

Weight was measure by digital weighing scales (not machine).

**Done (line 179).**

For anthropometry, I assume measurements were made in duplicate as you indicated that "The mean of two anthropometric measurements …..". But please make this clear. Also, if values were different by <5cm etc. were both values rejected. Or was this at the point of measurement? Please be clear.

**To make clear, the relevant statement now reads, ‘Anthropometric measurements were taken twice and averaged for analyses; where the difference between readings was more than the acceptable level (5mm for height, 0.5kg for weight and 1cm for waist circumference), a third reading was taken’ (lines 182-184).**

Dietary intake data was collected using a semi-quantitative FFQ: "A semi-quantitative food frequency questionnaire coupled with customized nutrient databases was used to estimate average daily salt and fat consumption and energy intake; its validation against multiple weighed 24-hour recalls in this setting has been published.[14]". Only salt and fat intakes have been selected for presentation in the current analysis. I think a justification on this point would be helpful. Also, how are salt intakes estimated from a FFQ? Dietary assessment of sodium intake is unreliable at best, even from a full weighted record. A comment on why these two components of dietary intake have been used in the analysis and their reliability, is warranted. Why not intakes of fruits and vegetables? Or sugar sweetened beverages? Would be interesting to look at other components of diet, given the transitional nature of the population.

**We agree that that the assessment of dietary intake from food frequency questionnaires is not very reliable; hence, we chose to limit the use of dietary variables to a few surrogate markers of unhealthy diet. Salt and fat intake were used as they are established dietary risk factors of cardiovascular disease. Sugar sweetened beverage consumption in the area is still low and fruit and vegetable consumption lacked variability, so they were not included in the analyses. Given the strong null findings in all the models, introduction of additional dietary variables is unlikely to change the results. We believe that a full-scale dietary analysis is beyond the scope of the current publication. We apologise for the incorrect citation for the food frequency questionnaire; the correct citation is now provided, which includes full details on estimation of nutrients (including sodium) from FFQ and their validation against fully weighed records (line 174).**

In addition, Reference 14 is the COHORT profile paper, and does not describe the validation of this tool. Similarly for the statement on physical activity assessment. Please provide the correct, original reference.

**Done (see lines 174, 177).**

Insert word, "AN enzymatic calorimetric method ……"

**Done (line 202).**

Page 13, the method selected for assessing 'village urbanisation' (night-time light intensity) has been shown to be valid in a single paper from China. Are there any other internal modes of validation that could be used from within the existing dataset to demonstrate the robustness of this tool within the Indian context?

**Yes. The tool has been validated against administrative data and cardiovascular risk factors among study participants. These are covered under two separate publications, which are currently submitted to other journals. We have included a statement related to these. We have also provided citation for another paper from APCAPs, which uses a related variable as marker of urbanisation (built-up land use by remote sensing), and also reference for another paper from India that used night-light intensity (lines 216-219 and 406-410).**

Ethics. On Page 12 you describe PPI. Where and how was ethical approval obtained for the follow-up survey described here?

**We have added to the PPI section that, ‘Ethical approvals for second and third follow ups of the cohort reported here were obtained from the Public Health Foundation of India, New Delhi, India; National Institute of Nutrition, Hyderabad, India; University of Bristol, UK; and the London School of Hygiene and Tropical Medicine (LSHTM), London, UK. Approvals were obtained from all village heads and their committees. Study participants provided written informed consent or a witnessed thumbprint if illiterate prior to study start’ (lines 252-257)**

Results.
Table 1. Add sex distribution to Table 1. Is the 62% male follow up reported in Table 2 a similar bias (towards males) as in the original birth cohort and in Wave 1?

**Done. Males represented 52% of original birth cohort and 54% in Wave 1. The lower ratio of girls at 2^nd^ or 3^rd^ follow-up can be explained by young women moving away after marriage and in some cases reluctance to travel outside of village for DXA examination.**

The intent of this sentence is not clear "About 20% of the intervention arm participants lived in the least urbanised third of villages, as opposed to 44% from the control arm." Or rather, I understand the data as presented in Table 2 - but it's not clear if this reflects a bias in rate of urbanisation between the control and intervention villages. I think it would be useful to include this data also in the results - e.g. not participant based, but cluster based.

**Yes, this indicates bias in rate of urbanisation. We have now included cluster based data (lines 266-269).**

What is the distribution of original village clusters according to the new Resident village urbanisation category? Or are we to assume that participants have not moved - so where they live now, is the same village they lived in when born into the study?

**Migration in the villages has been low and invariably to the neighbouring Hyderabad city as opposed to one of the other villages. This is possibly because the villages are close to the city and people can commute to work or education.**

Indeed, in the Discussion, you state that "then the subsequent attenuation of this difference may have resulted from environmental pressure of urbanisation (which has been disproportionately more in the intervention villages)" - but this is not clear from the results. Please modify.
Figure 2. I think it would be more helpful to the reader to replace the axes labels of 'FU1' and 'FU2' with the age of the participants at follow up.

**We speculated this on the basis that urbanisation has been slightly greater in intervention villages. Urbanisation was associated with cardiovascular risk factors in the cohort and effect sizes for some outcomes were slightly attenuated on adjustment for urbanisation. We have now provided age at follow-up in Fig 2 (now Fig 3, see line 416).**

Discussion
An important omission is the MINIMat trial in Bangladesh, which was a trial of early or usual food supplementation (+ additional micronutrients). Cardiovascular disease risk factors have been measured in the offspring.
[https://www.ncbi.nlm.nih.gov/pubmed/23514767](https://protect-eu.mimecast.com/s/bvCaCWqVNfODpGUXI5dr?domain=ncbi.nlm.nih.gov)
[https://www.ncbi.nlm.nih.gov/pubmed/23241449](https://protect-eu.mimecast.com/s/l99gCX5WOI2O60cpeduZ?domain=ncbi.nlm.nih.gov)
Also, an important difference to note between the current Indian trial and those reported from The Gambia and Guatemala (& Bangladesh should be added) is the timing of intervention; India and Guatemala were interventions given in pregnancy and early childhood; The Gambia and Bangladesh supplements were given in pregnancy only. You should add some consideration of the results obtained from the current study, in relation to these other studies. The Discussion on this point is rather cursory.

**The MINIMat trial was not included as we compared our findings to other studies that have published data on cardiovascular risk factors in adulthood, as the long-term significance of such measurements conducted in early childhood is unclear. The above references are for measurements at age 5 years, and so would not meet our criteria. Nevertheless, we have included this citation and also expanded our discussion of other studies as suggested (see lines 372-390).**

I do not follow the statement that "The linkage to additional data collected during the trial (e.g. nutritional supplement collection and maternal and offspring anthropometrics) was not reliable enough to be incorporated in these analyses". It is not clear why linkage to these data is not reliable, yet linkage to other participant details (village of birth, dates of birth) is considered reliable. A greater description on the fidelity of the participant information needs to be included in the methods section of the current paper. Is the data presented on birthweights (reported in both the introduction and discussion, published elsewhere? If so, please include a reference. If not, some further information should be included in the Results of the current paper (birthweight by village, sex).

**The additional data was available in separate electronic files, while key data on participant details was also available in duplicate hard copies against which it could be verified. A greater description of the fidelity of participant information and birthweight data were published in the publication related to earlier follow-up and NIN’s scientific report; we had provided references for both (line 110).**

I also don't understand this statement: "We previously analysed the birthweight data from the trial (birthweights were recorded separately for each village, despite unreliable linkage to study participants) and found a difference of 61g (95%CI 18 to 104g) between trial arms (intervention 2655g, control 2594g), which is comparable to birthweight difference (mean difference 41g, 95% CI: 4.7 to 77.3) reported in a systematic review of trials in which protein-calorie supplementation in pregnancy was directly observed, suggesting that this is a minor concern.[12,13,22]". What is a minor concern? This needs restructuring to make it clear what is meant.

**We have now restructured this to clarify that this refers to the concern that the supplement may have been shared with other family members, thereby reducing the amount received by the intended beneficiary (see lines 348-358).**

Were there any differences in current CVD risk factors according to level of urbanisation of the villages? I believe this could be of value in terms of (i) demonstrating the robustness of the urbanisation tool and (ii) explaining the lack of any associations observed.

**Yes. These data are part of other publications showing strong independent associations between two separate indices of urbanisation measured by remote sensing (built-up land use and night-time light intensity) and CVD risk factors. One of these is now published (which we have now cited) and the other is in late stages of peer review with a journal (we will provide reference as soon as acceptance is confirmed) (lines 406-410).**

**Reviewer #3:** PMEDICINE-D-20-00167R1
Impact of nutritional supplementation in pregnancy on offspring's risk of cardiovascular disease: long-term follow-up of a cluster trial

Thank you for the opportunity to review this manuscript. This is an interesting and compelling revised paper from a strong research team. The authors assessed whether the provision of supplemental nutrition to undernourished pregnant women and their offspring lowered the offspring's risk of cardiovascular disease in young adulthood. The findings of the analyses were null (and the author conducted numerous analyses) but this does not detract from the paper's importance. The authors note that rates of cardiovascular disease tend to increase more rapidly with age in India (more so than in many other countries) and so further follow-up of offspring into middle adulthood may yield different results.

This paper is well written and will be of interest to maternal and child health practitioners and researchers interested in early life origins of adult disease.

Minor comments:

1. The Abstract does not include the primary research question or aim of the study. As written, the research question is not clearly stated but may be gleaned from the middle of the Methods and findings section. The research question is also not clearly stated in the Background section of the manuscript. It would be helpful to include a statement describing the aims of the study just prior to presenting the hypothesis.

**Done (see lines 27-30 and 101-105).**

2. The funding disclosures presented at the beginning and end (within the body) of the manuscript are somewhat inconsistent.

**Done (now consistent).**

3. There are inconsistent decimal places for estimates and Cis in Tables 3 and 4 and p values presented in supplementary tables.

**Done (made consistent throughout).**
