# Supplementary material for: Effect of supplemental nutrition in pregnancy on offspring’s risk of cardiovascular disease in young adulthood: Long-term follow-up of a cluster trial from India
Source: PLoS Med. 2020 Jul 21;17(7):e1003183. doi: 10.1371/journal.pmed.1003183 (PMC7373266; doi:10.1371/journal.pmed.1003183)
Supplement: Attachment — Submitted filename: Response to editors after further revision.docx [file pmed.1003183.s008.docx]

**The Editor**

**PLOS Medicine**

Dear Editor,

Thank you for further comments on our revision. Please see our point by point response to these comments in bold letters below.

Please do not hesitate if you have any follow-on questions or suggestions.

Best wishes,

Sanjay Kinra, on behalf of the co-authors

**Requests from Editors:**

Title- Please add India to the title. Could you please also just say "follow-up of a cluster trial".

**Done. The title now reads, ‘Effect of supplemental nutrition in pregnancy on offspring’s risk of cardiovascular disease in young adulthood: follow-up of a cluster trial from India’.**

The original manuscript document instead of the revised document has been included in the manuscript PDF, please remove the original manuscript copy within editorial manager.

**Done.**

Author summary- please add bullet points

**Done.**

Line 66,67 needs revision, since you are presenting results from a non-randomised study?

**Done. This sentence now reads, ‘Strong evidence for this theory from intervention studies (in which some people are exposed to treatment and others are not) is limited’.**

Lines 75,76- could you revise for clarity? The use of “who” twice is a bit unclear

**Done. The sentence now reads, ‘They found no difference in risk of heart disease between offspring born to women who were offered extra food in pregnancy and childhood those who were not’.**

Author summary lines 78-79 should be tempered, as there are several confounders. Perhaps you can add “our results suggest…” or similar?

**Done. The sentence now reads, ‘Our results showed that extra food offered to undernourished pregnant women may not lower their offspring’s risk of heart disease in young adulthood.’**

Line 80- “cannot” is rather emphatic, please tone down to “should not”. This part of the summary should be revised to indicate that an intervention to the limited time segments (just during a pregnancy period and just after giving birth) was insufficient.

**We agree and thought that even ‘should not’ may come across as too emphatic in some cultures. Therefore, we have revised the statement to say that, ‘****Our findings suggest that, until further evidence is available, policy makers should attach limited value to heart health benefits of food supplementation programs for pregnant women and young children’.**

**For consistency, we have also revised similar statements with the word ‘cannot’ in the main abstract and implications sections (line 426-428). The final sentence in the main abstract now reads, ‘Our findings, coupled with evidence from other intervention studies to date, suggest that policy makers should attach limited value to cardiovascular health benefits of maternal and child protein-calorie food supplementation programs.’**

**We hope that you will agree that these revised statements are less emphatic and capture the uncertainty inherent in limited evidence to date.**

We do not require a patient and public involvement section. However, if you wish to include any of these details within the methods section please do so.

**Done. We have now removed that section and only kept the statements related to ethical approval in the methods (end of second paragraph, lines 159-164).**

Please can you confirm that the included map is free from any copyright restrictions?

**Yes, we confirm.**

Please state within the methods section if any changes were made to the study protocol and provide reasons for the same.

**We have now included the statement at the beginning of the methods section, ‘No change was made to the study protocol after start of the study.’**

Could you please remove page numbers in the CONSORT checklist and replace with section and paragraph numbers. Please also rename the file to state that it is the CONSORT checklist.

**Done.**

Implications
Implications should include the need for monitoring, in general, compliance of program participants with an intervention program. It is possible that a lack of monitoring would make an effective intervention meaningless. This study is in line with it.

**Done. We have now added the following statement to implication section**, **‘Meanwhile, further research involving long-term follow-up of participants of intervention studies in which the compliance is monitored are needed to provide more conclusive evidence.’ Lines 433-435.**

Table 2-The authors should create one more column of p-values from the test comparing intervention groups and control groups for each variable. Or I would suggest revise this column to include p-values from the comparison between two groups. It may be ok to put a table footnote that there was no significant difference in degrees of missing information between the two groups.

**There was a footnote for missing data (*) in Table 2, saying that ‘No significant differences (at p<0.05) in % missing data between trial arms’. Perhaps this would suffice, but please let us know if we have misunderstood this request?**

Village urbanisation score This variable clearly showed the difference between the intervention group and control group. The authors should highlight it.

**Done. We have now edited the following sentence in results section, which now reads**, **‘****The village urbanisation score was clearly different between trial arms: about 20% of the intervention arm participants lived in the least urbanised third of villages, as opposed to 44% in the control arm (3 villages in intervention arm and 7 villages in control arm belonged to the least urbanized thirds category).’ Lines 262-266.**
Table 3- I would suggest to create two columns for Model 1, Model 2, and Model 3 each, putting effect measures in one column and p-values in the other column in each. “p=0.xx”look too messy.

**Done. Also made the same change to Table 4 for consistency.**

Fig3 Bar charts are not good choice. A bar indicates a range from 0 to some positive value. But none of the variables in Fig 3 can be zero. The authors should use diamonds or circules as the best point estimates and 95% confidence intervals.

**Done.**


**Comments from Reviewers:**

Reviewer #1: The authors have satisfactorily addressed my comments.

Reviewer #2: The authors have adequately addressed all the comments made by both myself and the other reviewers and I believe this paper is now suitable for publication.

**We thank both the reviewers.**
